# Origin and Dispersal History of Two Colonial Ascidian Clades in the *Botryllus schlosseri* Species Complex

**DOI:** 10.1371/journal.pone.0169944

**Published:** 2017-01-20

**Authors:** Marie L. Nydam, Kirsten B. Giesbrecht, Emily E. Stephenson

**Affiliations:** Division of Science and Mathematics, Centre College, Danville, Kentucky, United States of America; National Cheng Kung University, TAIWAN

## Abstract

Human-induced global warming and species introductions are rapidly altering the composition and functioning of Earth’s marine ecosystems. Ascidians (Phylum Chordata, Subphylum Tunicata, Class Ascidiacea) are likely to play an increasingly greater role in marine communities. The colonial ascidian *B*. *schlosseri* is a cryptic species complex comprising five genetically divergent clades (A-E). Clade A is a global species, and Clade E has so far been identified in European waters only. Using the largest mitochondrial cytochrome oxidase I datasets yet assembled, we determine the origin and dispersal history of these species. Nucleotide diversity and Approximate Bayesian Computation analyses support a Pacific origin for Clade A, with two likely dispersal scenarios that both show the northwestern Atlantic populations establishing early in the history of the species. Both Discrete Phylogeographic Analysis and Approximate Bayesian Computation support an origin of Clade E on the French side of the English Channel. An unsampled lineage evolved from the French lineage, which reflects the conclusion from the median joining network that not all Clade E lineages have been sampled. This unsampled lineage gave rise to the haplotypes on the English side of the English Channel, which were the ancestors to the Mediterranean and Bay of Biscay populations. Clade E has a wider geographic range than previously thought, and shows evidence of recent range expansion. Both Clade A and Clade E should be considered widespread species: Clade A globally and Clade E within Europe.

## Introduction

Human-induced global warming and species introductions are rapidly altering the composition and functioning of Earth’s marine ecosystems [1–6]. Significant attention is focused on the species that are thriving or will thrive in these new ecosystems in order to predict how these new ecosystems will function [7–12].

Ascidians (Phylum Chordata, Subphylum Tunicata, Class Ascidiacea) are one of the taxa likely to play an increasingly greater role in marine communities [13–14]. The spread of introduced ascidians via shipping traffic [15–27] has accelerated in the last several decades, and is expected to continue [28–29]. Ascidians are often positively impacted by human-induced environmental changes such as increasing water temperatures [30–32], heavy metal pollution [28,33–34], increasing nutrients from sewage [13,35] and sediment runoff [13]. The population growth rate of the colonial ascidian *Botryllus schlosseri* is predicted to increase as ocean temperatures warm [36], and increases in *B*. *schlosseri* density [37–38] and abundance [39] at higher ocean temperatures have been reported. *B*. *schlosseri* is considered to be tolerant of harbor pollution [33].

Despite the central positions that ascidians will occupy in the marine ecosystems of the future, their evolutionary history not well known. In particular, our understanding of ascidian dispersal history is in its infancy [14], largely because of inadequate species identifications [14,40]. Before we can investigate the dispersal patterns of a group, we must establish the species identities and delimitations. Molecular studies in the last two decades have identified many cryptic species complexes of ascidians; groups that appear morphologically indistinguishable but are genetically divergent and/or reproductively isolated [15,24,26–27,41–47]. In cryptic species complexes where genetically divergent clades have been discovered, the origin and dispersal history can be investigated.

The colonial ascidian *B*. *schlosseri* comprises five genetically divergent clades, known as Clades 1–5 [46] and more recently, Clades A-E [26,48]. Clade 1 corresponds to Clade E, Clade 2 to Clade D, Clade 3 to Clade C, Clade 4 to Clade B, and Clade 5 to Clade A. Clade A is documented from the western Mediterranean [26,46], the eastern Mediterranean [49], the southwestern Pacific [50], the northeastern Pacific [26, 51–53], the northwestern Atlantic [26,51–53], the southwestern Atlantic [54] and the northeastern Atlantic [26,46,53]. Clade B has only been found in the Mediterranean Sea (Vilanova, Spain) [46]. Clade C was also found in Vilanova and in one location in the North Atlantic Ocean (Fornelos, Spain) [46]. Clade D was also found in Fornelos, as well as in the English Channel (Roscoff, France) [26,46]. The geographic range of Clade E includes several sites in the Mediterranean Sea [46], two sites on the North Atlantic coast of Spain [46], and several sites in the English Channel [26]. Clades B-E have been termed highly geographically restricted, in contrast to the widespread Clade A [26].

The geographic history of Clade A is unresolved: neither the origin of the species nor the timing and geography of its dispersal routes are clear. The type locality of *B*. *schlosseri* is Falmouth, England [55]. The type specimen is considered to be Clade A [53]. However, both Clade A and Clade E individuals are abundant in Falmouth, England [26]. Only molecular comparisons between Clade A and Clade E individuals have been published; we do not know if they vary in morphology, histology, life history, etc. Individuals from these two clades cannot be discriminated by any superficial morphological characters (e.g. coloration, colony growth morphology) [26]. Therefore, the type specimen from Falmouth could be either Clade A or Clade E. The native population of Clade A has been proposed as the northeastern Pacific [23], the Indo-Pacific [56], the Mediterranean [57,58] or European waters generally [14], but data to confirm these proposals are lacking. A recent paper concludes that Clade A originated in the Mediterranean Sea, based on higher genetic diversity at mtCOI and microsatellites in the Mediterranean Sea than in the North Atlantic coasts of Scandinavia [59].

Although Clade A has spread across the globe, the details of its spread are not well-understood. Given that Clade A and Clade E co-occur in the Mediterranean and the northeastern Atlantic, any records of *B*. *schlosseri* from these areas could be either Clade A or Clade E. *B*. *schlosseri* (Clade A or Clade E) has historically been found in the northeastern Atlantic from southern Norway to northern France and in the Mediterranean, Adriatic and Black Seas [57]. Clade A was first observed in the northwestern Atlantic (New York to Massachusetts, USA) in the mid-1800s [60]. Van Name [61] and Berrill [57] assume that these northwestern Atlantic populations were introduced from Europe via shipping traffic. Yund and co-authors have recently provided evidence that one Clade A haplotype (Bs2) was present in the northwestern Atlantic prior to anthropogenic movement of ascidians via ship transport [53].

Clade A was reported to be widespread (Australia, New Zealand and Japan) in the western Pacific by 1929 [62–63], and was first recorded in the eastern Pacific (San Francisco Bay) in 1944 [64]. Cohen and Carlton [64] consider Clade A to be introduced to the eastern Pacific because neither Van Name [61] nor any other publication prior to 1945 reported this species in this area. Lejeusne et al. conclude that eastern Pacific populations derive from western Pacific populations [19].

Clade E has thus far been identified from six locations in the English Channel [26], six locations from the Western Mediterranean [26,46], and two locations on the North Atlantic coast of Spain [46]. In 11 of these 14 locations Clade E co-occurs with Clade A [26,46]. Because Clade E was not recognized as distinct from Clade A until 10 years ago [46], its origin and dispersal history have not been studied.

Here we use the largest Clade A and Clade E sequence datasets yet assembled to determine the origin and dispersal history of these cryptic species. We examine polymorphism levels and population structure, visualize geographic distribution and frequency of haplotypes using median joining networks, assign probabilities of geographic origins of haplotypes using Bayesian phylogeography, and infer origins and dispersal scenarios using Approximate Bayesian computation.

## Materials and Methods

### Sampling

Individuals were collected from floating docks, buoys and ropes in harbors and marinas. Collection permits are not required in the countries where the individuals were sampled because the sampling locations were not within marine reserves or protected areas and no endangered or protected species were sampled. 337 Clade A individuals were collected from 29 locations in the eastern Pacific, northwestern Atlantic, northeastern Atlantic, and western Mediterranean (S1 Table). The 29 locations are: Ares, Spain; Blanes, Spain; Brest, France; Burela, Spain; Concarneau, France; Estartit, Spain; Falmouth, England; Falmouth MA, USA; Ferrol, Spain; Gijon, Spain; Gosport, England; Hamble Point, England; L’Escala, Spain; Llastres, Spain; Monterey CA, USA; Mutriku, Spain; Parkstone Bay, England; Perros-Guirec, France; Plymouth, England; Poole, England; Quissett MA, USA; Sada, Spain; Sandwich MA, USA; San Sebastian, Spain; Santa Barbara CA, USA; Santander, Spain; Seattle WA, USA; Venice, Italy; Vilanova, Spain.

118 Clade E individuals were collected from 14 locations in the northwestern Atlantic, northeastern Atlantic, and western Mediterranean (S1 Table). The 14 locations are: Blanes, Spain; Brest, France; Concarneau, France; Falmouth, England; Gijon, Spain; Gosport, England; Granville, France; Parkstone Bay, England; Perros-Guirec, France; Plymouth, England; Poole, England; Roscoff, France; Santander, Spain; Torquay, England. Single systems (i.e. groups of ~10 zooids sharing a common exhalant opening) were cut from colonies and placed immediately in a solution of 20% dimethyl sulfoxide (DMSO) saturated with NaCl. Samples were placed at -80°C within 12 days.

### DNA extraction, amplification, sequencing and alignment

Genomic DNA was extracted from whole systems using a Nucleospin Tissue Kit^®^ (Macherey-Nagel, Düren, Germany). Mitochondrial cytochrome oxidase I (mtCOI) was amplified using the universal invertebrate primers LCO1490 and HCO2198 [65]. The mtCOI gene was chosen because it is the gene most often used in previously published phylogeographic studies of *B*. *schlosseri*. PCR amplification was performed in a GenePro Thermal Cycler (Bulldog Bio) using a 10-μl total reaction volume with 2 mM MgCl_2_, 0.2 mM dNTPs, 1 μl of 10x buffer (50mM KCl, 20mM Tris (pH 8.4)), 0.2 μM of each primer, 0.08 U of Taq Polymerase (NEB) and 1 μl of template DNA. The PCR protocol was as follows: 35x (95°C for 30 seconds, 51°C for 1 minute, 72°C for 1 minute). PCR products were incubated with 0.5 μl each of Exonuclease I (New England Biolabs, Ipswich, MA) and Antarctic Phosphatase (New England Biolabs, Ipswich, MA) at 37°C for 45 minutes, followed by 90°C for 10 minutes. The PCR products were sequenced at the University of Kentucky's Advanced Genetic Technologies Center using an ABI-3730 automated sequencer (Applied Biosystems, Foster City, CA).

Sequences from this study were combined with previously published sequences from refs. [19,26,46,52,66], as compiled in [53]. Sequences were aligned using Muscle (MUltiple Sequence Comparison by Log-Expectation) as implemented in the Aligner 6.0.2 software (Codon Code Corporation, Centerville, MA). All sequences in the alignment generated by the authors were manually edited. Sequences with double peaks were occasionally found, which could be evidence of chimerism. These sequences were not included in the alignments. The alignment was trimmed to 524 bp to match the length of previously published sequences. 336 sequences were obtained from the 337 Clade A individuals collected for this study. The Clade A alignment contained a total of 1,085 sequences, 749 from previous studies and 336 from this study. 109 sequences were obtained from the 118 Clade E individuals collected for this study. The Clade E alignment contained a total of 299 sequences, 190 from previous studies and 109 from this study. The sequences from this study are available on Genbank: Accession Numbers JN083237-JN083303 and KX500662-KX500833 for Clade A and KX500834-KX500943 for Clade E.

### Analyses

To assess levels of polymorphism in both clades, the following statistics were calculated in DnaSP 5.10.1 [67]: number of haplotypes, haplotype diversity, nucleotide diversity (π) for all sites, and 2N_e_μ estimated from segregating sites (θ_w_). Only populations with > 3 sequences were included in these calculations.

Population structure within each clade was explored with an analysis of molecular variance (AMOVA), fixation indices (*F*_CT_, *F*_SC_, and *F*_ST_), and pairwise *F*_ST_ values between all populations in Arlequin 3.5.2.2 [68]. Pairwise *F*_ST_ values were corrected for multiple testing using the Benjamini and Yekutieli method [69] in R 3.3.2 [70]. Fixation indices were calculated from distance matrices based on the number of different alleles. For Clade A, the analysis was run twice, with two sets of groups. The first set contained four groups: Mediterranean, northeastern Atlantic, northwestern Atlantic and Pacific. The second set contained seven groups: Bay of Biscay, eastern Pacific, English Channel + Ireland, Mediterranean, North Sea, northwestern Atlantic, and western Pacific. For Clade E, the four groups in this analysis were Bay of Biscay, English Channel England and Ireland, English Channel France, and Mediterranean. The Bay of Biscay groups in our analyses do not perfectly match the geographic boundaries of this body of water. The Bay of Biscay groups contain populations that are 41–56 km west of Cape Ortegal, which is the southwestern boundary of the Bay of Biscay.

To visualize the geographic distribution of Clade A and Clade E haplotypes, median joining networks were produced using PopART 1.7 (Population Analysis with Reticulate Trees) [71]. To assign Clade A and Clade E haplotypes to certain geographic regions of origin, Discrete Phylogeographic Analysis (DPA) was conducted in a Bayesian framework [72]. All of the programs used to perform the DPA are in the BEAST 2 software package [73]. To run this analysis, a BEAST 2 XML file was generated in the program BEAUTi [73]. For Clade A, two separate analyses were run, one with four locations (as in the AMOVA analyses) and one with six (Bay of Biscay, English Channel + Ireland, Mediterranean, North Sea, northwestern Atlantic, and Pacific). For Clade E, a single analysis was completed with four locations, as in the AMOVA. A discrete trait called "location" was defined in the Partitions tab of BEAUTi. The substitution model HKY + I was used, based on JModelTest 2.1.6 [74–75] results. The base frequencies option "estimated" and the relaxed clock lognormal option were selected. The tree prior was Coalescent with Constant Population. The chain length was 2 x 10^8^ generations and was stored every 5 x 10^7^ generations, the trace log every 10^4^ generations, the screen log every 1.2 x 10^6^ generations, the tree log every 10^4^ generations, and the treeWithTraitlogger.location every 10^4^ generations. This BEAUTi-generated XML file was then imported into BEAST 2. The DPA was completed by the BEAST 2 software. The BEAST 2 log file was analyzed in the separate program TRACER [76]. Chain convergence was assessed by viewing trace plots for each parameter and effective sample sizes (ESS) for posterior, likelihood, prior and treeLikelihood statistics in TRACER. Burn-in was assessed by viewing trace plots for each parameter; trees generated in the first 15–30% of the chain were discarded using TreeAnnotator. The output from TreeAnnotator was then sent to the program FigTree 1.4.2 [77]. The phylogenies, along with location posterior probabilities at each node, were visualized using FigTree.

The program DIYABC 2.1.0 [78] was used to investigate the origins and dispersal of Clade A and Clade E. Because mtCOI can be subject to selection [79], which can influence the summary statistics that are central to the DIYABC program, all populations were tested for evidence of selection using Tajima’s D [80] as implemented in DnaSP 5.10.1 [67]. A Tajima’s D value that deviates from zero at α = 0.05 could indicate a demographic process rather than selection, but any populations with a non-zero Tajima’s D were removed from the analysis. For Clade A, these populations were: Bagaduce River, ME; Woods Hole, MA; Falmouth, England (FAL3); Llastres, Spain; New Haven, CT; Ladysmith, BC; and Santa Barbara, CA. For Clade E, one population was excluded: Falmouth, England (FAL2). All populations were also tested for evidence of demographic bottlenecks, using Fu’s Fs [81] and Ramos-Onsins and Rozas’ R2 [82] as implemented in DnaSP 5.10.1 [67] to determine if this scenario should be incorporated into the DIYABC parameters [83]. None of the populations in either Clade A or Clade E showed evidence of a bottleneck event.

For Clade A, populations were grouped into four geographic regions: Mediterranean, northeastern Atlantic, northwestern Atlantic and Pacific. In each of four analyses run in DIYABC, all of the scenarios were constrained to have one of the four geographic regions as the ancestral lineage. For Clade E, populations were grouped into four geographic regions: Bay of Biscay, English Channel England, English Channel France and Mediterranean. Three different analyses were run in DIYABC: English Channel England ancestral, English Channel France ancestral, and Mediterranean ancestral. Results from polymorphism analyses and median joining networks showed Bay of Biscay to be a very unlikely ancestral population.

Prior distributions on effective population size, timing of events, admixture rate and mean mtCOI mutation rate were uniform for both Clade A and Clade E. Minimum and maximum parameter values for the two clades are displayed in S2 Table. For both clades, time parameters were constrained such that t4 > t3, t4 > t2, and t3 ≥ t2.

Every potential demographic scenario for each project was checked in three different ways before it was included in the analysis [83]. Scenarios that did not pass one or more of these checks were not used. First, the “pre-evaluate scenario-prior combinations” option was applied to each scenario, to determine whether the datasets simulated under the scenario and prior conditions matched the observed data set reasonably well. Both PCAs and summary statistics were used to determine the suitability of the scenario and priors for the data set. Second, the “estimate posterior distributions of parameters” option was selected, and then the “Compute bias and precision on parameter estimations” was used to check these parameter estimations. Third, the “Perform model checking” option was applied to each scenario; this option allows the user to determine if the scenario and prior conditions fit the dataset, as summarized by the summary statistics. Both PCAs and summary statistics were used to determine the suitability of the scenario and priors for the data set. After all the checks were complete, reference tables were generated for each project, with 8–15 scenarios in each project. 10^6^ data sets were simulated for each scenario in each project. All scenarios can be found in S3 Table.

After the reference tables were generated, the posterior probabilities of all scenarios in each project were compared using the "Compute posterior probabilities of scenarios" option facilitated by linear discriminant analysis [78]. For each analysis (e.g. Mediterranean ancestral), the scenario with the highest posterior probability was identified. If a single scenario from an ancestral set had the highest probability and its 95% confidence interval (CI) did not overlap with the 95% CI of any other scenario, this single scenario was chosen. If the highest probability scenario overlapped 95% CIs with one or two other high probability scenarios, all of the scenarios with overlapping 95% CIs were chosen. Chosen scenarios could not have 95% CIs that overlapped with any scenarios that were not chosen. Chosen scenarios were then placed in a new analysis for each Clade, which contained the top scenarios from each of the original analyses (e.g. Mediterranean ancestral).

An analysis was then run on each clade; this analysis contained the top scenarios from each original ancestral analysis. Reference tables were generated for the two new analyses (Clade A and Clade E), with 10^6^ simulations for each scenario. Prior values were specified as before. After the reference tables were completed, posterior probabilities of scenarios with linear discriminant analysis were computed, and the scenario with the best posterior probability was identified. This scenario was considered to represent the most likely demographic history for its clade. Finally, the reference tables for the two analyses (Clade A and Clade E) containing the best scenarios were re-generated under a different maximum value for the distribution of effective population size N_e_ (maximum was changed from 2 x 10^5^ to 6 x 10^5^). Posterior probabilities of scenarios were computed, and the top scenarios for N_e_ max = 2 x 10^5^ vs. N_e_ max = 6 x 10^5^ were compared. This was done in order to examine results from more than one N_e_ prior distribution, in the event that variation in prior distribution results in changes to the posterior probabilities of scenarios [84].

## Results

### Clade A polymorphism statistics

In Clade A, 16/68 populations have no polymorphism at mtCOI; there is no discernible geographic clustering of these populations (S4 Table). Three populations have haplotype diversity values ≥ 0.8, two from the northwestern Atlantic and one from Bay of Biscay. The highest π value is in Bodega Bay, CA (0.1916), which is nearly 10x higher than the nucleotide diversity in any other population. The two populations with the highest θ_w_ values are in the northwestern Atlantic (Port La Tour, Nova Scotia and Nahant, Massachusetts).

When populations are split into four groups (Mediterranean, northeastern Atlantic, northwestern Atlantic, Pacific), nucleotide diversity is highest in the Pacific, and lowest in the northeastern Atlantic. When populations are split into six groups by replacing the northeastern Atlantic populations with Bay of Biscay, English Channel + Ireland, and North Sea, nucleotide diversity is highest in the Pacific, and lowest in the Bay of Biscay.

### Clade A population structure

AMOVA results are very similar whether four or seven geographic regions were used, so only results from four geographic regions will be reported (Table 1). More of the variation is found within populations (42%) than among groups (31%) or among populations within groups (27%). Population pairwise *F*_st_ values are high (S5 Table). The highest average pairwise *F*_st_ values are between the Pacific group and the other three groups (0.604–0.629) (S6 Table). The average pairwise *F*_st_ values between the non-Pacific groups range from 0.49 (Mediterranean vs. northeastern Atlantic) to 0.551 (Mediterranean vs. northwestern Atlantic).

**Table 1:** Structure of populations within Clade A as determined by AMOVA.

Source of variation	df	Sum of Squares	Variance components	Percentage of variation
Among groups	3	123.725	0.14546	30.71
Among population within groups	80	143.836	0.12799	27.02
Within populations	1001	200.414	0.20021	42.27
Fixation Indices	Value	P-value		
*F*_CT_	0.38998	< 0.001 ± 0		
*F*_SC_	0.57731	< 0.001 ± 0		
*F*_ST_	0.30709	< 0.001 ± 0		

Groups: Mediterranean, northwestern Atlantic, northeastern Atlantic, and Pacific

Differentiation among populations is significant both among populations within groups (*F*_sc_) and among populations among groups (*F*_st_), as is differentiation among groups (*F*_ct_) (Table 1). All P-values for the hierarchical F-statistics are < 0.001. The two populations with the highest number of statistically significant pairwise *F*_st_ values are Graña, Spain (northeastern Atlantic) and Bodega Bay, CA, USA (Pacific). The three populations with the fewest statistically significant pairwise *F*_st_ values are Saint Marie de la Mer, France (Mediterranean), Gosport, England (northeastern Atlantic) and Port Pendennis, England (northeastern Atlantic).

### Clade A haplotypes

35 haplotypes are present in the 1,085 sequences (S7 Table). 18 haplotypes were published in previous studies [19,26,46,66] and 17 haplotypes have not previously been published. The new haplotypes are Bs39-Bs55. These labels follow the naming conventions used in recent *B*. *schlosseri* publications [19,26,46,66]. The haplotype designation of all sequences obtained for this study can be found in S8 Table.

### Clade A haplotype distribution

**Fig 1:**
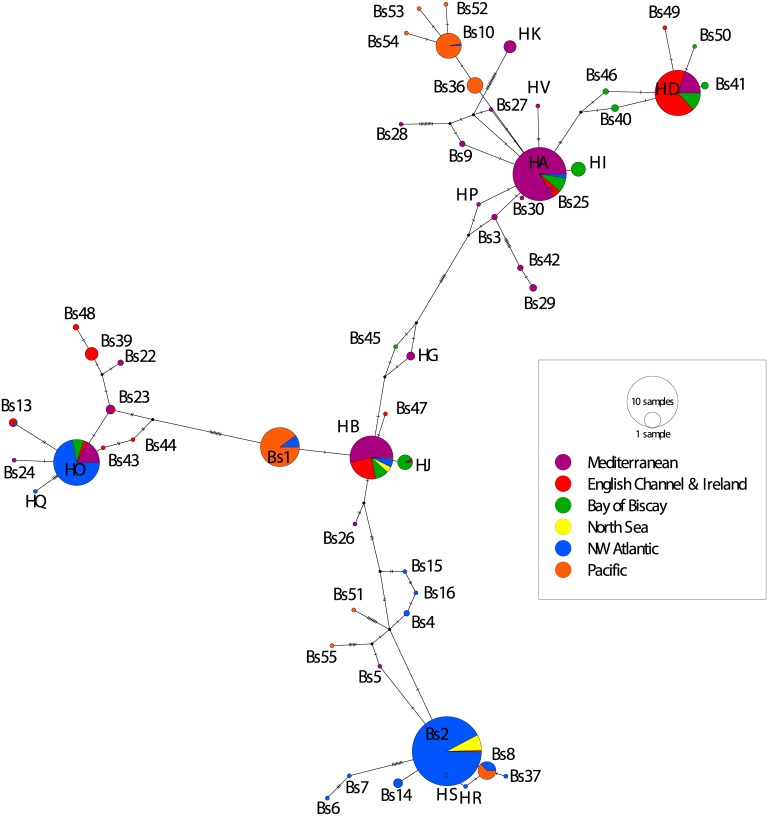
Median joining haplotype network of all Clade A populations. Populations are grouped into six geographic regions: Mediterranean, English Channel + Ireland, Bay of Biscay, North Sea, northwestern (NW) Atlantic, Pacific.

Median joining networks using 4 and 6 geographic locations (S1 Fig and Fig 1, respectively) show the major haplotype groups: HA, HB, HK, HO, Bs1, Bs2, and Bs10/Bs36. The HA haplotype group contains Bs3, Bs9, Bs25, Bs27, Bs29, Bs30, Bs36, Bs40, Bs41, Bs42, Bs46, Bs49, Bs50, Bs52, Bs53, Bs54, HA, HD, HI, HP, and HV. The HB haplotype group contains Bs26, Bs45, HB, HG, and HJ. The HK haplotype group contains Bs28 and HK. The HO haplotype group contains Bs13, Bs22, Bs23, Bs24, Bs39, Bs43, Bs44, Bs48, HO, and HQ. The Bs1 haplotype group contains only the Bs1 haplotype. The Bs2 haplotype group contains Bs2, Bs4, Bs5, Bs6, Bs7, Bs8, Bs14, Bs15, Bs16, Bs37, Bs51, Bs55, HR, and HS. The Bs10/Bs36 haplotype group contains Bs10, Bs36, Bs52, Bs53, and Bs54.

**Fig 2:**
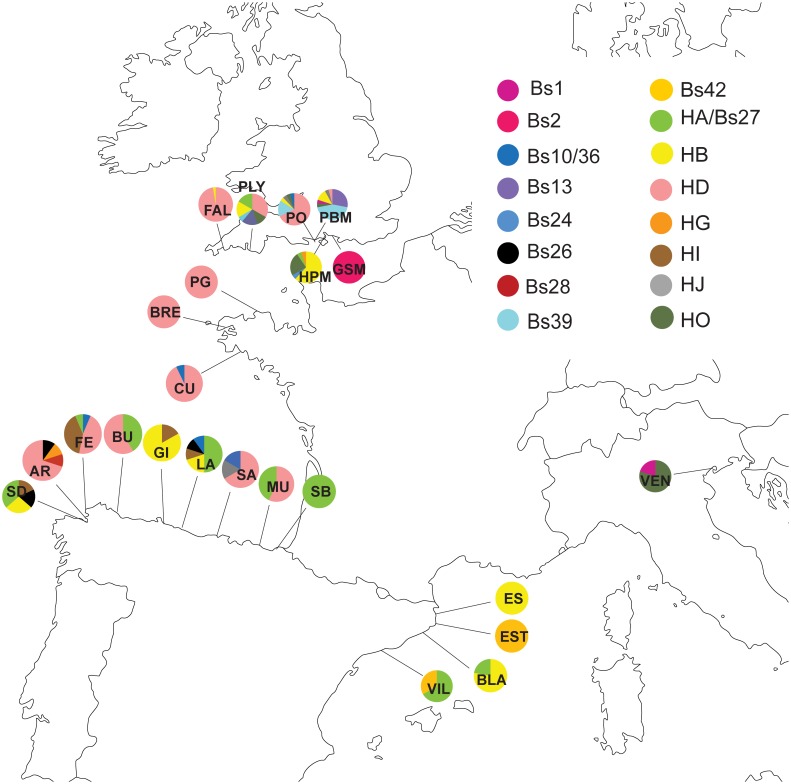
Clade A haplotype frequencies in the northeastern Atlantic populations sampled by the authors of this study. The population abbreviations are as follows: AR = Ares, Spain; BLA = Blanes, Spain; BRE = Brest, France; BU = Burela, Spain; CU = Concarneau, France; ES = L'Escala, Spain; EST = Estartit, Spain; FAL = Falmouth, England; FE = Ferrol, Spain; GI = Gijon, Spain; GSM = Gosport, England; HPM: Hamble, England; LA = Llastres, Spain; MU = Mutriku, Spain; PBM = Parkstone Bay, England; PG = Perros-Guirec, France; PLY = Plymouth, England; PO = Poole, England, SA = Santander; SB = San Sebastian, Spain; SD = Sada, Spain; VEN = Venice, Italy; VIL = Vilanova, Spain.

The HA haplotype group is primarily Mediterranean in composition, but there are also individuals from this haplotype group in the English Channel, the Bay of Biscay, and the northwestern Atlantic. HA is one of three common haplotypes in the Mediterranean and one of six common haplotypes in the Bay of Biscay, but it is not common in the English Channel (Fig 2). Haplotype frequencies for all Clade A populations are listed in S9 Table.

The HA haplotype group contains the HD haplotype, which is primarily English Channel + Ireland but also contains samples from the Bay of Biscay and the Mediterranean. The HD haplotype dominates the English Channel coast of France. The HD haplotype is common on the English Channel coast of England and in the Bay of Biscay (Fig 2 and S8 Table). The HD haplotype was found in a single Mediterranean location (Cadaqués, Spain).

**Fig 3:**
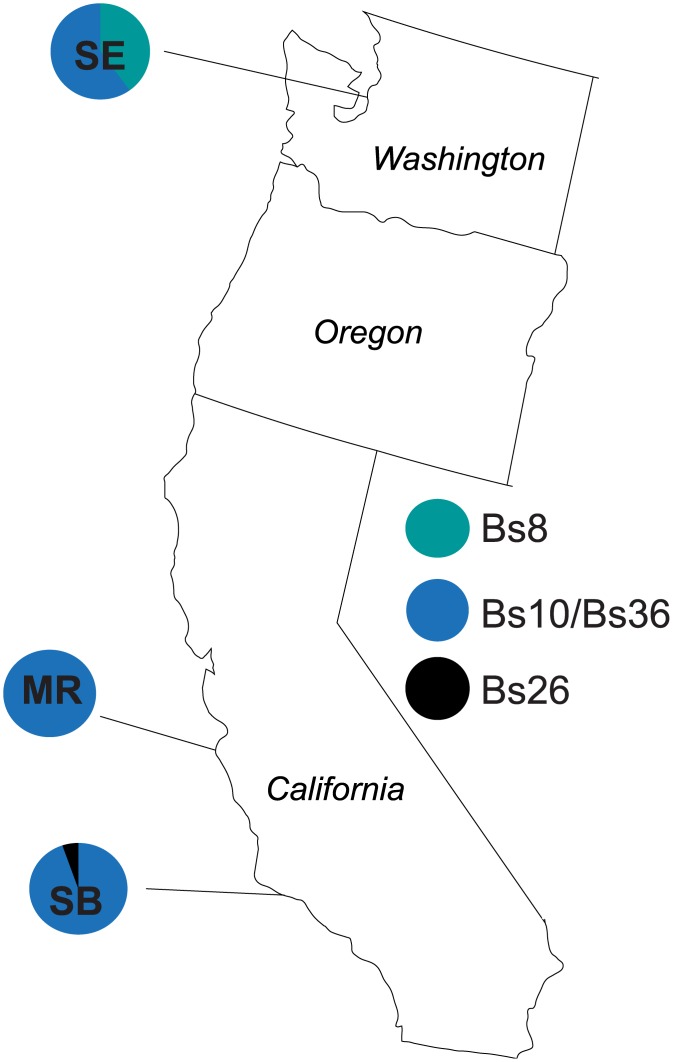
Clade A haplotype frequencies in the eastern Pacific populations sampled by the authors of this study. The population abbreviations are as follows: MR = Monterey, CA, USA; SB = Santa Barbara, CA, USA; SE = Seattle, WA, USA. Small cross-marks represent mutational steps.

The HA haplotype group is also closely connected to the Bs10/Bs36 haplotype group, which is almost entirely composed of individuals living in the Pacific Ocean. The Bs10/Bs36 haplotype is dominant in the three eastern Pacific populations sampled for this study (Fig 3).

The HA haplotype group is connected to the HB haplotype group, although there are 10 mutations separating the two groups. All six geographic locations have individuals belonging to the HB haplotype group. Over half of the individuals in HB haplotype group are found in the Mediterranean, with ~25% in the English Channel and Ireland. Smaller percentages are found in the North Sea, the Bay of Biscay, the northwestern Atlantic and the Pacific. HB is the most common haplotype in the Mediterranean (Fig 2). The HB haplotype is common in the Bay of Biscay and the English Channel coast of England, although it never represents more than 50% of two populations in these areas.

**Fig 4:**
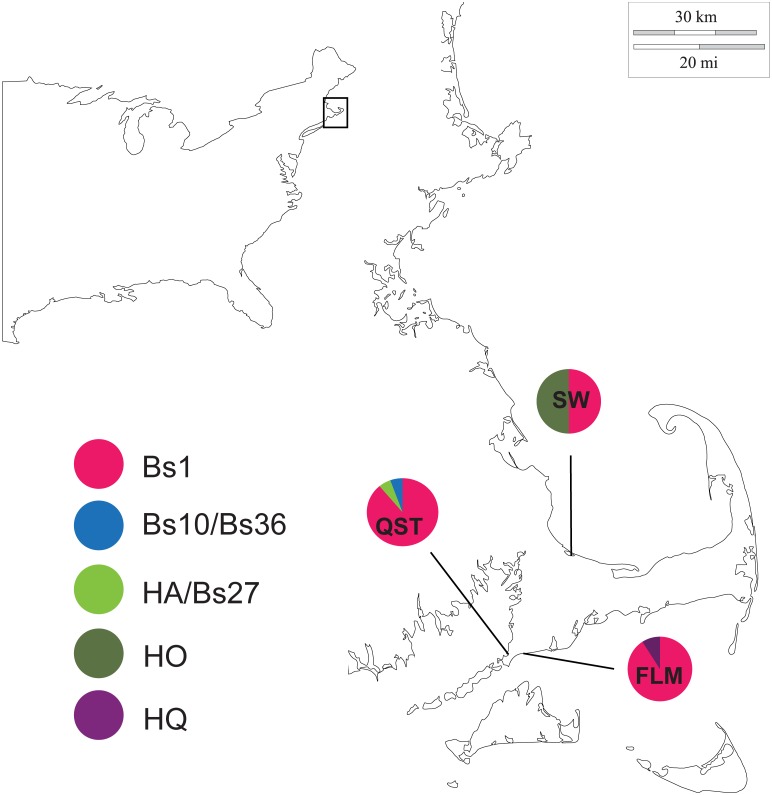
Clade A haplotype frequencies in the northwestern Atlantic populations sampled by the authors of this study. The population abbreviations are as follows: FLM = Falmouth, MA, USA; QST = Quissett, MA, USA; SW = Sandwich, MA, USA.

The HB haplotype group is only one mutational step away from the Bs1 haplotype group, which is primarily Pacific with a small percentage of northwestern Atlantic individuals. However, Bs1 is the most common haplotype in the northwestern Atlantic populations sampled for the first time in this study (Fig 4). The Bs1 haplotype is not present in the Eastern Pacific sequences obtained for this study (Fig 3); all of the Bs1 haplotypes are found in the Western Pacific (S7 Table).

Nine mutational steps from the Bs1 haplotype group is the HO haplotype group, 70% of which is found in the northwestern Atlantic. Smaller percentages are found in the Bay of Biscay, English Channel and Ireland, and the Mediterranean.

The last major haplotype group, Bs2, has the HB haplotype group as its nearest neighbor. The Bs2 haplotype group is primarily from the northwestern Atlantic region, with a small number of individuals from the North Sea. The Bs2 haplotype was not found in the Mediterranean or northeastern Atlantic (Figs 1 and 2).

The population-level patterns for Clade A in the Pacific and northwestern Atlantic are thoroughly described by [53], but the present study adds 17 populations to the northeastern Atlantic (Bay of Biscay and both sides of the English Channel) and adds sequences to six previously sampled locations in the northeastern Atlantic, including the type locality of *B*. *schlosseri*: Falmouth, England [55]. Within the northeastern Atlantic, the Spanish Mediterranean populations share haplotypes, with each of three haplotypes (HA/Bs27, HB, Bs42) present in two populations (Fig 1). Each of these three haplotypes is genetically distinct, however. The Italian Mediterranean population (Venice) does not share haplotypes with the Spanish Mediterranean populations. The Venice haplotypes (HO and Bs1) are widespread and found in several geographic regions; HO is most common in the northeastern Atlantic and Bs1 in the Pacific.

The Bay of Biscay populations share haplotypes, with each population sharing at least one of its haplotypes with another population (Fig 2). Santander contains the haplotype HJ, which was also found in the Bay of Biscay (Graña) by [46]. Haplotypes HG and Bs28 are found in Ares, Spain. Neither of these haplotypes is found elsewhere in the Bay of Biscay, but both are also found in the Spanish Mediterranean [26,46].

Of the nine populations in the English Channel, six have HD as the dominant haplotype (Fig 2). The French populations are almost entirely composed of HD haplotype individuals, as is Falmouth on the English side. The other English populations (Hamble Point, Parkstone Bay, Plymouth, and Poole) contain individuals from 5–7 different haplotypes. These haplotypes are largely shared between populations, although haplotype HG is only seen in Hamble Point, and haplotype Bs1 is only seen in Parkstone Bay.

### Clade A origins

The DPA specifying four locations produced the location posterior probabilities in Table 2 and S10 Table. Only the posterior probabilities for the major haplotype groups (Bs2, HB, Bs1, HO, HK, HA, Bs10/Bs36; S1 Fig) are shown.

**Table 2:** Location posterior probabilities for Clade A haplotype groups.

Haplotype group	Mediterranean	NE Atlantic	NW Atlantic	Pacific
Bs2	0.0411	0.0802	0.6102	0.2685
HB	0.5102	0.449	0.0408	0.0000
Bs1	0.0019	0.0013	0.0154	0.9814
HO	0.3117	0.4672	0.198	0.0231
HK	0.626	0.3062	0.0514	0.0164
HA	0.3979	0.3929	0.143	0.0662
Bs10/Bs36	0.0000	0.0004	0.0027	0.997

Populations are grouped into four geographic regions.

The DPA specifying six locations produced the location posterior probabilities in Table 3 and S10 Table. Only the location posterior probabilities for the major haplotype groups (Fig 1) are shown. As in the four locations analysis, the northwestern Atlantic is the likely origin of the Bs2 haplotype group (posterior probability = 0.56). The Bs1 haplotype group, with a posterior probability of 0.99, almost certainly originated in the Pacific. The Mediterranean is the most likely origin for the HB haplotype group. This is in contrast to the findings of the four locations DPA of the HB haplotype group, which has an equal probability of originating in the Mediterranean and the northeastern Atlantic. When the northeastern Atlantic is split into three separate groups in the six locations analysis, none of them are identified as more probable than the Mediterranean. The HO haplotype group could have originated in the English Channel + Ireland or the Mediterranean. In the four locations analysis, the likeliest origins were northeastern Atlantic or the Mediterranean; it appears that the most probable specific location inside the northeastern Atlantic is the English Channel + Ireland rather than the North Sea or the Bay of Biscay. The HK haplotype group originated in the Mediterranean according to both the four and six locations analyses. The HA haplotype group has a roughly equal probability of originating in the Mediterranean (0.29), the English Channel + Ireland (0.23), and the Bay of Biscay (0.28). This is a similar result to the four locations analysis. Finally, the Bs10/Bs36 haplotype group originated in the Pacific.

**Table 3:** Location posterior probabilities for Clade A haplotype groups.

Haplotype group	Mediterranean	English Channel + Ireland	Bay of Biscay	North Sea	NW Atlantic	Pacific
Bs2	0.0249	0.0374	0.136	0.0237	0.5612	0.3392
HB	0.4098	0.238	0.1691	0.0117	0.140	0.0315
Bs1	0.0006	0.0016	0.001	0.0009	0.0104	0.9855
HO	0.2859	0.3576	0.112	0.0131	0.205	0.0264
HK	0.5063	0.194	0.2462	0.0094	0.0341	0.0101
HA	0.2891	0.234	0.2757	0.034	0.1019	0.0563
Bs10/Bs36	0.0001	0.0001	0.0001	0.0002	0.0014	0.9984

Populations are grouped into six geographic regions.

For the DIYABC analysis, the most likelihood scenario (or scenarios) for each ancestral region are presented in S11 Table. In the Mediterranean ancestral analysis, the posterior probability for the most likely scenario (Scenario 2) was low. The next most likely scenario (Scenario 1) was therefore also included in the next round of analysis, even though the 95% CIs of the posterior probabilities for these two scenarios did not overlap. The Pacific ancestral analysis contained two scenarios (21 and 26) with very similar posterior probabilities and broadly overlapping 95% CIs: both of these scenarios were included in the analysis that compared the best scenarios from all ancestral analyses. The northeastern Atlantic ancestral project had a single best scenario (14). The northwestern Atlantic ancestral project contained three scenarios (15, 48 and 55) with close posterior probability values and broadly overlapping CIs: all three of these scenarios were included in the next round of analysis.

All 8 scenarios from the ancestral sets (Scenarios 1,2,14,15,21,26,48,55) were compared in two separate analyses: one where the maximum N_e_ was 2 x 10^5^ and another where the maximum N_e_ was 6x10^5^. Regardless of which maximum N_e_ was used, three scenarios had much higher posterior probabilities than the other five scenarios (Table 4). These three scenarios (21, 26, 48) had broadly overlapping 95% CIs, so they could not be differentiated. Therefore, these scenarios were compared to each other. The scenario with the highest probability was Scenario 26 when the maximum N_e_ was 2 x 10^5^ and Scenario 21 when the maximum N_e_ was 6 x 10^5^ (Table 5). Both scenarios assign the Pacific region as the origin of Clade A (S3 Table). Scenario 26 shows the Mediterranean lineage descending from the ancestral Pacific lineage early in the evolutionary history of Clade A. This Mediterranean lineage gives rise to a northwestern Atlantic lineage and a northeastern Atlantic lineage (S3 Table). In Scenario 21, an unsampled lineage diverges from the original Pacific lineage. Two Atlantic lineages emerge from the unsampled lineage: a northwestern Atlantic and a northeastern Atlantic lineage. Finally, the Mediterranean populations arise from the northeastern Atlantic lineage.

**Table 4:** Posterior probabilities and 95% confidence intervals (CI) for the most likely dispersal history scenarios when the best scenarios from each ancestral set are compared against each other.

	Scenario #	PP 2x10^5^	95% CI	PP 6x10^5^	95% CI
Mediterranean ancestral	1	0.0259	0–0.0517	0.0688	0.0553–0.0823
	2	0.0267	0.0007–0.0526	0.0813	0.0677–0.0948
NE Atlantic ancestral	14	0.0408	0.0109–0.0707	0.0956	0.0820–0.1093
NW Atlantic ancestral	15	0.0399	0.0145–0.0652	0.0785	0.0606–0.0963
	48	0.2513	0.2283–0.2743	0.1735	0.1546–0.1925
	55	0.1015	0.0810–0.1219	0.1302	0.1150–0.1454
Pacific ancestral	21	0.2483	0.2266–0.2700	0.2031	0.1821–0.2242
	26	0.2657	0.2429–0.2886	0.1689	0.1512–0.1866

Results from both maximum N_e_ max = 2 x 10^5^ and maximum N_e_ = 6 x 10^5^ are presented. PP = posterior probability of each scenario.

**Table 5:** Posterior probabilities and 95% confidence intervals (CI) for the most likely dispersal history scenarios when three equally likely scenarios (21, 26, 48) were compared against each other.

	Scenario #	PP 2x10^5^	95% CI	PP 6x10^5^	95% CI
NW Atlantic ancestral	48	0.2433	0.2318–0.2548	0.0688	0.0294–0.1082
Pacific ancestral	21	0.3569	0.3485–0.3653	0.5042	0.4900–0.5184
	26	0.399	0.3911–0.4084	0.427	0.4125–0.4415

Results from both maximum N_e_ max = 2 x 10^5^ and maximum N_e_ = 6 x 10^5^ are presented.

### Clade E polymorphism statistics

The number of haplotypes is positively correlated with the sample size (S4 Table). Single haplotypes were found in Canet, France (8 individuals) and Fornelos, Spain (9 individuals), whereas 7 haplotypes were found in Plymouth, England (41 individuals). Haplotype diversity ranges widely, from 0 in Canet, France and Fornelos, Spain to 0.842 in Brixham, England. The population with the highest π and θ_w_ values is Sete, France (in the Mediterranean). As a group, the Mediterranean populations have the highest polymorphism levels (roughly double that of the group with the next highest levels: English Channel England). The Bay of Biscay populations have the lowest polymorphism levels.

### Clade E population structure

A majority (66%) of the variation was found within populations, with 22% among populations within groups and 12% among groups (Table 6). Population pairwise *F*_ST_ values are high (S5 Table). The highest average pairwise *F*_ST_ is between the Mediterranean and Bay of Biscay groups (0.795), and the lowest is between the two English Channel groups (0.236) (S6 Table). Pairwise *F*_ST_ values between the Mediterranean and the two English Channel groups and between the Bay of Biscay and the two English Channel groups are intermediate (0.426–0.581).

**Table 6:** Structure of populations within Clade E as determined by AMOVA.

Source of variation	df	Sum of Squares	Variance components	Percentage of variation
Among groups	3	14.958	0.05145	11.88
Among population within groups	23	32.957	0.09653	22.3
Within populations	313	89.185	0.28494	65.82
Fixation Indices	Value	P-value		
*F*_CT_	0.11884	< 0.001 ± 0		
*F*_SC_	0.25306	< 0.001 ± 0		
*F*_ST_	0.34182	< 0.001 ± 0		

Groups are Bay of Biscay, English Channel England and Ireland, English Channel France, and Mediterranean.

Differentiation among populations is significant both among populations within groups (*F*_SC_) and among populations among groups (*F*_ST_), as is differentiation among groups (*F*_CT_) (Table 6). All P-values for the hierarchical *F* statistics are < 0.001. Each population has significant pairwise *F*_ST_ values in comparisons with 9–26 other populations. The population with the fewest significant pairwise *F*_ST_ values (2) is Granville, France (English Channel coast of France). The population with the most significant pairwise *F*_ST_ values (19) is Canet, France (Mediterranean coast of France).

### Clade E haplotypes

There are 23 haplotypes contained in 299 sequences (S7 Table). 17 haplotypes have been published previously [19, 26, 46, 53]. The new haplotypes are Bs56-Bs61. The haplotype designation of all sequences used in this study (including previously published sequences) can be found in S8 Table.

### Clade E haplotype distribution

**Fig 5:**
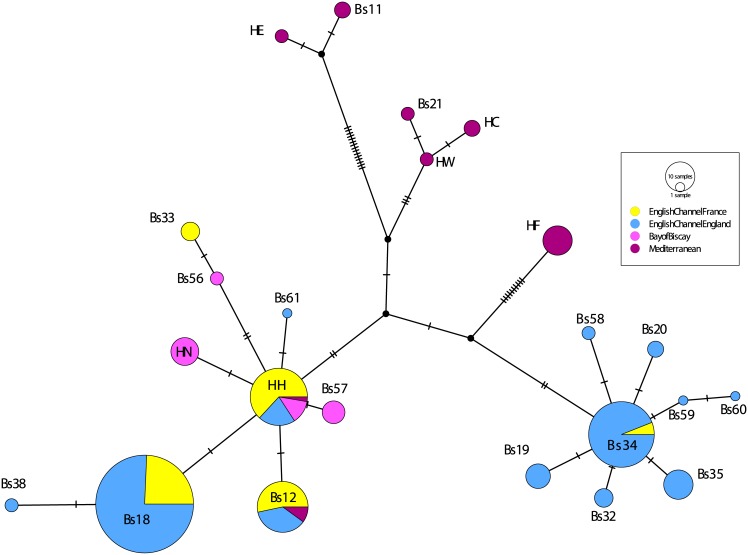
Median joining haplotype network of all Clade E populations. Populations are grouped into four geographic regions: English Channel France, English Channel England, Bay of Biscay, and Mediterranean.

**Fig 6:**
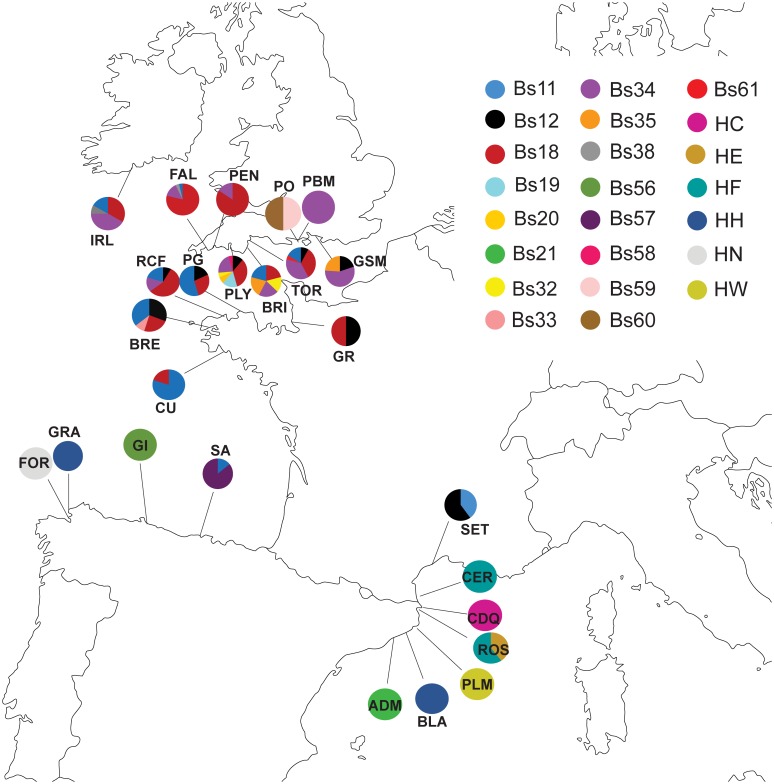
Clade E haplotype frequencies in the northeastern Atlantic and Mediterranean populations by the authors of this study.

The center of the Clade E median joining network is populated by distinct Mediterranean-endemic haplotypes which are not common in this data set (HC, HE, HF, HW, Bs11 and Bs21) (Fig 5). Haplotypes HC, HE, HW, Bs11 and Bs21 are only present in one population each. Cadaqués (HC), Palamos (HW), Arenys de Mar (Bs21) have no other haplotypes, but < 3 individuals were collected from each of these populations (S1 Table). Roses, Spain (HE) also contains HF haplotype individuals, and Sete, France (Bs11) also contains the more widespread haplotype Bs12 (S8 Table). Haplotype HF can be found in two neighboring populations: Roses, Spain and Canet, France (Fig 6). Haplotype frequencies are listed in S9 Table.

The Mediterranean haplotypes are connected to unsampled haplotypes, represented by black circles at nodes (Fig 5). These unsampled haplotypes are closely connected to the major haplotype group Bs34. The Bs34 haplotype is primarily located in the English side of the English Channel, with a small proportion found on the French side of the English Channel (Fig 6). The Bs34 haplotype makes up 12–56% of the English coast of the English Channel populations where it occurs, and 19% of the only population on the French coast of the English Channel where it is found (Roscoff, France) (S8 Table).

The unsampled nodes are also closely connected to the haplotype HH. Haplotype HH was originally sequenced from a single location in the Bay of Biscay by [46] (S7 Table). However, a wider sampling effort shows that haplotype HH is geographically diverse. A majority of individuals with the HH haplotype are found on the French side of the English Channel, but substantial percentages are also found on the English side of the English Channel, Ireland, and in the Bay of Biscay. HH haplotype individuals comprise 24–80% of the French English Channel populations where they are found (S8 Table). A very small number of HH haplotype individuals are located in the Mediterranean (Fig 6).

The Bs12 haplotype is only a single mutational step away from the HH haplotype, and has sequences from both sides of the English Channel and the Mediterranean. The Bs18 haplotype is also only a single mutational step away from the HH haplotype, and is present only in the English Channel (75% of the individuals are on the English side, 25% on the French side) (S8 Table and Fig 6).

Fig 6 shows several interesting population-level patterns. In addition to the Mediterranean endemism highlighted in Fig 5, the Mediterranean region is genetically diverse, with only Roses, Spain and Canet, France sharing haplotypes. The Bay of Biscay region is also diverse with a high level of endemism: three of the four locations sampled have haplotypes found only at that location (Fornelos: HN, Gijon: Bs56, and Santander: Bs57) (Fig 6). All three of these endemic haplotypes are genetically similar to haplotype HH (Fig 5). Since haplotype HH is also present in the Bay of Biscay, HN, Bs56 and Bs57 could have evolved from this abundant and widespread haplotype.

Moving to the English Channel, the genetic composition of the French populations is largely homogeneous, and similar to the English populations. The exception is haplotype Bs33, which appears to be endemic to Brest, France (Fig 6). The geography of this haplotype is unexpected, given that it is derived from Bs56, a haplotype restricted to single population in the Bay of Biscay (Fig 5). On the English side of the English Channel, haplotypes are shared between populations, with exceptions in Plymouth and Poole, England. These populations contain several endemic haplotypes derived from haplotype Bs34 (Fig 5), which is common and widespread on the English coast of the English Channel (Fig 6).

### Clade E origins

Location posterior probabilities produced by the DPA are shown in Table 7 and S10 Table. Only the location posterior probabilities for the major haplotype groups (HE, HF, HW, Bs34, HH, Bs12, Bs18; Fig 5) are shown. The HE haplotype group is composed of HE and Bs11. The HF haplotype group contains only HF. The HW haplotype group includes HC, HW and Bs21. The Bs34 haplotype group is composed of Bs19, Bs20, Bs32, Bs34, Bs35, Bs58, Bs59, and Bs60. The HH haplotype group contains Bs33, Bs56, Bs57, Bs61 and HN. The Bs12 haplotype group includes Bs12 only. The Bs18 haplotype group is composed of Bs18 and Bs38.

**Table 7:** Location posterior probabilities for Clade E haplotype groups.

Haplotype groups	English Channel France	English Channel England	Bay of Biscay	Mediterranean
HE	0.022	0.0035	0.0091	0.9854
HF	0.0031	0.0001	0.0007	0.9862
HW	0.0091	0.0006	0.0033	0.987
Bs34	0.0444	0.9506	0.0027	0.0024
HH	0.7613	0.2271	0.0093	0.0023
Bs12	0.7148	0.2743	0.0058	0.0051
Bs18	0.1622	0.8346	0.0027	0.0006

For the DIYABC analyses, the Mediterranean ancestral analysis contained two scenarios (7 and 8) with high posterior probabilities; both of these scenarios were included in the analysis comparing the best scenarios from across each ancestral set (S11 Table). The English Channel England ancestral project had a single likely scenario (15) with a posterior probability value of 0.5288. The English Channel France ancestral project had a single highly likely scenario (19) with a posterior probability value of 0.8699.

When all four best scenarios from the ancestral projects (Scenarios 7, 8, 15 and 19) were compared together, Scenario 19 was the most likely (Table 8). Results from maximum N_e_ = 2 x 10^5^ are shown in Table 8; results from maximum N_e_ = 6 x 10^5^ are very similar (Scenario 19 posterior probability: 0.9838, 95% CI: 0.9796–0.9879). In Scenario 19, Clade E originated on the French side of the English Channel (S3 Table). Then, an unsampled population diverged from the ancestral Clade E population. This unsampled population gave rise to the populations on the English side of the English Channel, which in turn gave rise to the Mediterranean and Bay of Biscay populations.

**Table 8:** Posterior probabilities and 95% confidence intervals (CI) for the most likely Clade E dispersal scenarios, when the best scenarios from each ancestral set are compared against each other.

	Scenario #	Posterior Prob.	95% CI
Mediterranean ancestral	7	0.0003	0.0000–0.4730
	8	0.0003	0.0002–0.0005
English Channel England ancestral	15	0	0.0000–0.4727
English Channel France ancestral	19	0.9994	0.9991–0.9997

### Clade A and Clade E geographic overlap

**Fig 7:**
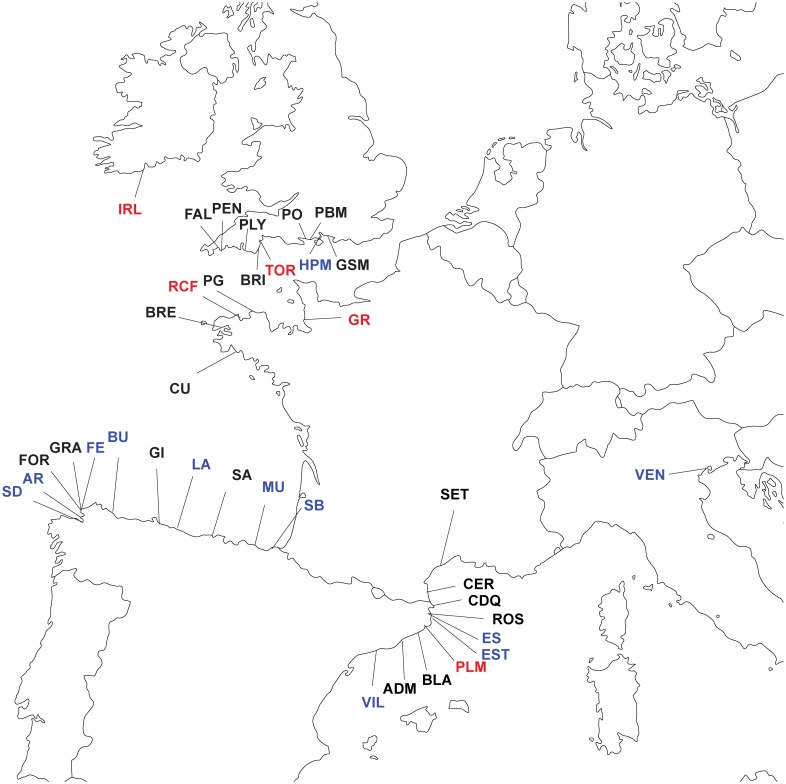
Distribution of Clade A and Clade E throughout the northeastern Atlantic and Mediterranean. Blue Labels = Locations where only Clade A was found, Red Labels = Locations where only Clade E was found, Black = Clade A and Clade E sympatric.

Fig 7 shows the geographic distribution of Clades A and E in the northeastern Atlantic and Mediterranean region. Clade E is not found outside of this region. Clade E is sympatric with Clade A across its entire geographical range, and rarely occurs in allopatry. There are 5 locations where only Clade E has been collected: Palamos (Spain), Roscoff and Granville (France), Torquay (England) and Cobh (Ireland). Several of these locations may have been incompletely sampled: only one individual was sampled from Palamos, and only two individuals from Granville (S1 Table). Clade A is almost always sympatric with Clade E in the English Channel (Hamble Point, England being the single exception); this is likely due to Clade E’s greater abundance in the English Channel than in other geographic regions in the northeastern Atlantic and Mediterranean. Clade A is sympatric with Clade E in 6/10 Spanish Mediterranean sites, and two of the remaining sites (Palamos and L'Escala) were not sampled extensively (S1 Table). Clade A is more often found without Clade E than with Clade E in the Bay of Biscay; this is due to the low abundance of Clade E in this region.

## Discussion

### Geographic history of Clade A

The highest nucleotide diversity levels in Clade A are found in the Pacific. This suggests a Pacific origin for Clade A, which is corroborated by DIYABC coalescent analyses. This diversity is driven by polymorphism in eastern Pacific populations, even though eastern Pacific populations are derived from western Pacific populations [19]. But the eastern Pacific region is more thoroughly sampled than the western Pacific region. The low nucleotide diversity in the Bay of Biscay contrasts with the high haplotype diversity. All other groups have higher nucleotide diversity and lower haplotype diversity than the Bay of Biscay. This suggests that the Bay of Biscay was colonized by many genetically similar haplotypes. The northwestern Atlantic region's high θ values, as well as the median joining network and the DPA, support the previously published conclusion that this region contains multiple distinct haplotypes [53].

The population structure of Clade A suggests that gene flow is somewhat restricted between geographic regions, as 30% of the molecular variation at mtCOI was found among regions (as opposed to only 12% for Clade E). This conclusion confirms previous analyses, which found evidence of restricted gene flow with isolation-by-distance for haplotypes HB and HJ, and restricted gene flow with some long-distance dispersal for haplotypes HA, HI, and HP [46]. These results are strikingly similar to two other closely related ascidians that are also globally distributed: *Botrylloides nigrum* and *Botrylloides violaceus*. *B*. *nigrum* has very similar levels of mtCOI population structure to Clade A when populations are pooled by ocean (Atlantic, Mediterranean and Pacific): 29% of the variation is found among regions [85]. However, when populations are pooled into basins (northwestern and southwestern Atlantic, Gulf of Mexico, North and South Caribbean, Central, Eastern and Indo-Pacific), only 11% of the variation is found among regions and this variation is not statistically significant. This difference in population structure in ocean vs. basin is not evident in Clade A *B*. *schlosseri*. *B*. *violaceus* also has a similar mtCOI population structure to Clade A when populations are pooled by ocean: 28% of the variation is found among regions [19].

Several other widespread ascidians have substantially less genetic structure at mtCOI than the botryllids. The *F*_CT_ value of Clade A is 0.39. The solitary ascidian *Styela clava*, introduced throughout the temperate Atlantic and Pacific oceans, has a *F*_CT_ value of 0.08 (when partitioned by geographic regions) and 0.25 (when partitioned by groups assigned through Bayesian analysis) [18]. The solitary ascidian *Styela plicata* has a similar *F*_CT_ to *Styela clava*: 0.09, with only 9% of the variation between oceans [86]. *Diplosoma listerianum* Clade A, found in all major temperate and tropical bodies of water, exhibits no mtCOI variation among geographic regions (Atlantic, Mediterranean, Indian and Pacific) [27].

The difference between the two groups of ascidians (botryllids vs. styelids/*D*. *listerianum*) in levels of population structure among oceans likely reflects the amount of time these two groups of species have been spreading outside their native ranges: the botryllid expansion may be substantially older than the styelid and *D*. *listerianum* expansion. However, *S*. *plicata* may be an exception to this narrative: phylogenetic trees for mtCOI and nuclear ANT (Adenine Nucleotide Transporter) show a deep divergence within this species, despite very little geographic structuring [86]. In this instance, recent introductions seem to have masked an ancient global range expansion.

The Clade A Bayesian phylogenetic tree and the median joining network present many abundant haplotypes with geographically distinct origins. This pattern, in conjunction with the substantial mtCOI population structure between regions, confirms Clade A as a species that has been well-established throughout the world's temperate oceans for centuries. Despite the complexity and longevity of Clade A's history, several patterns of dispersal can be inferred. The northwestern Atlantic was colonized multiple times: by the HA and HO haplotype groups (which originated in Europe), and by the two Pacific-origin haplotype groups (Bs1 and Bs10). On the other hand, Bs2, a native northwestern Atlantic haplotype [53], was introduced into the North Sea (Fig 1, S2 Fig). The North Sea was probably invaded a second time by the HB haplotype group, which seems to have originated in the Mediterranean (Table 3). The Mediterranean region is also the probable origin of the HK haplotype group, even though haplotypes HB and HK are not closely related. The Mediterranean also contains many HA and HO haplotypes; we cannot yet assign origins to these haplotypes with confidence. Like the northwestern Atlantic, the English Channel and the Bay of Biscay were both colonized many times, by haplotypes HA, HB, HD and HO. Both the English Channel (e.g. Bs39 and Bs48) and the Bay of Biscay (e.g. HI, Bs40, and Bs46) contain native haplotypes, but these haplotypes do not seem to have spread to other regions. These native haplotypes are all closely connected to widespread and abundant haplotypes, and likely evolved from these dominant haplotypes after colonization (e.g. HI from HA in the Bay of Biscay).

The Pacific region contains several distinct haplotypes that have spread to the northwestern Atlantic (Fig 1). Both of the most likely dispersal scenarios from coalescent analyses confirm this pattern: the northwestern Atlantic populations derive from the Pacific populations. In the median joining network, the Bs1 haplotype (Pacific origin) connects to the HO haplotype, which is very common in the northwestern Atlantic. This connection is separated by 12–14 mutations, which could represent unsampled haplotypes. In DIYABC Scenario 21, the northwestern Atlantic lineage evolves from Pacific populations through an unsampled intermediary (S3 Table). Yund et al. [53] does not view a Pacific origin for the Bs2 haplotype as likely, because the Pacific haplotypes Bs8 and Bs10/Bs36 seem to derive from other regions. However, haplotype Bs1 is noted as an exception to this pattern [53].

Authors of a previous mtCOI study considered a Pacific origin for Clade A unlikely based on a nested haplotype network which placed Pacific haplotypes Bs8 and Bs10 at the edges of the network and the Mediterranean clades in the center of the network [19]. Our median joining network also shows Bs8 and Bs10 far from the center (Fig 1), but both the nested haplotype network ([19]; Fig 2a) and the median joining network (Fig 1) place Bs1, a Pacific haplotype, in the center of the network. In DIYABC Scenario 26, the Mediterranean individuals derive from Pacific populations (S3 Table). Both the nested haplotype network and the median joining network support this scenario, as the Bs1 haplotype connects to haplotype HB (likely Mediterranean origin).

There is a deep split in the Clade A phylogeny (Fig 1 and S2 Fig) with HA/HD/Bs10 representing one sub-clade, and HB/HO/Bs1/Bs2 another. The node where this split occurs represents the most recent common ancestor of Clade A. Since the coalescent analyses clearly point to a Pacific origin for Clade A, the Pacific region would be predicted to be the most likely origin for this ancestral node. But the DPA does not identify this node, or the nodes representing the ancestors of each sub-clade, as originating in the Pacific.

Although the DPA did not support the Pacific origin of Clade A that was determined by coalescent analyses, Scenario 26, one of the two most likely dispersal scenarios from DIYABC, matches the DPA results. The ancestral node of the HA/HD/Bs10 sub-clade has a much higher likelihood of Mediterranean than northwestern Atlantic origin, whether the populations are split into four or six regions. This initial evolution of the HA/HD/Bs10 sub-clade likely occurred early in Clade A's history, soon after the Mediterranean lineage diverged from the ancestral Pacific lineage (S3 Table). The DPA assigns the ancestral node of the HB/HO/Bs1/Bs2 sub-clade similar probabilities for Mediterranean and northwestern Atlantic origins. In Scenario 26, sub-clade HB/HO/Bs1/Bs2 could have originated close to the time when the northwestern Atlantic lineage was initially evolving from the Mediterranean population, or after the northwestern Atlantic lineage had diverged in allopatry.

In summary, we have found support for a Pacific origin of Clade A from nucleotide diversity measures, a median joining network, and ABC scenarios, but not from the DPA. Since the eastern Pacific populations derive from the western Pacific, the origin is likely in the Indo-Pacific, as originally proposed by [55]. An increased sampling of western Pacific *B*. *schlosseri* would be needed to confirm this origin. A western Pacific origin of Clade A was supported by [46] to explain low mtCOI haplotype diversity and absence of intermediate haplotypes in Europe.

### Geographic history of Clade E

Average polymorphism levels are highest in the Mediterranean, but this is not evidence for a Clade E origin in the Mediterranean. This result is driven by the diversity in Sete, France; this population contains both haplotypes Bs11 and Bs12. Bs11 is a Mediterranean-specific haplotype, and Bs12 is more common in the English Channel than in the Mediterranean. This result shows that Sete, France has been colonized more than once by Clade E. The variable polymorphism levels among English Channel populations likely reflect the unique histories of anthropogenic transport to each location. This variation prevents us from using polymorphism data to make conclusions about Clade E's history in the English Channel as a whole. The low polymorphism levels in the Bay of Biscay populations suggest that a few Clade E haplotypes have recently invaded this area, as corroborated by the median joining network and DPA.

Evidence of population structure is observed in Clade E; statistically significant differentiation was found among populations within groups (*F*_SC_), among populations among groups (*F*_ST_), and among groups (*F*_*ct*_). Additionally, most of the 27 populations have statistically significant pairwise *F*_ST_ comparisons with at least 1/3 of the other populations. Patterns of differentiation between groups could reflect either divergence when Clade E initially expanded from its ancestral region, or current human-mediated gene flow, or both. DIYABC results strongly suggest that Clade E originated on the French side of the English Channel. The two English Channel regions are the most similar genetically, which could reflect a short coalescence time between the origin of the Clade E population on the French side of the English Channel and the subsequent divergence of the English populations. This similarity could also reflect ongoing gene flow between populations on either side of the English Channel, which is the busiest international seaway in the world. The Mediterranean and Bay of Biscay regions have very similar *F*_CT_ values when compared to populations on the English side of the English Channel, which makes sense if they diverged from the English populations at the same time (S3 Table, Clade E Scenario 19) and experienced similar evolutionary rates. The high genetic differentiation between the Mediterranean and Bay of Biscay could reflect a long period of allopatry between the two regions.

The median joining network of Clade E haplotypes looks similar to the statistical parsimony network in Bock et al. (2012), despite the different methodologies employed to generate the networks. Several conclusions can be inferred from the relationships displayed in these networks. First, there is a single ancestral haplotype; it is likely to be HH. Both the HH and Bs12 haplotype groups originated on the French coast of the English Channel (Table 7 and S10 Table). The HH haplotype is more geographically diverse and connects to more haplotypes than the Bs12 haplotype, making it a better candidate for the ancestral haplotype.

Second, the full genetic diversity of Clade E has not been sampled. Unsampled haplotypes occupy a central position in the haplotype network. The most probable DIYABC scenario also involves an unsampled lineage, which descended from the ancestral southern English Channel lineage and eventually gave rise to the northern English Channel and then Mediterranean and Bay of Biscay lineages (S3 Table: Clade E Scenario 19).

Third, given the distance between the endemic Mediterranean haplotypes and any other Clade E haplotypes in the median joining network, it is probable that unsampled lineages colonized the Mediterranean and gave rise to the HC, HE, HF, HW, Bs11 and Bs21 haplotypes. However, several scenarios were tested in DIYABC wherein Mediterranean populations were descended from unsampled lineages; none of these scenarios were considered most probable in comparison to other scenarios in the same ancestral set (S3 Table: Clade E Scenarios 14, 16, 18, 20, 21). However, the most likely DIYABC scenario, whereby the Mediterranean region was colonized by English individuals, is not incompatible with the median joining network.

Fourth, gene flow in both directions across the English Channel has occurred in Clade E. If Clade E originated in France, the presence of two widespread yet genetically distinct haplotypes (Bs18 and Bs34) in England suggests that this area has been colonized at least twice. Since the HH and Bs12 haplotypes are French in origin, and there are many English individuals with HH and Bs12 haplotypes, HH and Bs12 alleles presumably moved north across the Channel. The Bs18 haplotype derives from the widespread HH haplotype, and could have evolved from the HH haplotype when it moved across the Channel to England. The 25% of Bs18 haplotype individuals from France could represent a later introduction of France of the English Bs18 haplotype. The individuals with Bs34 haplotypes (and related satellites) are all located in the northern English Channel, except for a few individuals in the southern English Channel. As with the French Bs18 haplotype individuals, the Bs34 haplotype individuals in France could be the descendants of an introduction into France from a Bs34 haplotype that evolved in England. Lastly, the Clade E populations in the Bay of Biscay are non-native, and all haplotypes derive from the geographically diverse (and presumed ancestral) HH haplotype. According to the most probable demographic scenario, Bay of Biscay populations were colonized by haplotypes from the HH haplotype group from the English Channel coast of England.

In the Bayesian phylogenetic tree of Clade E produced by BEAST as part of the DPA, the Mediterranean haplotypes HE and Bs11 form a sister group to the rest of the phylogeny (S3 Fig). This relationship is well supported, with a posterior probability of 1.0. Although this tree does not have an outgroup, this result is reproduced in a rooted Bayesian tree [53], a rooted Maximum Likelihood Tree [46], and a rooted Neighbor-Joining Tree [26]. This does not imply that these Mediterranean haplotypes are ancestral, only that the HE/Bs11 lineage and the rest of the Clade E lineages diverged from a common ancestor at the same time [87]. This contradicts the coalescent analysis implemented in DIYABC, which indicates that Mediterranean lineages emerged later than English Channel lineages. However, the DIYABC scenarios are necessarily simplified. Because all the Mediterranean populations were grouped together, we did not test a scenario that separated the evolutionary history of HE/Bs11 from the other Mediterranean endemic haplotypes. It is indeed probable that the HE/Bs11 lineage diverged very early from the ancestral Clade E population, given the substantial genetic separation between HE/Bs11 and the rest of Clade E (Fig 5 and S3 Fig).

The two major clades in the Clade E phylogeny both have Mediterranean endemic haplotypes as sister taxa (haplotype HF is sister to an English Channel + Ireland clade corresponding to the Bs34 haplotype group in the median joining network andHC/HW/Bs21 is sister to a diverse clade corresponding to HH/Bs12/Bs18 group in the median joining network) (S3 Fig). However, these sister group relationships are not well supported (0.28 and 0.37 posterior probability values, respectively). The positions of HF and HC/HW/Bs21 are different in every published Clade E tree [26,46,53]. This uncertainty is also reflected in the median joining network, in which HF and HC/HW/Bs21 are connected to the rest of the network by nodes representing unsampled haplotypes (Fig 5).

The DPA strongly supports the hypothesis that the French side of the English Channel is the origin of the HH haplotype group (Table 7 and S10 Table). The HH haplotype, although geographically diverse, is most abundant in France (Fig 5). The DPA also ascribes a French origin to the Bs12 haplotype group. The Bs18 and Bs34 haplotypes are both clearly of English origin (Table 7 and S10 Table). The Bs18 haplotype is more abundant in England than any other region, and the Bs34 haplotype is only found in a few non-English individuals (Fig 5). When the HH and Bs18 haplotype groups are analyzed together, the English Channel coast of France is considered to be the origin (location posterior probability: 0.477), but less confidence can be placed in this assignment than in the origin of the HH or Bs18 haplotype groups when analyzed alone (0.606 for English Channel France and 0.835 for English Channel England, respectively). This lack of confidence arises when trying to determine the origin of a clade composed of a strongly French-origin clade and a strongly English-origin clade.

While we cannot rule out the possibility that the origin of Clade E lies in the unsampled haplotypes, a coalescent analysis of all available sequences supports the origin of Clade E on the French coast of the English Channel. The original French haplotype was likely HH (or an unsampled haplotype closely related to HH). The HH haplotype group is the most geographically widespread and genetically well-connected of the two haplotype groups (HH and Bs12) that originated in the southern English Channel. The HH haplotype group then colonized the northern English Channel, giving rise to the Bs18 haplotype group. The English Bs34 haplotype group likely evolved from an unsampled haplotype group related to HH, given the greater genetic distance between the HH and Bs34 groups than between the HH and Bs18 groups. The Bs18 and Bs34 haplotype group appear to be the result of separate colonizations of the English Channel. Individuals of the HH haplotype group colonized the Bay of Biscay, as all Bay of Biscay haplotypes are HH or 1–2 mutational steps away from haplotype HH. According to the DIYABC analysis, Bay of Biscay populations evolved from English individuals. If this is correct, the Bay of Biscay was colonized by English individuals with HH-like haplotypes. The dispersal history of Clade E into the Mediterranean is unclear, as the Mediterranean-endemic haplotypes are genetically divergent from non-Mediterranean haplotypes. The ancestors of two haplotypes (HE and Bs11) could have colonized the Mediterranean very early in the history of Clade E, but this possibility was not tested in DIYABC. The median joining network suggests that individuals whose haplotypes may not be represented in this data set were introduced to the Mediterranean region, but these scenarios were not supported in the coalescent analyses. Instead, the northern English Channel region was considered the source of Mediterranean Clade E populations.

### Geographic history of Clade A and Clade E

Clade A has been characterized as a successful invader, owing to its broad geographic range, genetically divergent haplotypes within single populations and widespread haplotypes within regions [26]. Each of these criteria can also be applied to Clade E. While Clade A has a wider geographical range than Clade E, Clade E is not restricted to the English Channel, as previously thought [26]. Clade E is widespread in European locations where *B*. *schlosseri* has been sampled, including a probable recent introduction into the southern Bay of Biscay. Clade E also has dissimilar haplotypes within a single population. For example, Brixham, Falmouth, Plymouth, Port Pendennis, and Torquay (England), Cobh (Ireland), and Roscoff and Sete (France) all contain the genetically dissimilar Bs18 and Bs34 haplotypes. In each of these populations, each haplotype is well-represented. Clade E also has widespread haplotypes within regions: the Bs18 haplotype is found in five of the eight populations in England, and the Bs34 haplotype in seven of the eight populations. In light of these data, both Clade A and Clade E should be considered widely dispersed (Clade A globally, Clade E within Europe).

Whether the geographic history and origins of Clade A and E can inform the origin of the entire *B*. *schlosseri* clade depends on the phylogenetic relationships between the five clades in the species complex. There are several different tree topologies of the *B*. *schlosseri* species complex in the literature [26,46,48,53]: none of them converge on the same topology and none has strong bootstrap support or high posterior probability values for all nodes.

The *Botryllus schlosseri* species complex has long been thought to be native to Europe [56], and recent molecular work has provided support for this hypothesis. Clades B, C, D and E are so far found only in Europe, and Clade E originated in the southern English Channel. There is evidence from polymorphism and coalescent analyses that Clade A originated in the Pacific, but it also has had a long evolutionary history in the Mediterranean and has colonized the English Channel and the Bay of Biscay several times. The *B*. *schlosseri* species complex either originated in the Pacific Ocean and diversified after Clade A arrived in European waters, or originated in Europe, with ancestors of Clade A dispersing to the Pacific Ocean. A well resolved phylogenetic tree of *B*. *schlosseri* will allow us to determine the likely geographic origin of this species group.

## Supporting Information

S1 Table Clade A and Clade E sequence lists. (XLSX)

S2 Table Clade A and Clade E DIYABC priors. (XLSX)

S3 Table Visualizations of Clade A and Clade E DIYABC scenarios. (PPTX)

S4 Table Clade A and Clade E Polymorphism Statistics. (XLSX)

S5 Table Clade A and Clade E All pairwise Fst values. (XLSX)

S6 Table Clade A and Clade E Fst values averaged across groups. (XLSX)

S7 Table Clade A and Clade E List of Haplotypes. (XLSX)

S8 Table Clade A and Clade E haplotypes of sequences from this study. (XLSX)

S9 Table Clade A and Clade E haplotype frequencies for each population sampled in this study. (XLSX)

S10 Table Clade A 4 and 6 locations and Clade E posterior probability (DPA) Graph. (XLSX)

S11 Table Clade A and Clade E DIYABC Best Scenarios for each ancestral set. (XLSX)

S1 Fig Clade A 4 locations median joining network for publication. (EPS)

S2 Fig Clade A 6 Locations Bayesian Phylogeny. (EPS)

S3 Fig Clade E Bayesian Phylogeny. (EPS)
