# Supplementary material for: Origin and Dispersal History of Two Colonial Ascidian Clades in the Botryllus schlosseri Species Complex
Source: PLoS One. 2017 Jan 20;12(1):e0169944. doi: 10.1371/journal.pone.0169944 (PMC5249052; doi:10.1371/journal.pone.0169944)
Supplement: S3 Table — (PPTX) [file pone.0169944.s003.pptx]

## Slide 1
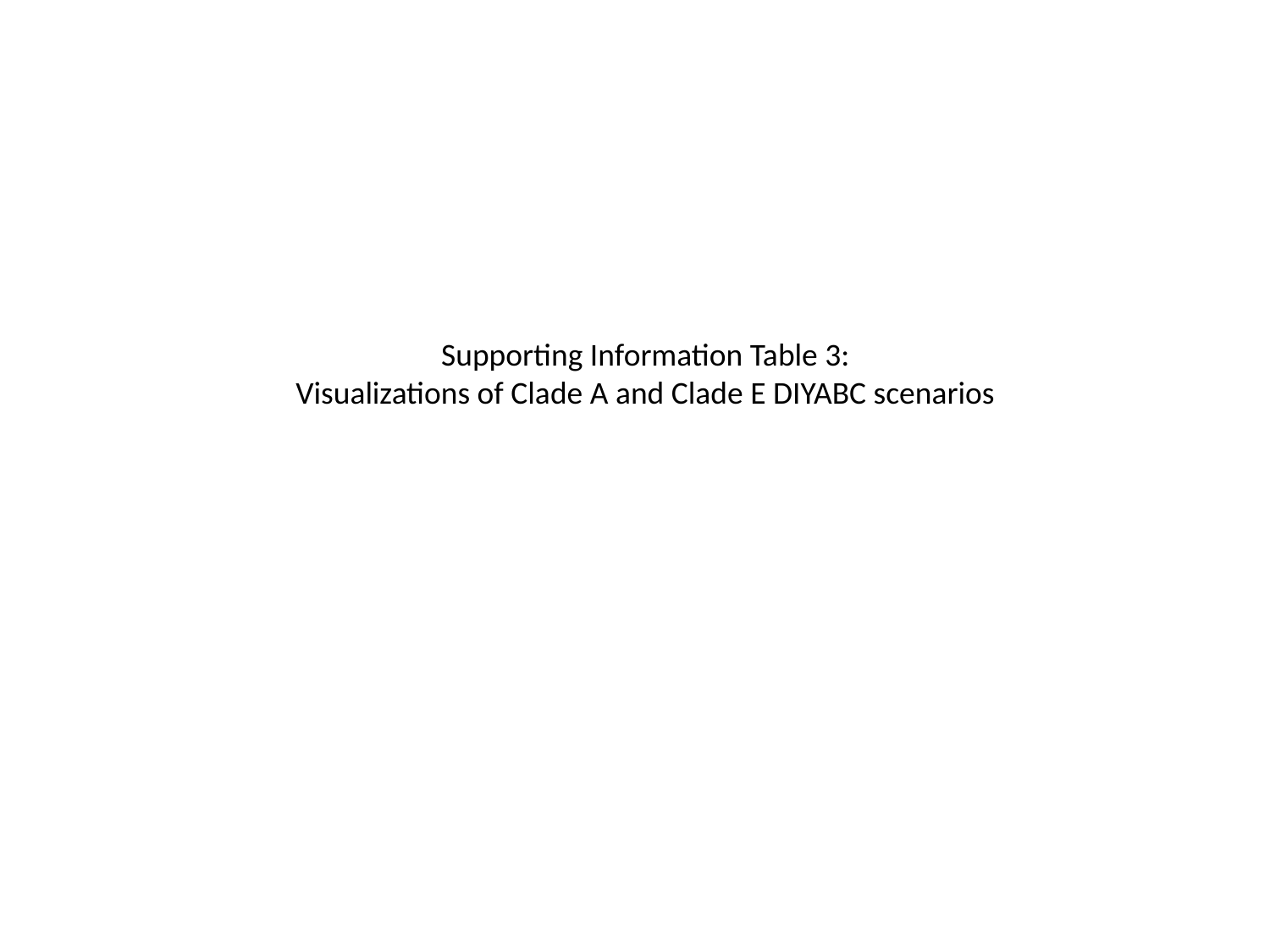

# Supporting Information Table 3: Visualizations of Clade A and Clade E DIYABC scenarios

## Slide 2
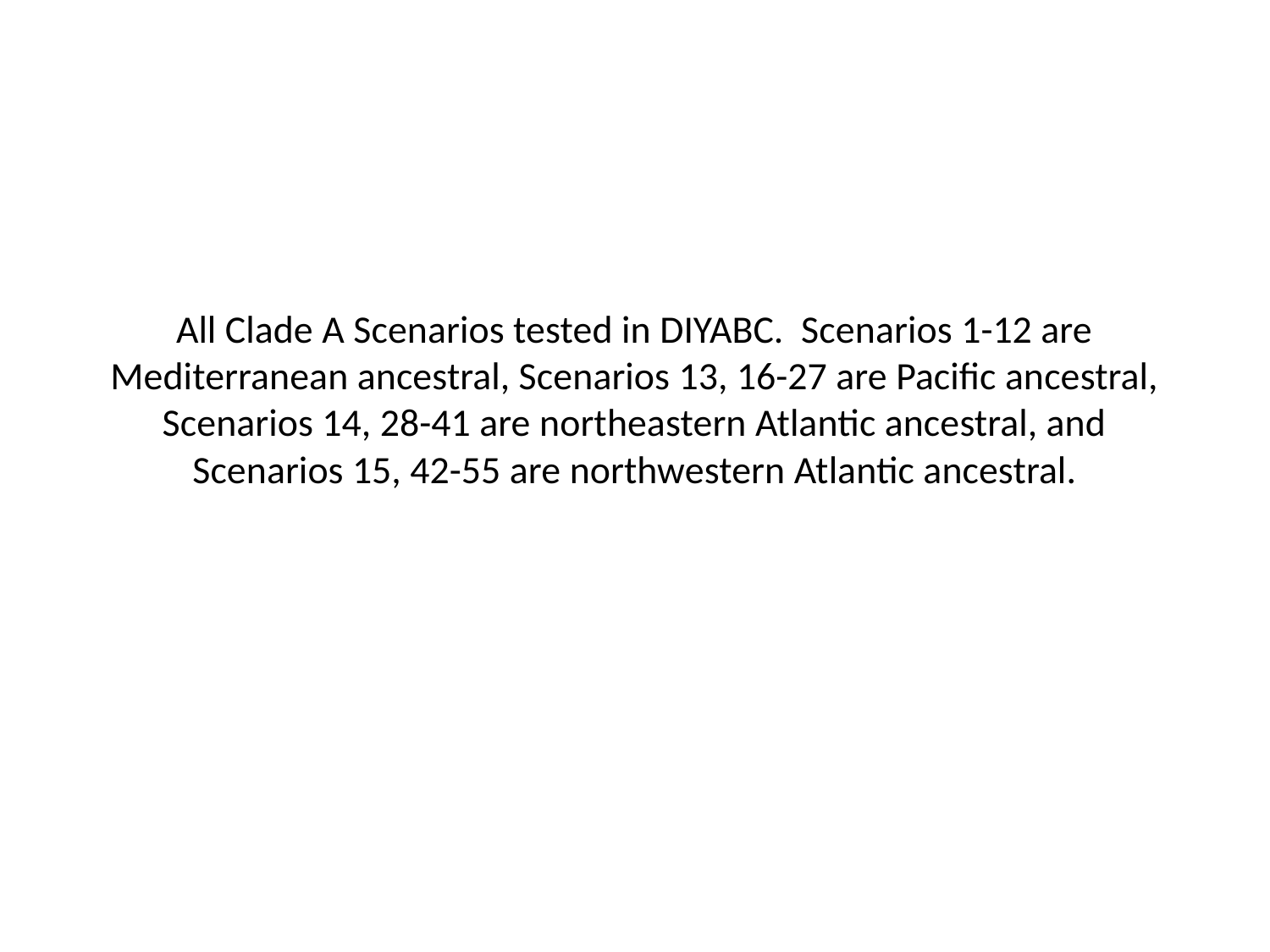

# All Clade A Scenarios tested in DIYABC. Scenarios 1-12 are Mediterranean ancestral, Scenarios 13, 16-27 are Pacific ancestral, Scenarios 14, 28-41 are northeastern Atlantic ancestral, and Scenarios 15, 42-55 are northwestern Atlantic ancestral.

## Slide 3
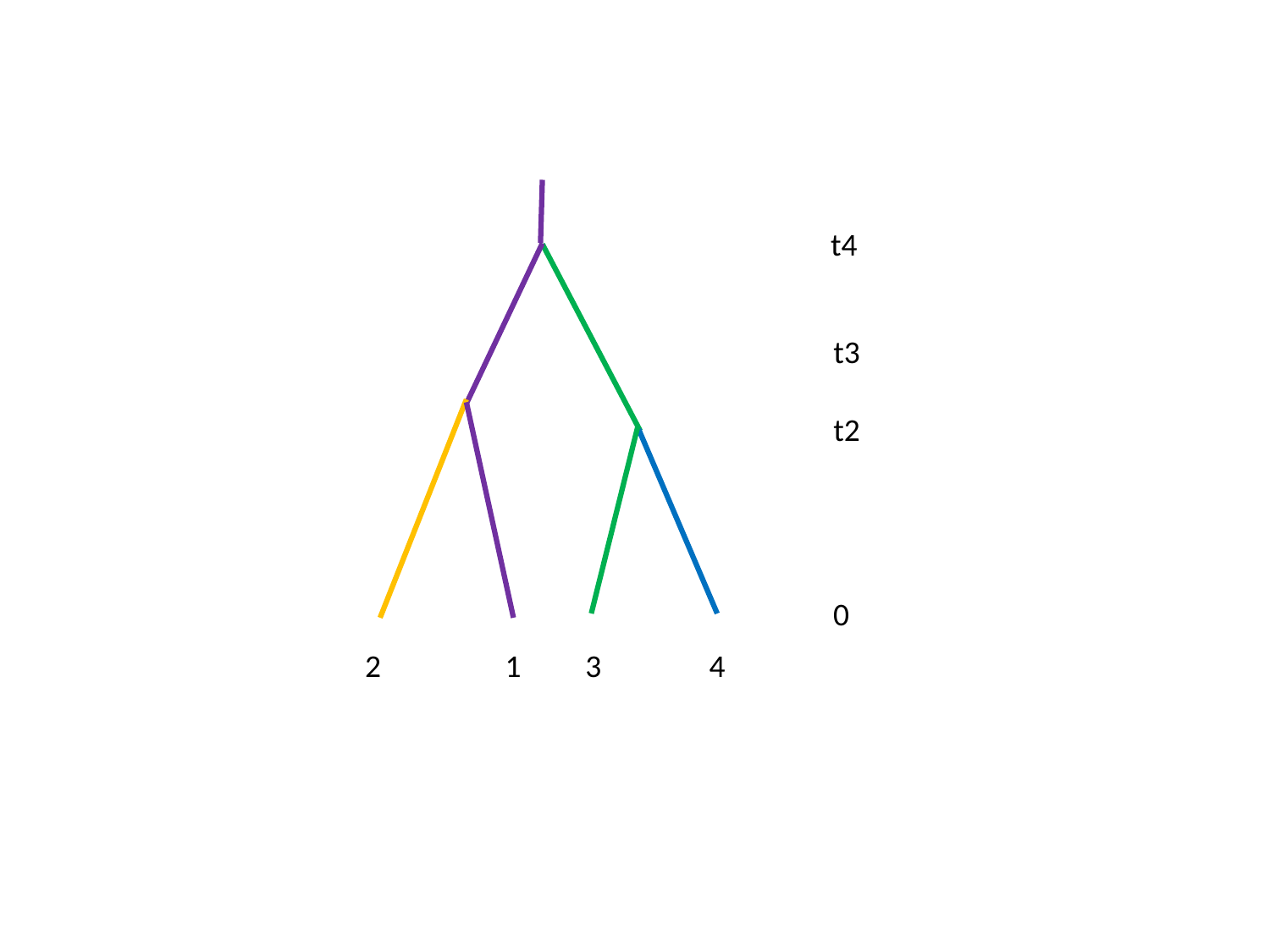

t4
t3
t2
0
2
1
3
4

## Slide 4
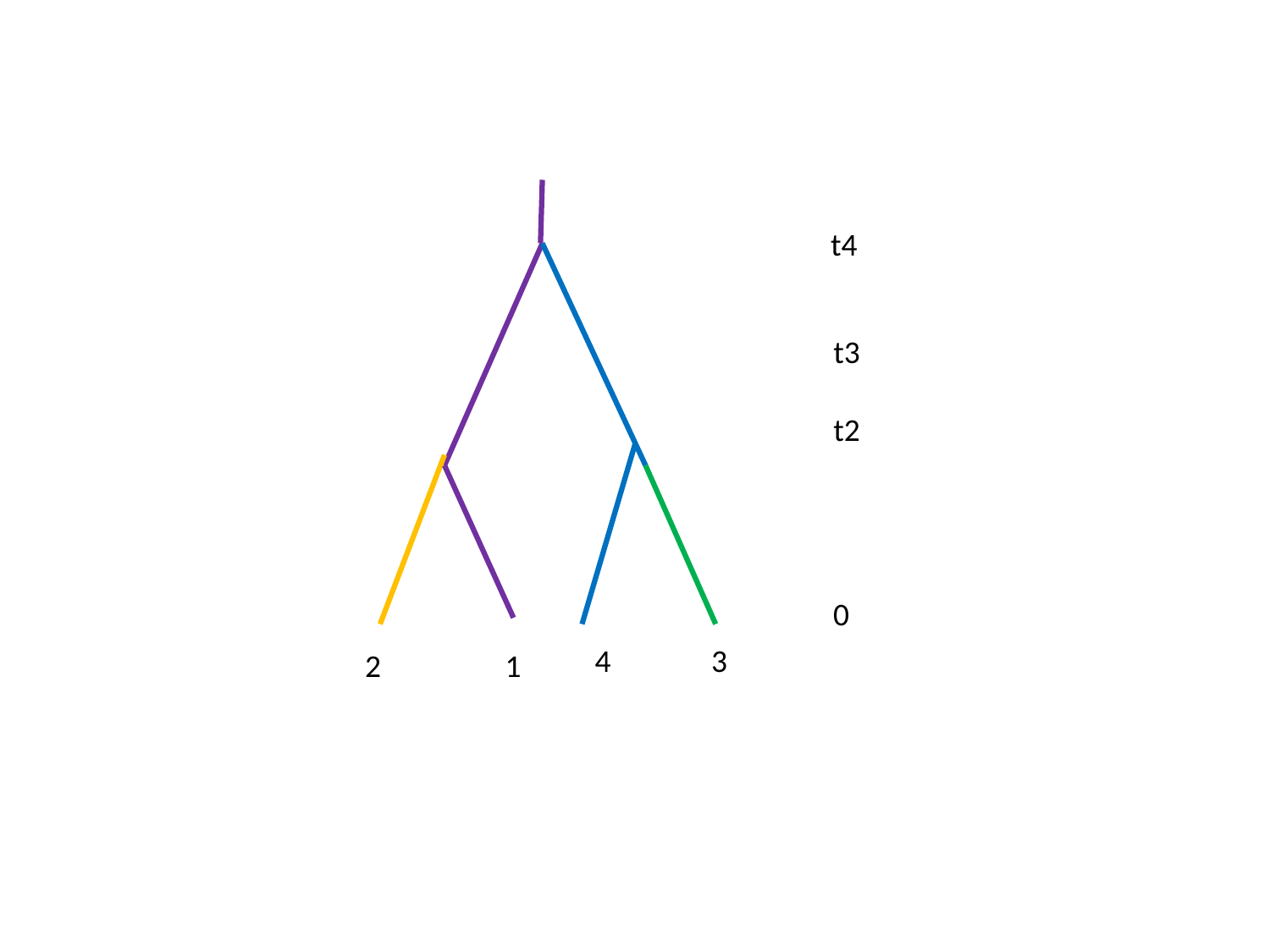

t4
t3
t2
0
4
3
2
1

## Slide 5
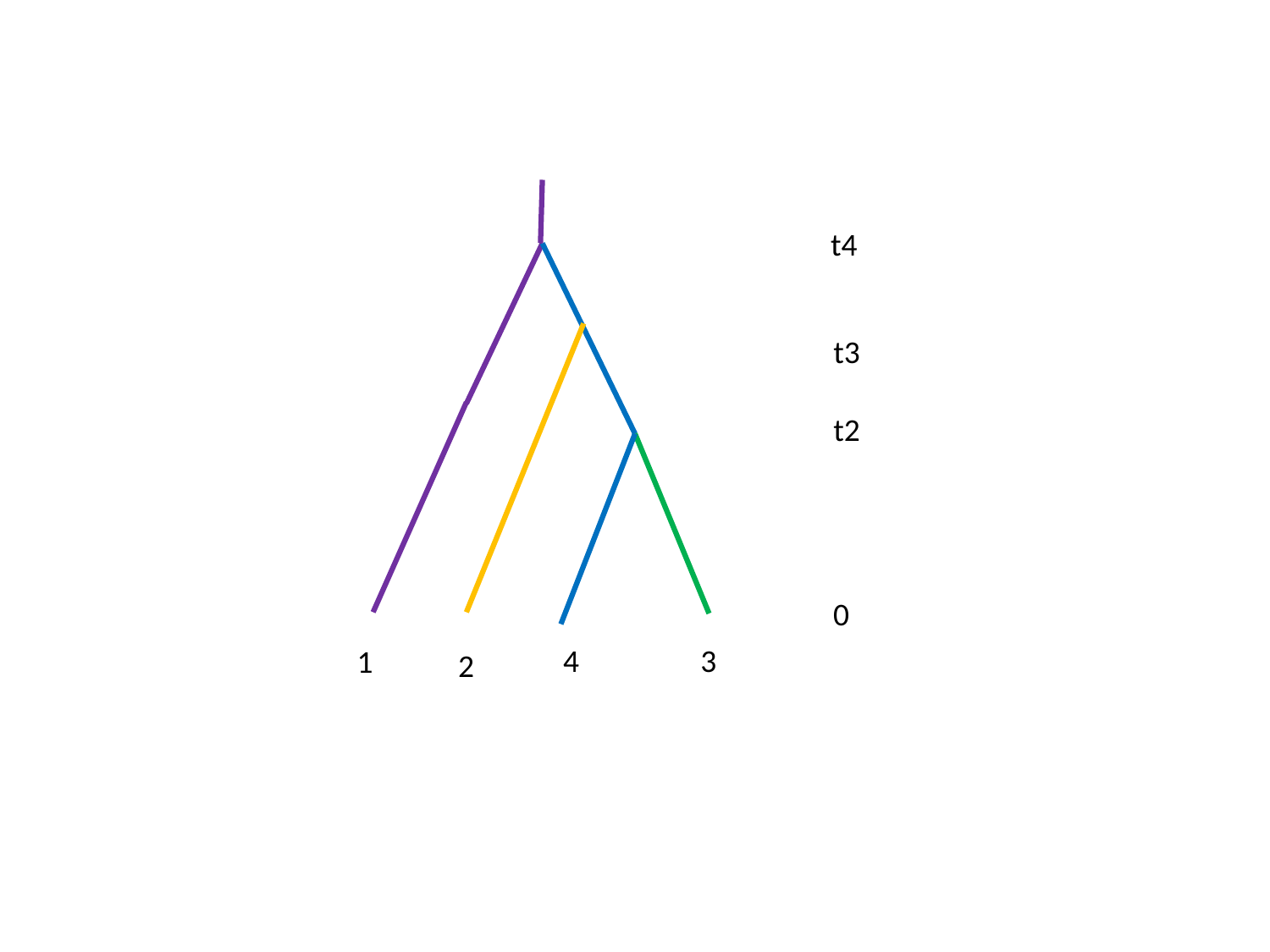

t4
t3
t2
0
4
3
1
2

## Slide 6
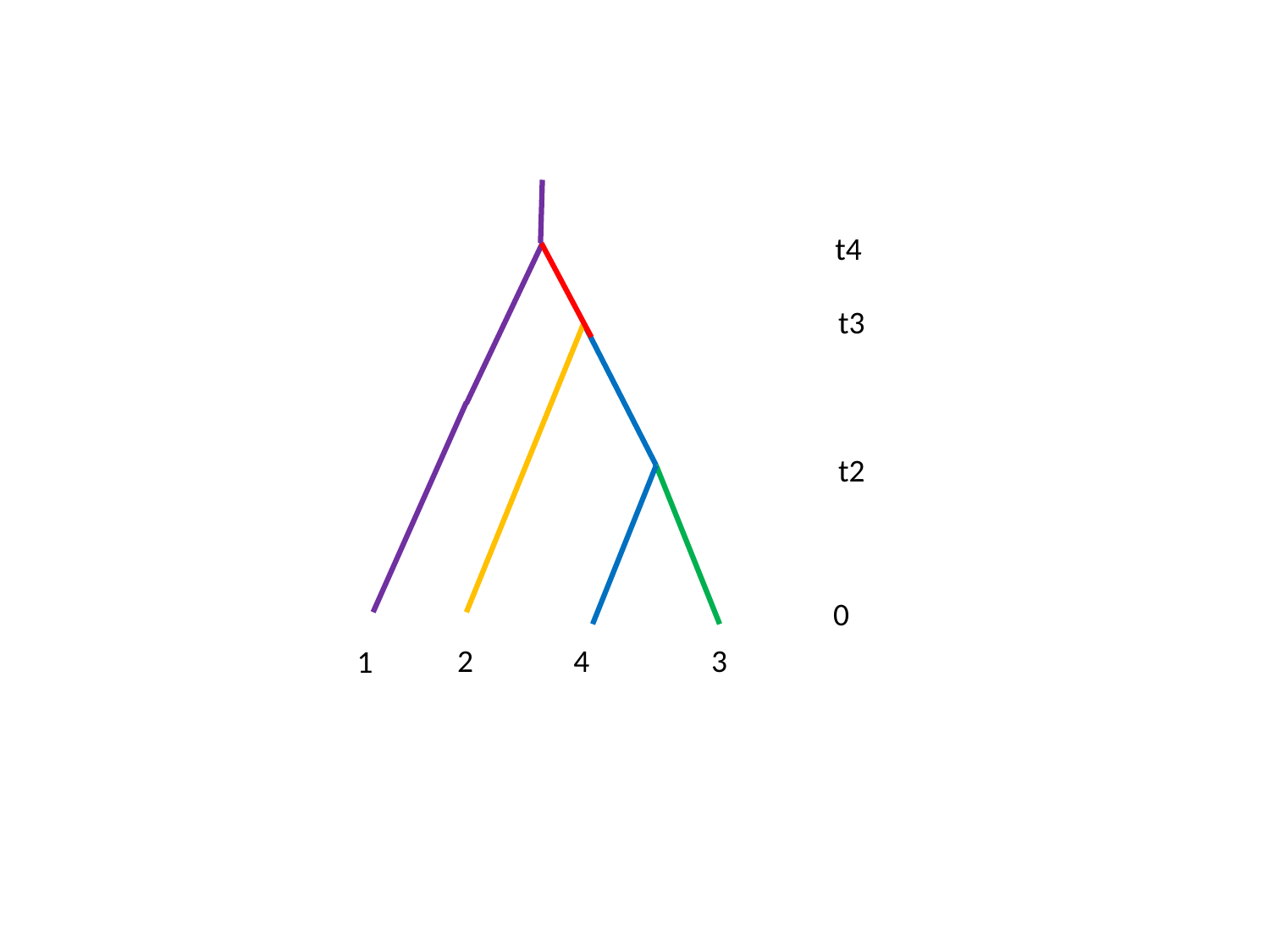

t4
t3
t2
0
2
4
3
1

## Slide 7
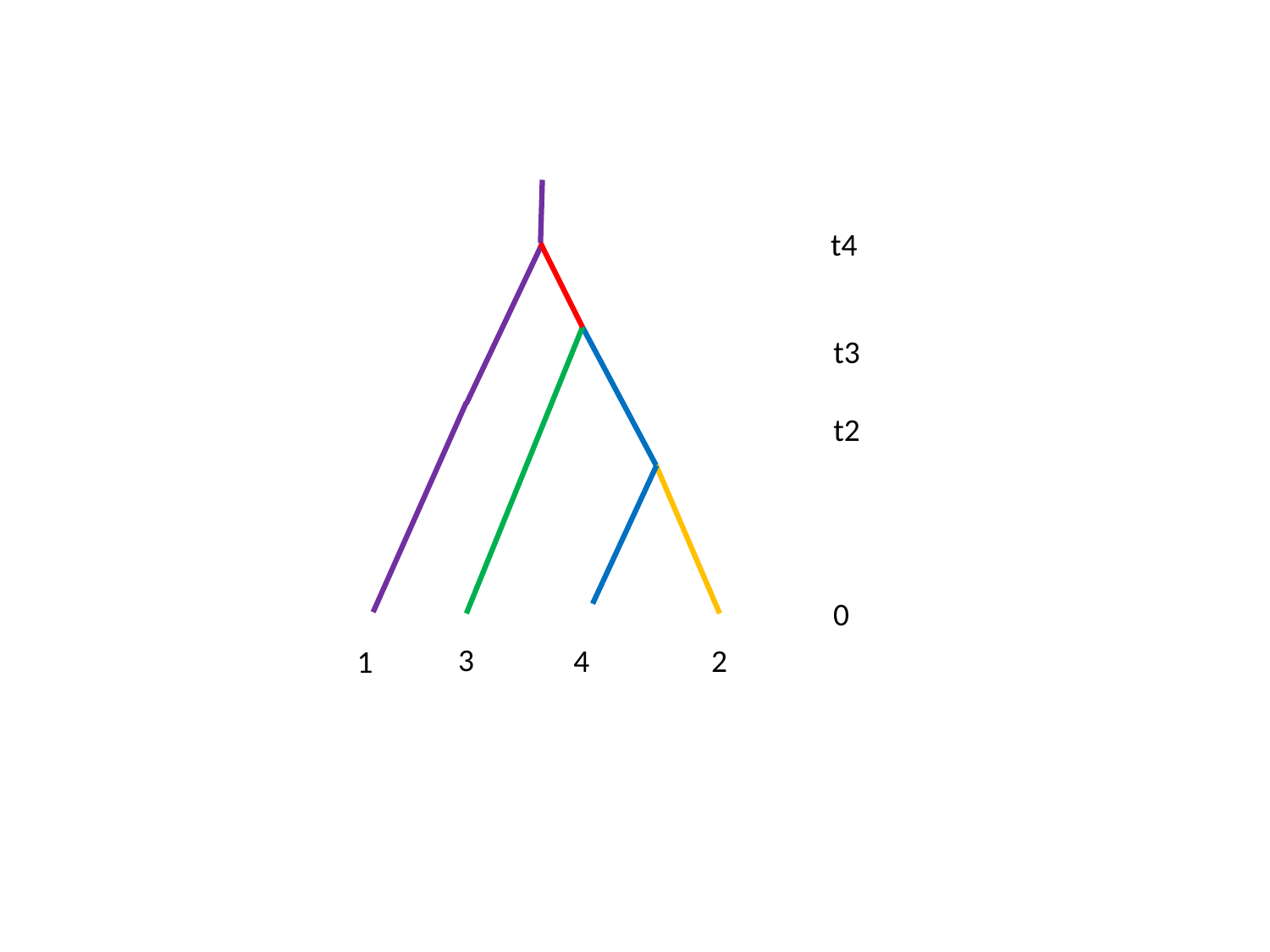

t4
t3
t2
0
3
4
2
1

## Slide 8
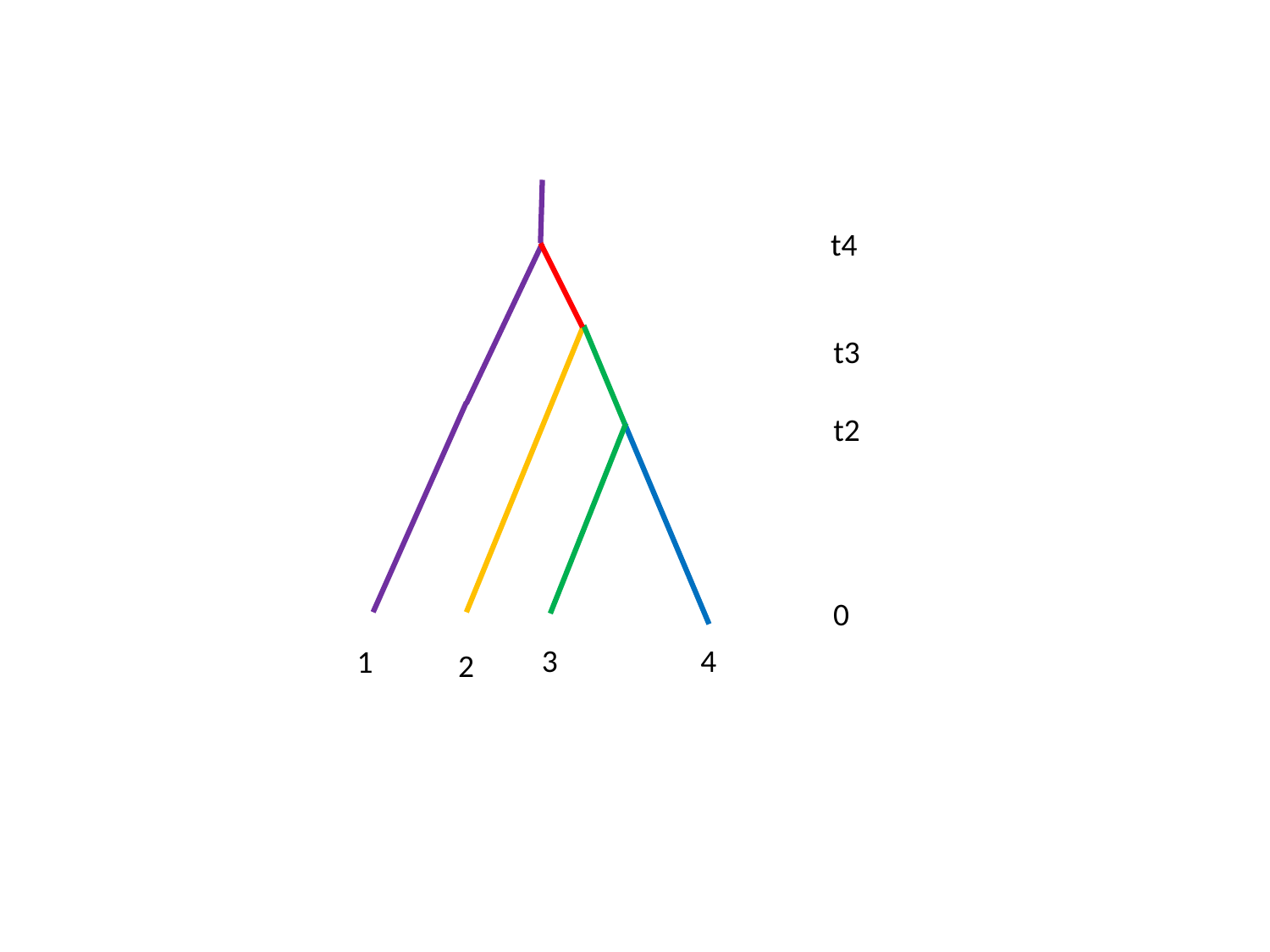

t4
t3
t2
0
3
4
1
2

## Slide 9
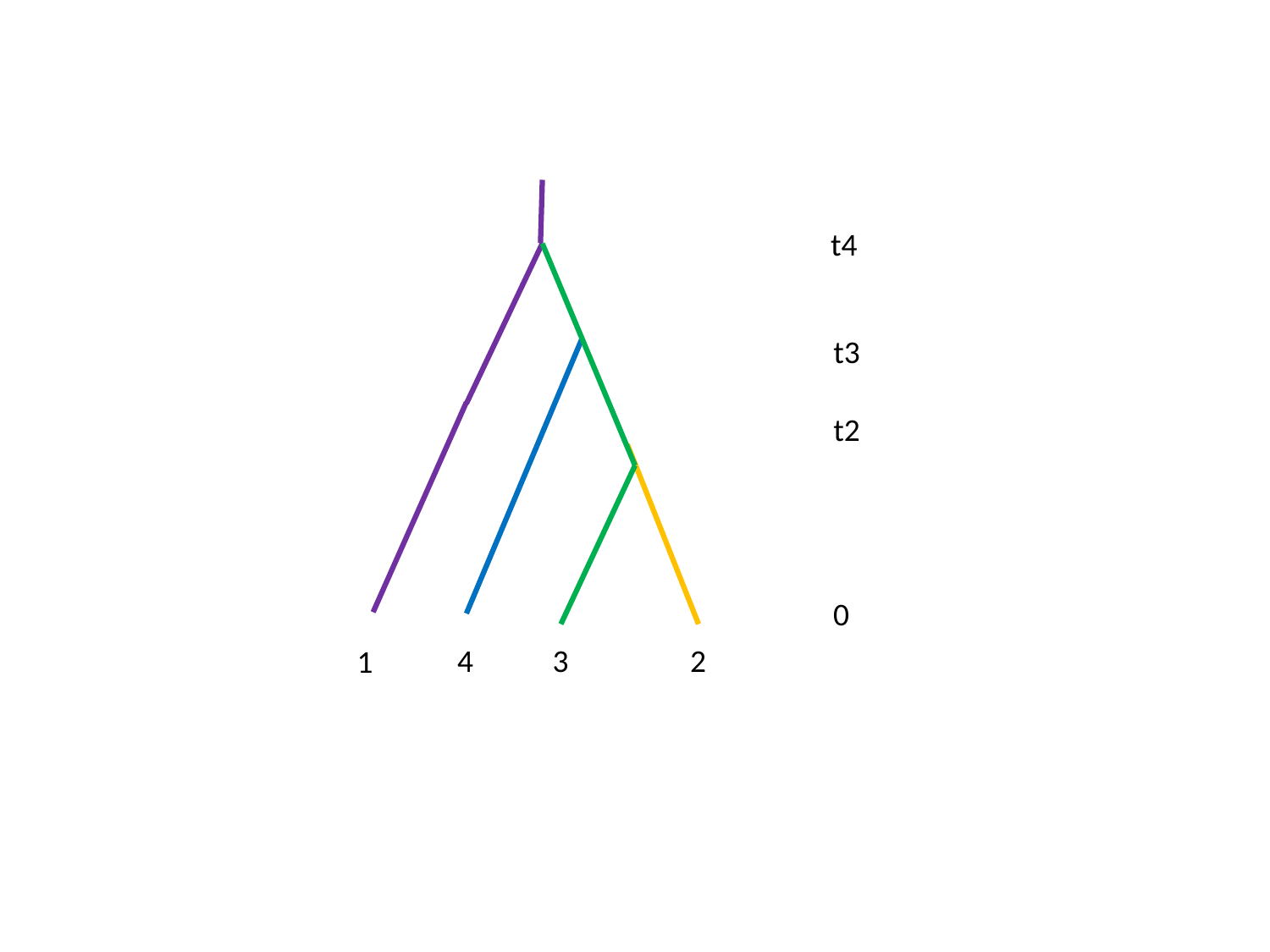

t4
t3
t2
0
4
3
2
1

## Slide 10
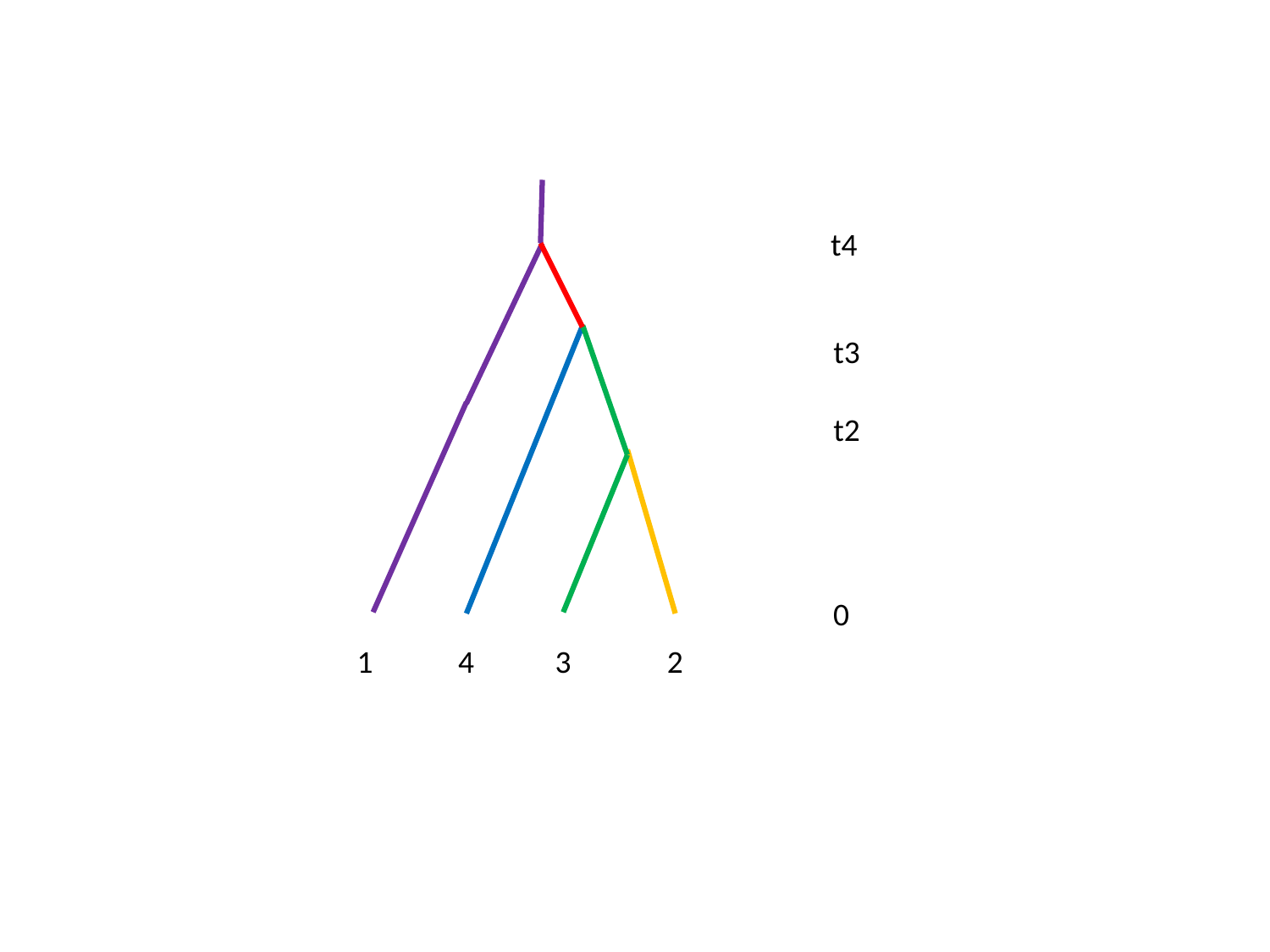

t4
t3
t2
0
1
4
3
2

## Slide 11
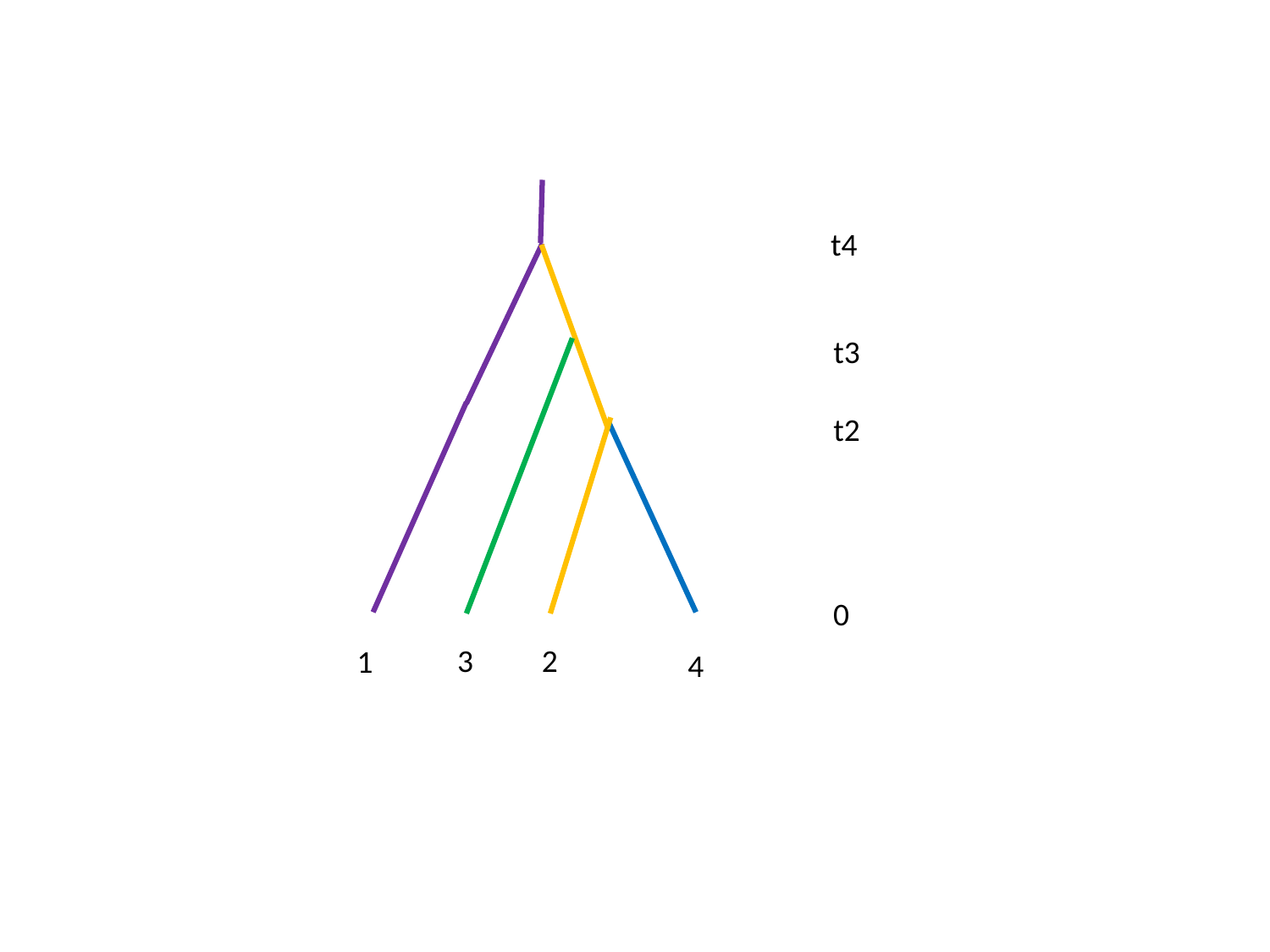

t4
t3
t2
0
3
2
1
4

## Slide 12
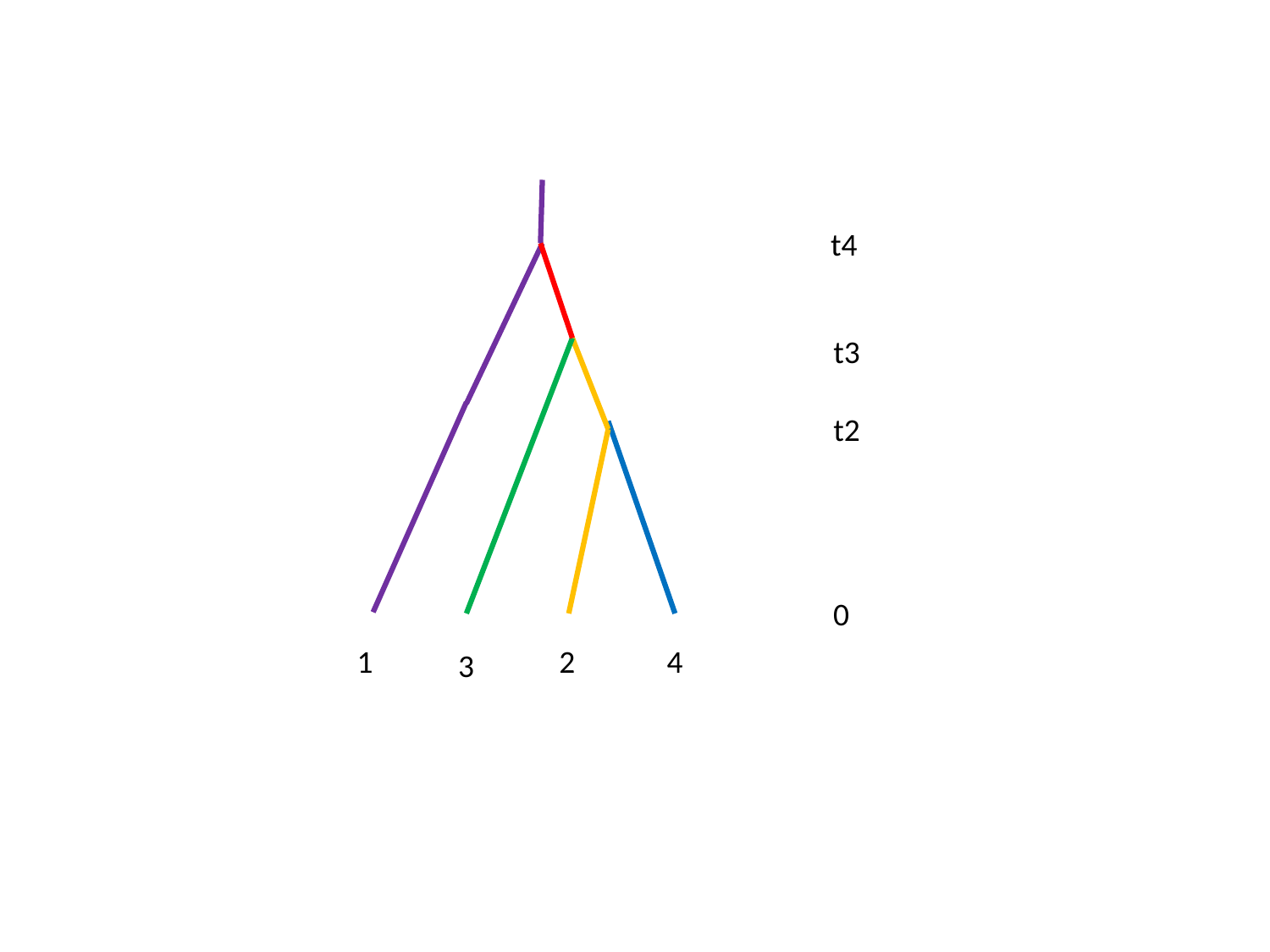

t4
t3
t2
0
1
2
4
3

## Slide 13
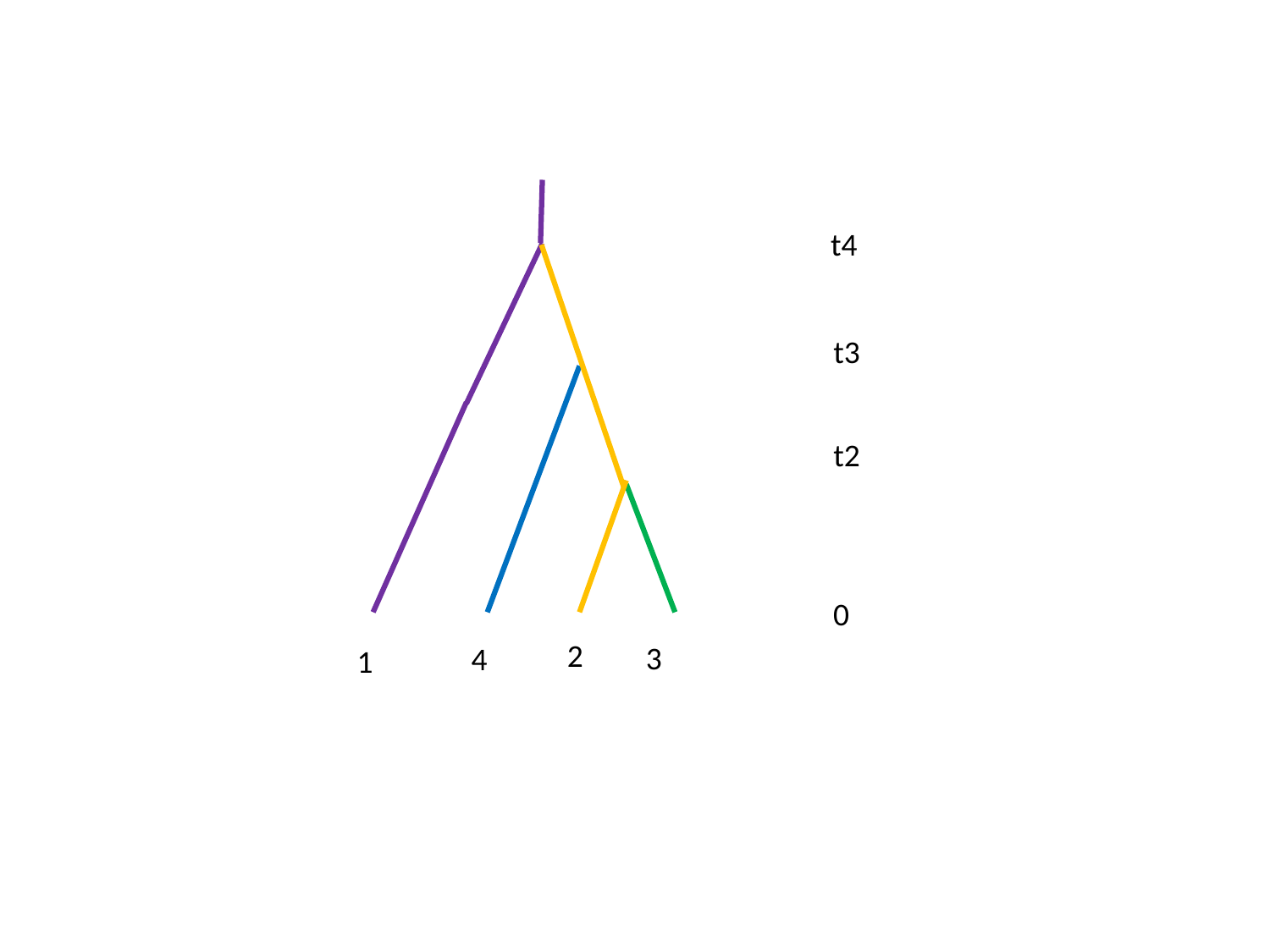

t4
t3
t2
0
2
3
4
1

## Slide 14
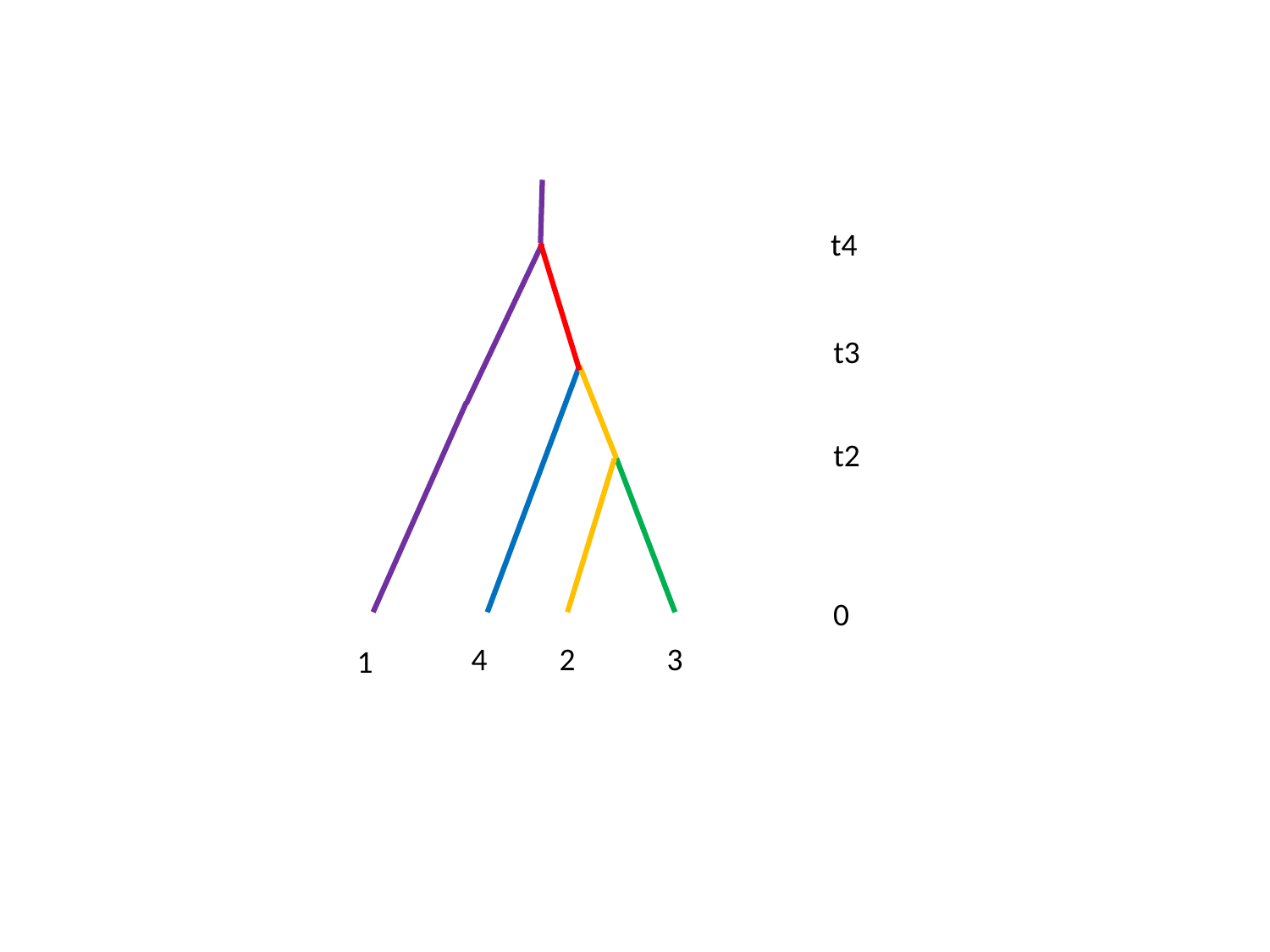

t4
t3
t2
0
4
2
3
1

## Slide 15
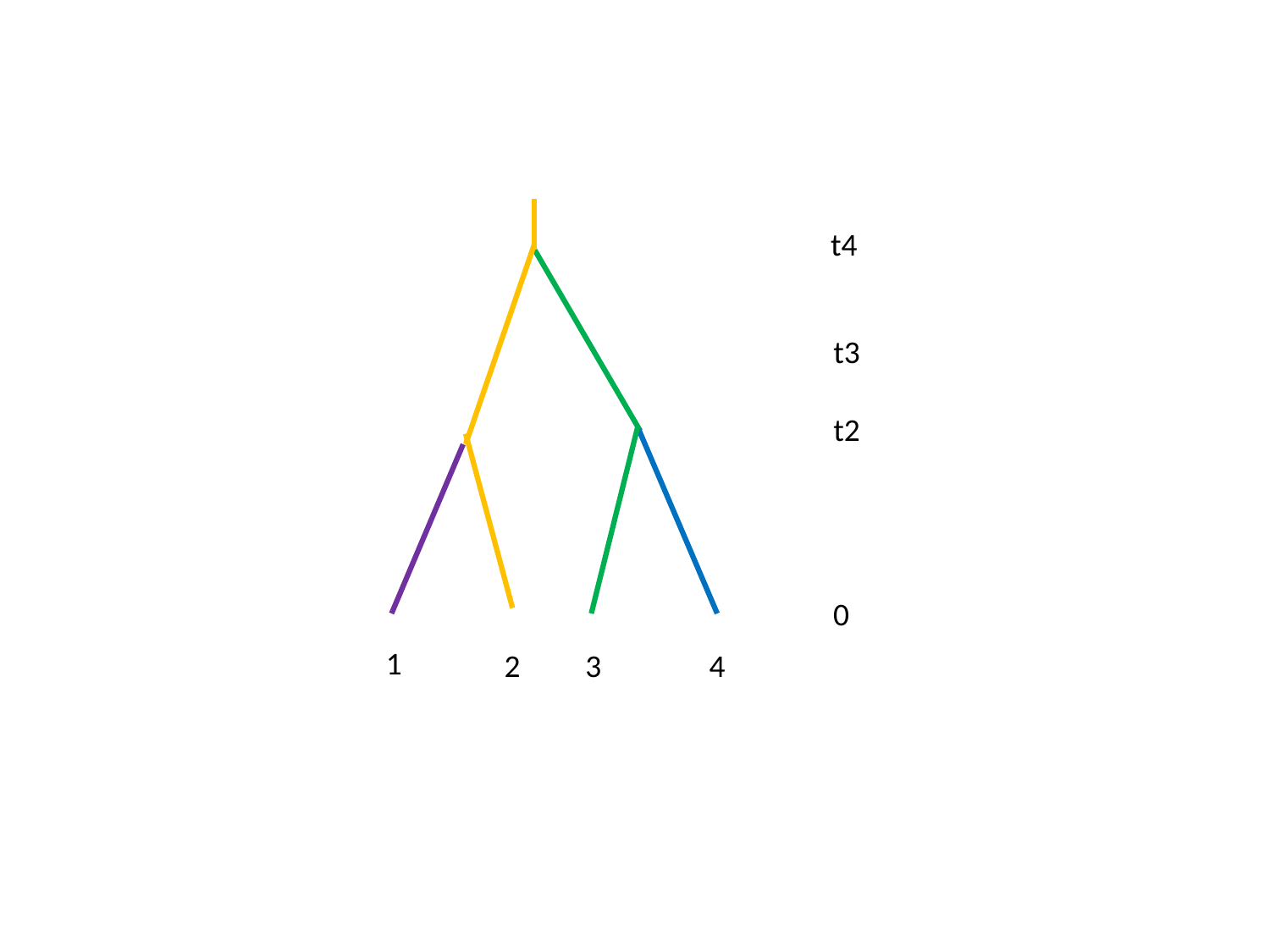

t4
t3
t2
0
1
2
3
4

## Slide 16
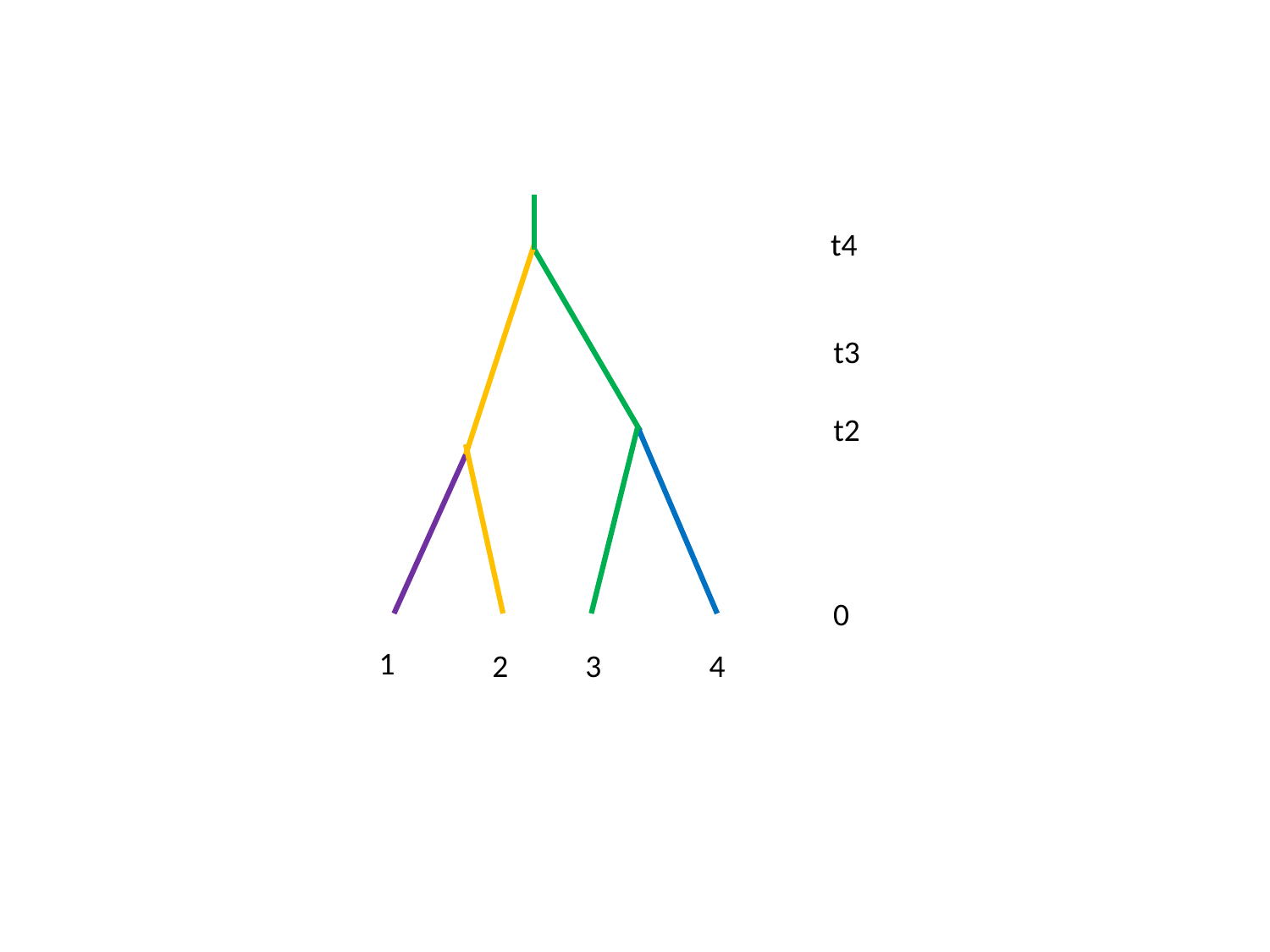

t4
t3
t2
0
1
2
3
4

## Slide 17
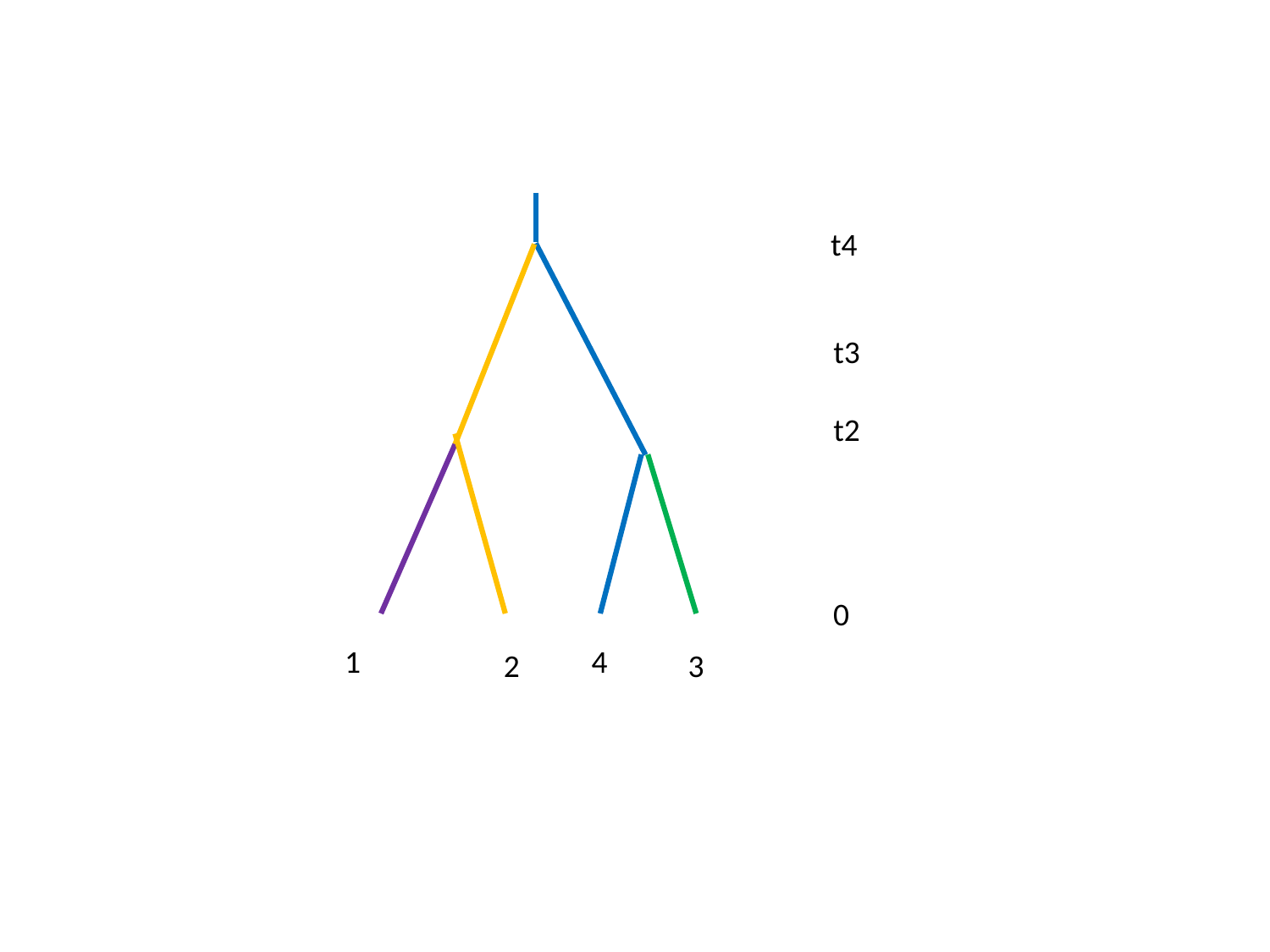

t4
t3
t2
0
1
4
2
3

## Slide 18
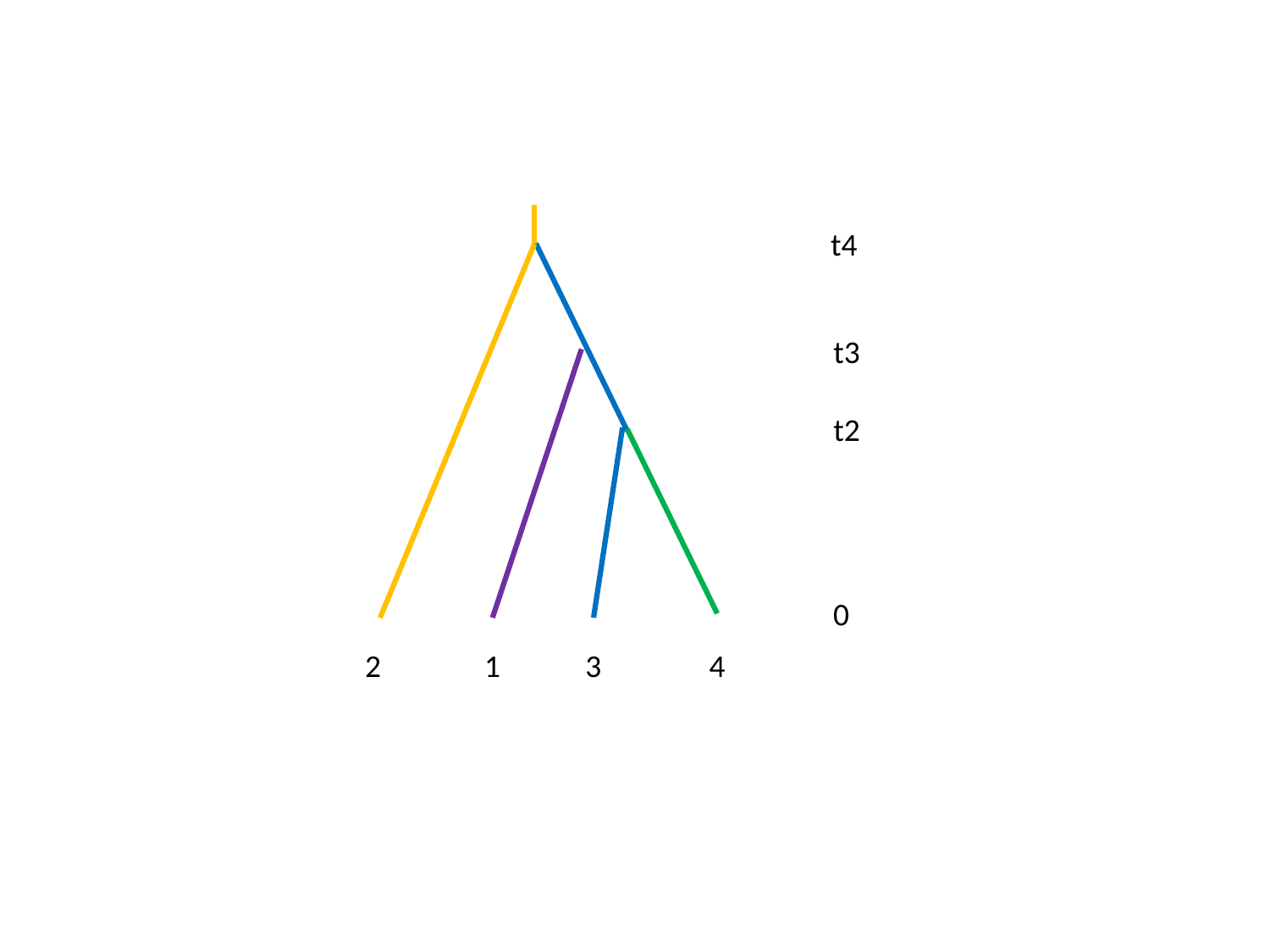

t4
t3
t2
0
2
1
3
4

## Slide 19
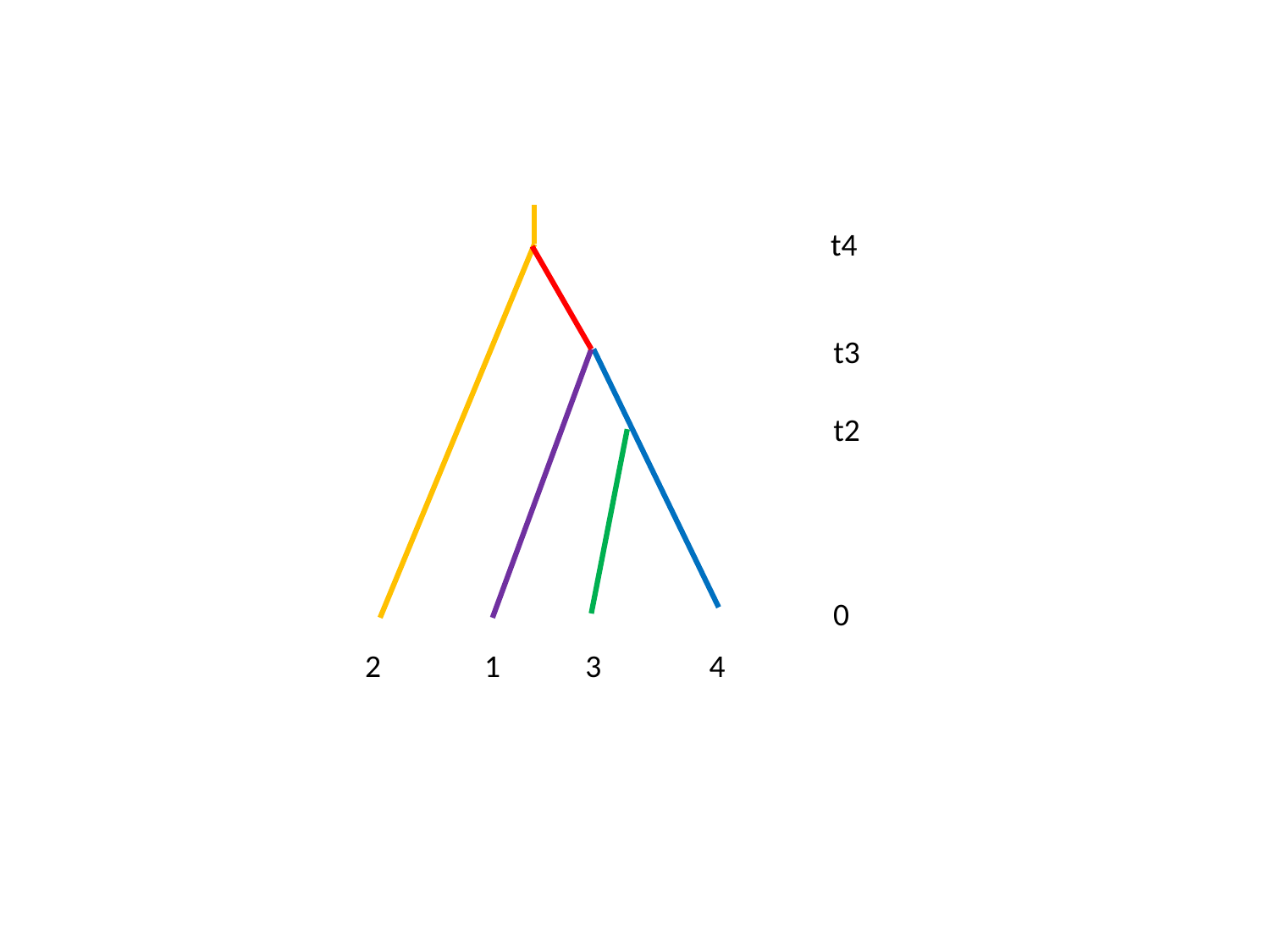

t4
t3
t2
0
2
1
3
4

## Slide 20
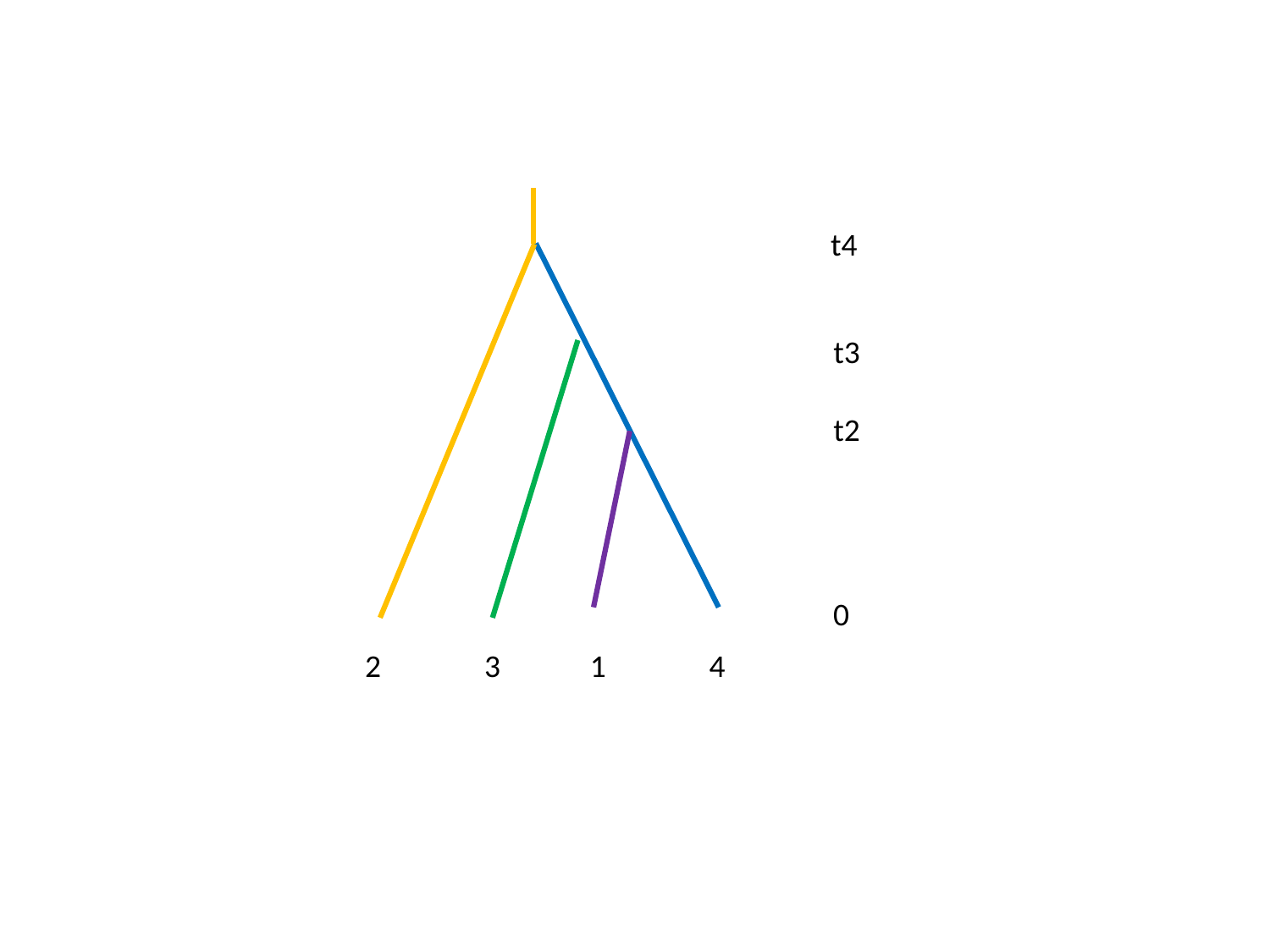

t4
t3
t2
0
2
3
1
4

## Slide 21
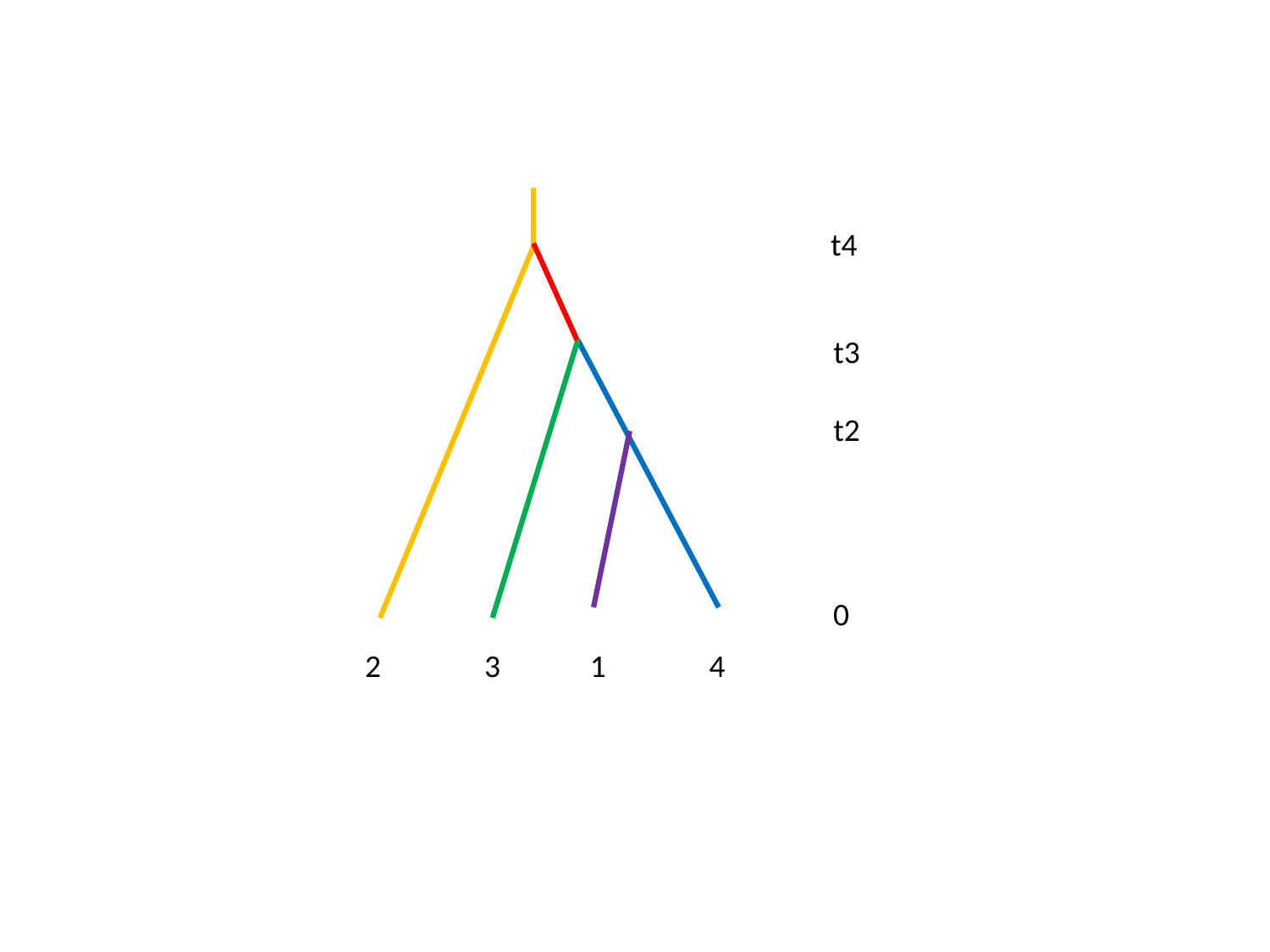

t4
t3
t2
0
2
3
1
4

## Slide 22
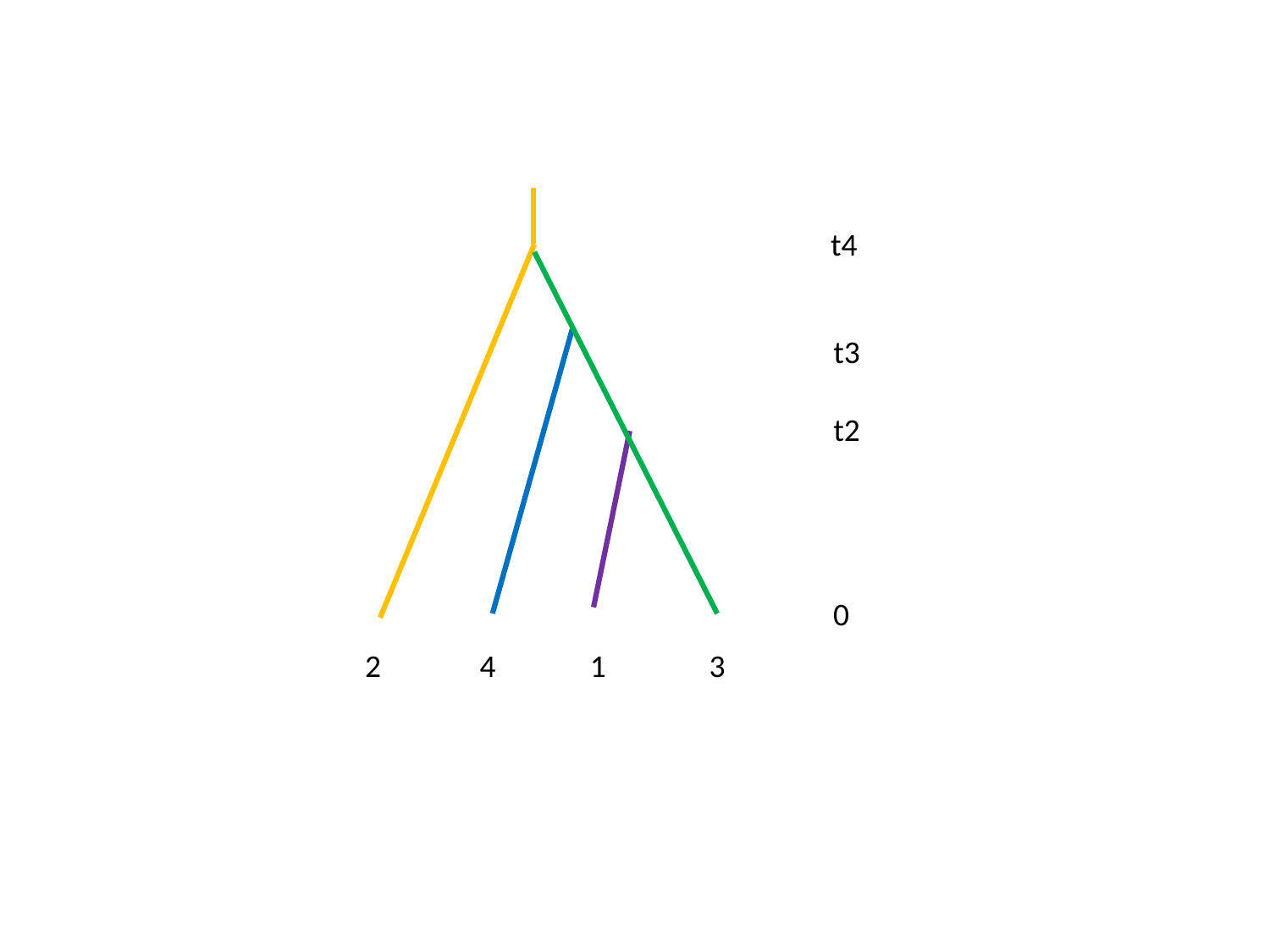

t4
t3
t2
0
2
4
1
3

## Slide 23
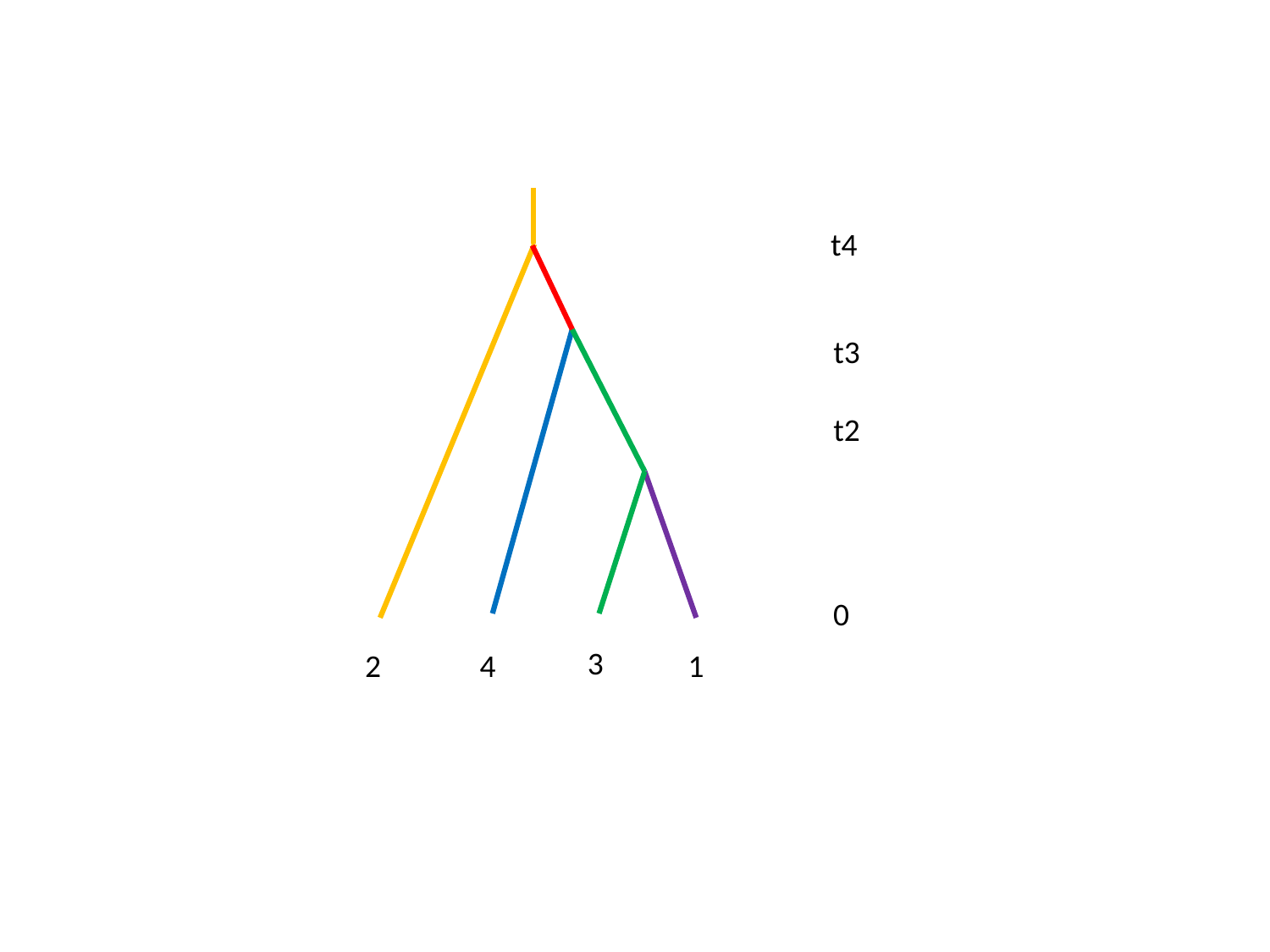

t4
t3
t2
0
3
2
4
1

## Slide 24
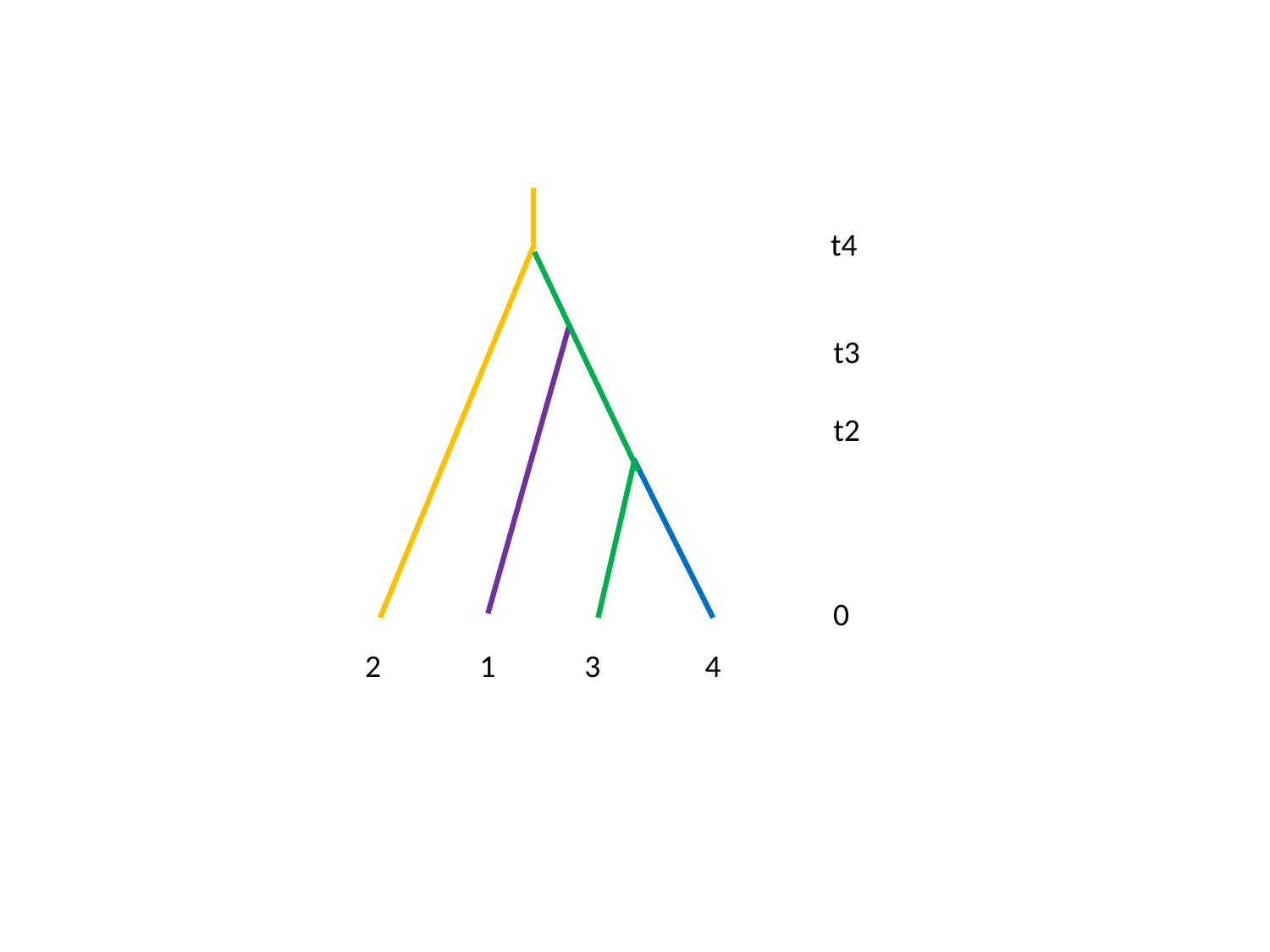

t4
t3
t2
0
2
1
3
4

## Slide 25
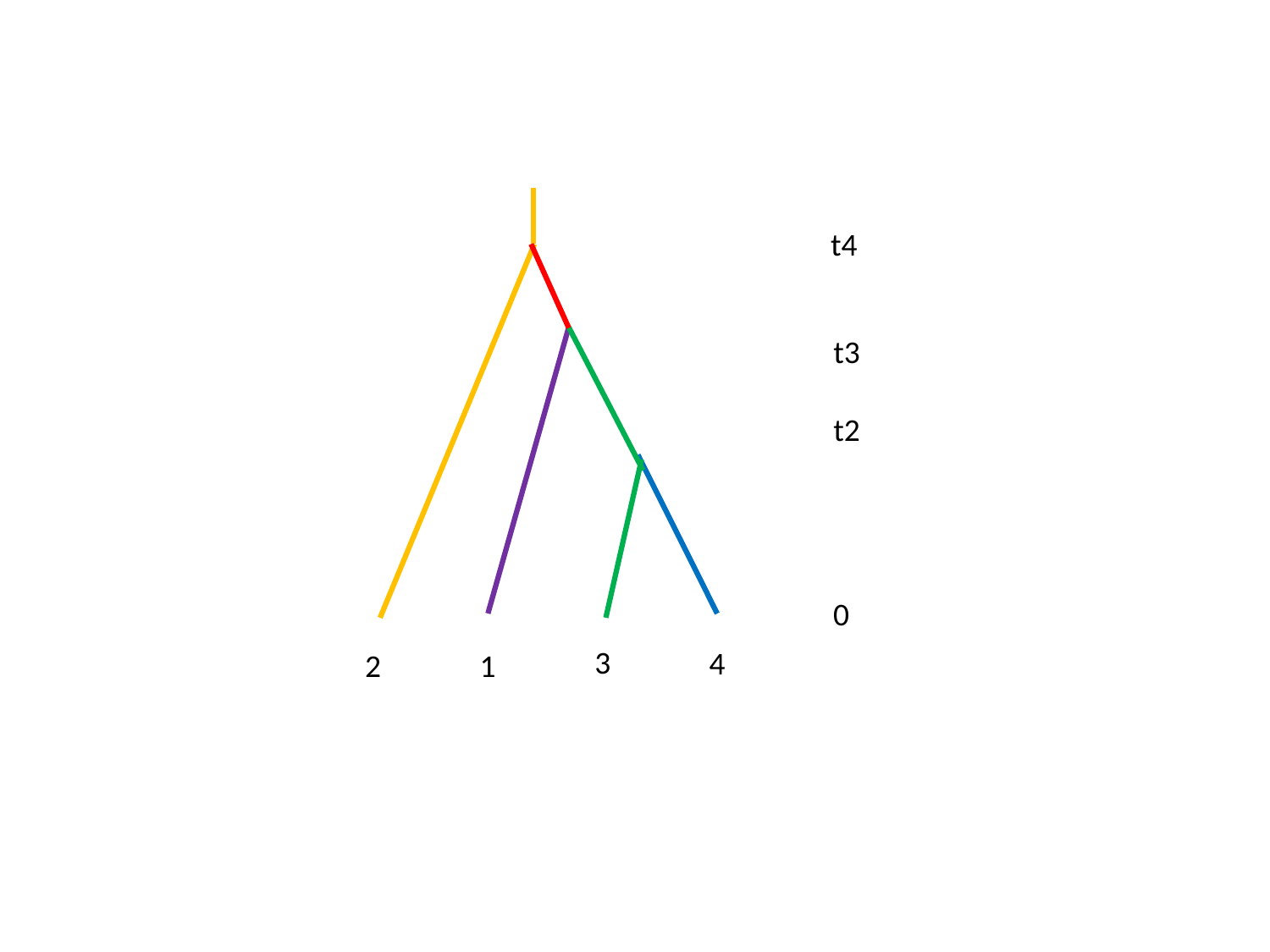

t4
t3
t2
0
3
4
2
1

## Slide 26
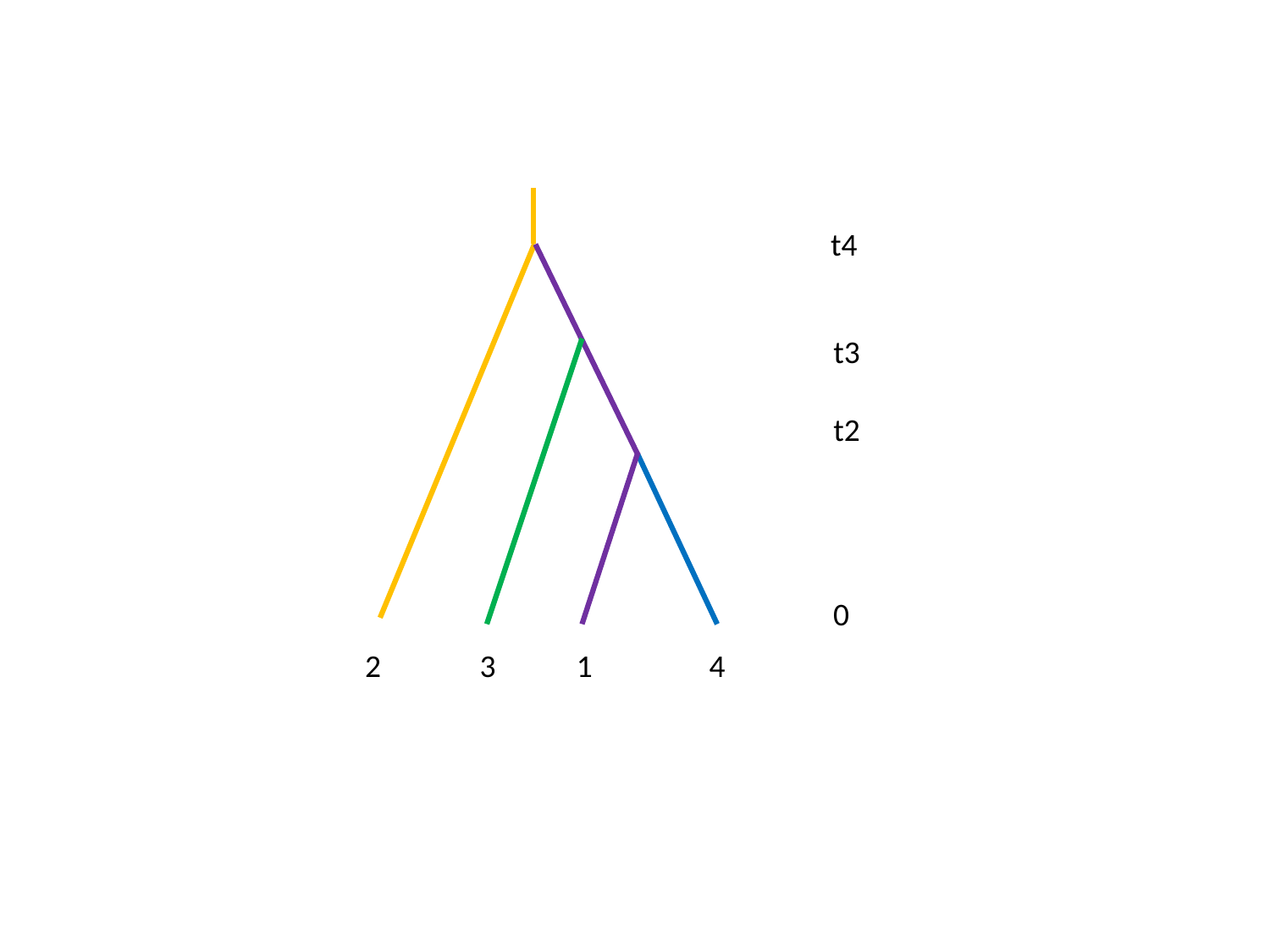

t4
t3
t2
0
2
3
1
4

## Slide 27
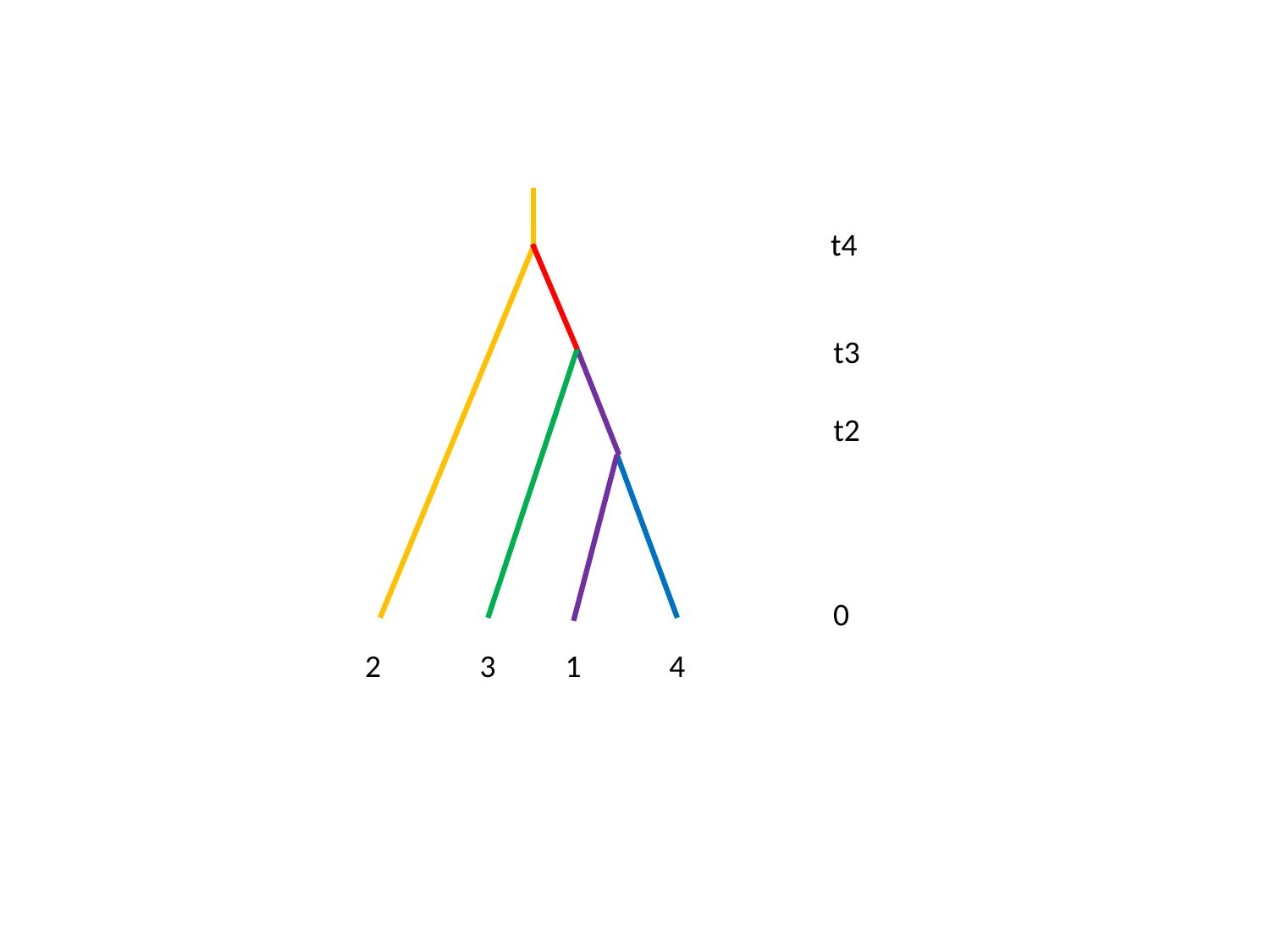

t4
t3
t2
0
2
3
1
4

## Slide 28
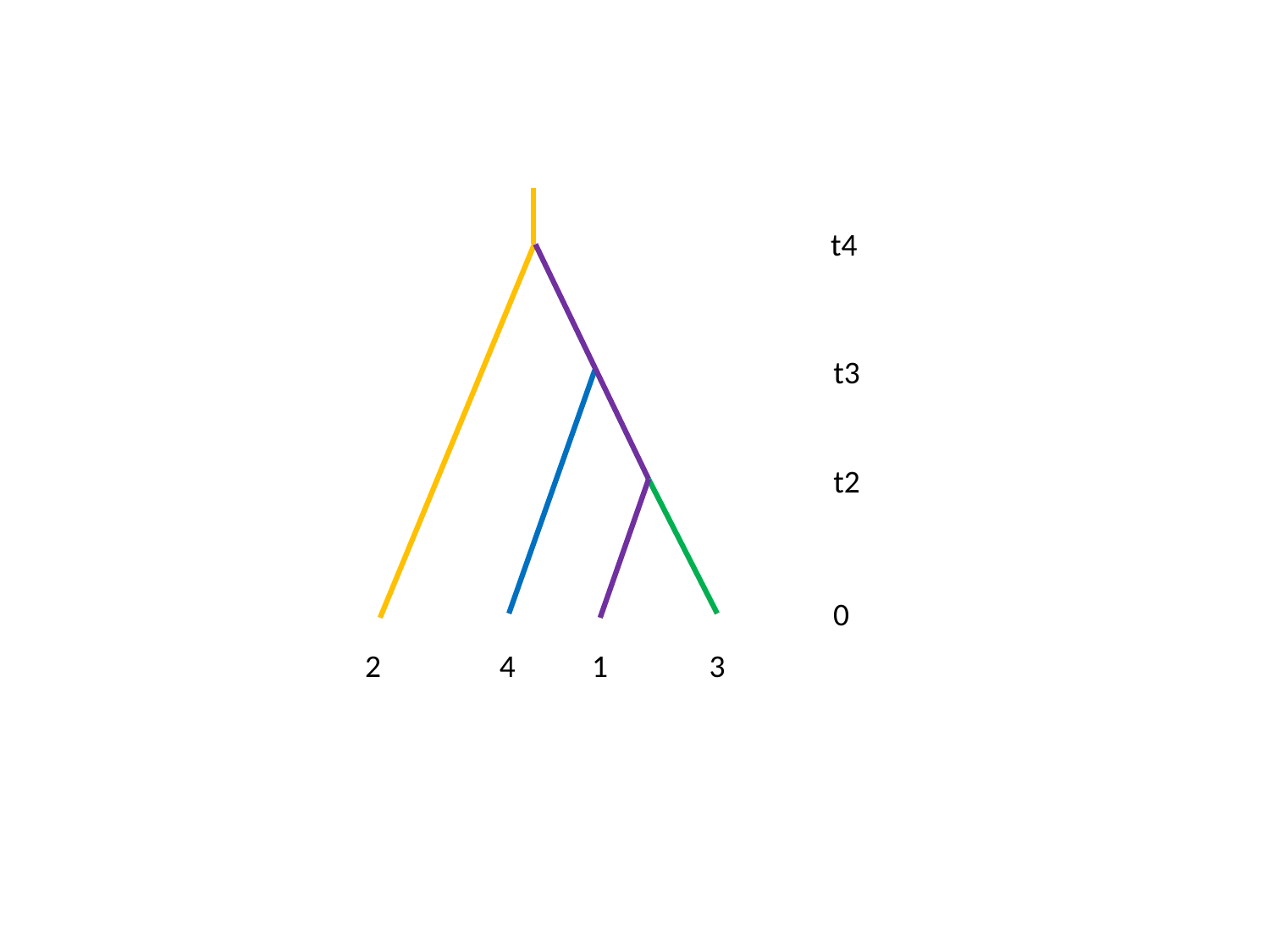

t4
t3
t2
0
2
4
1
3

## Slide 29
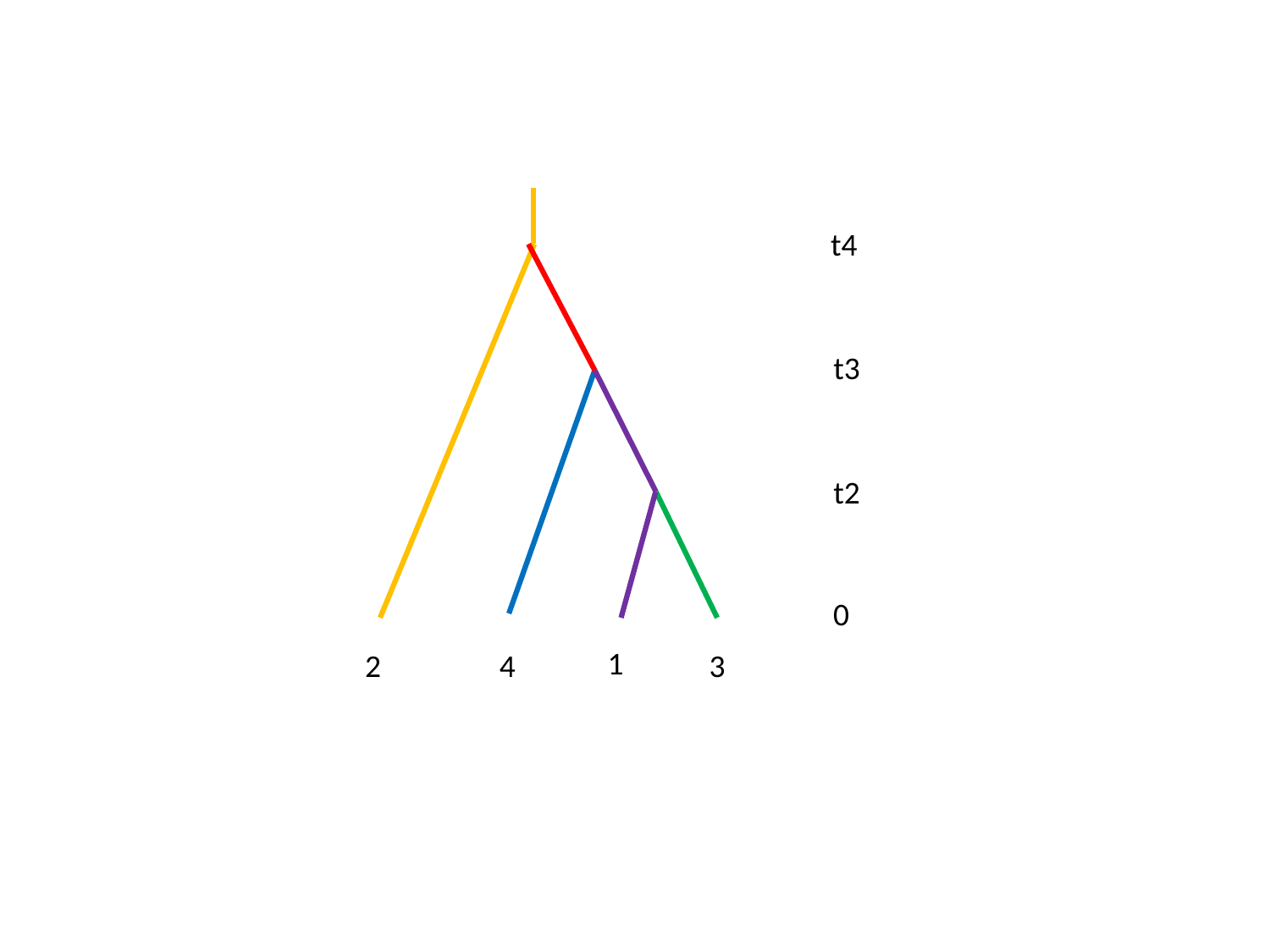

t4
t3
t2
0
1
2
4
3

## Slide 30
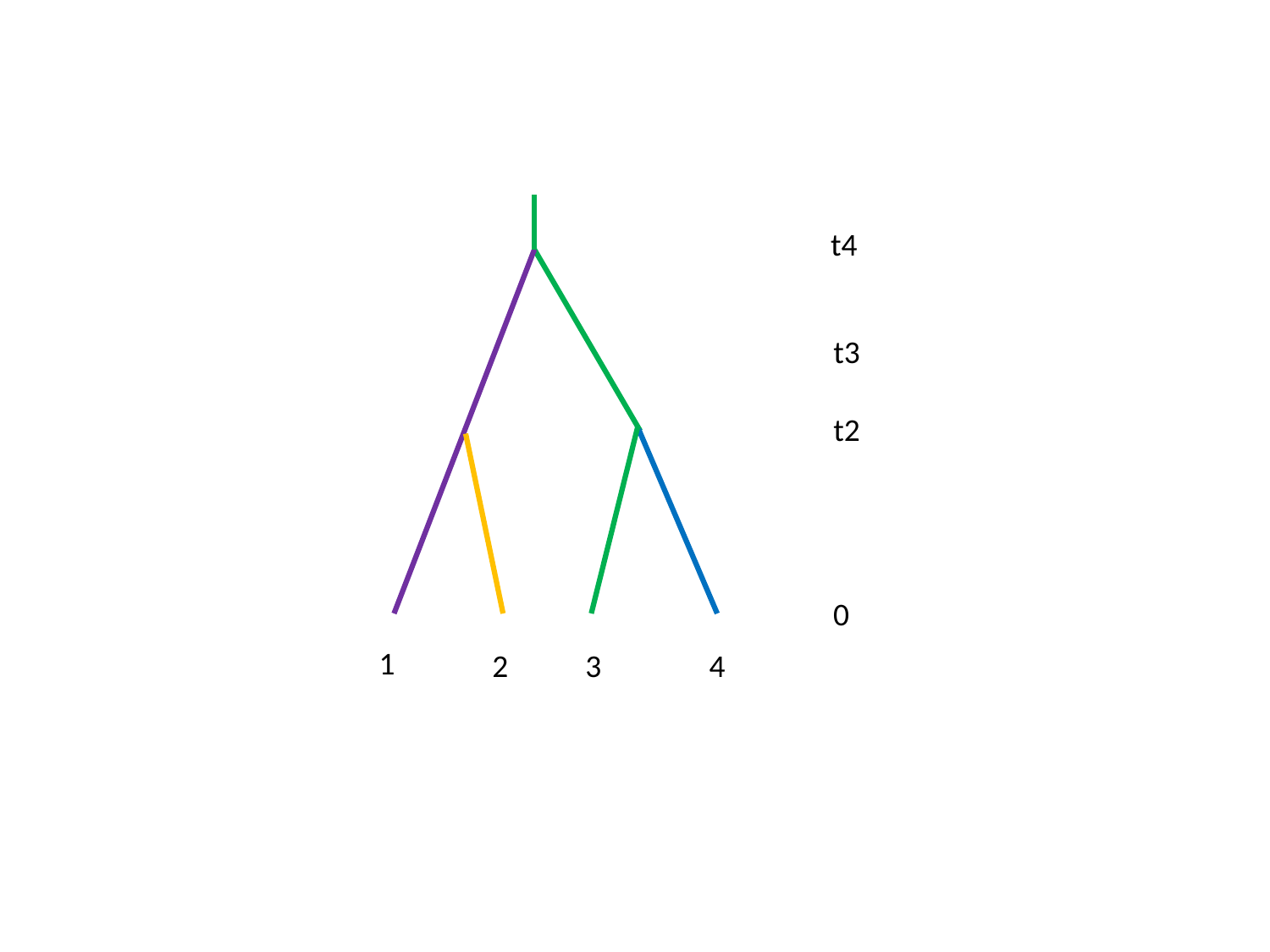

t4
t3
t2
0
1
2
3
4

## Slide 31
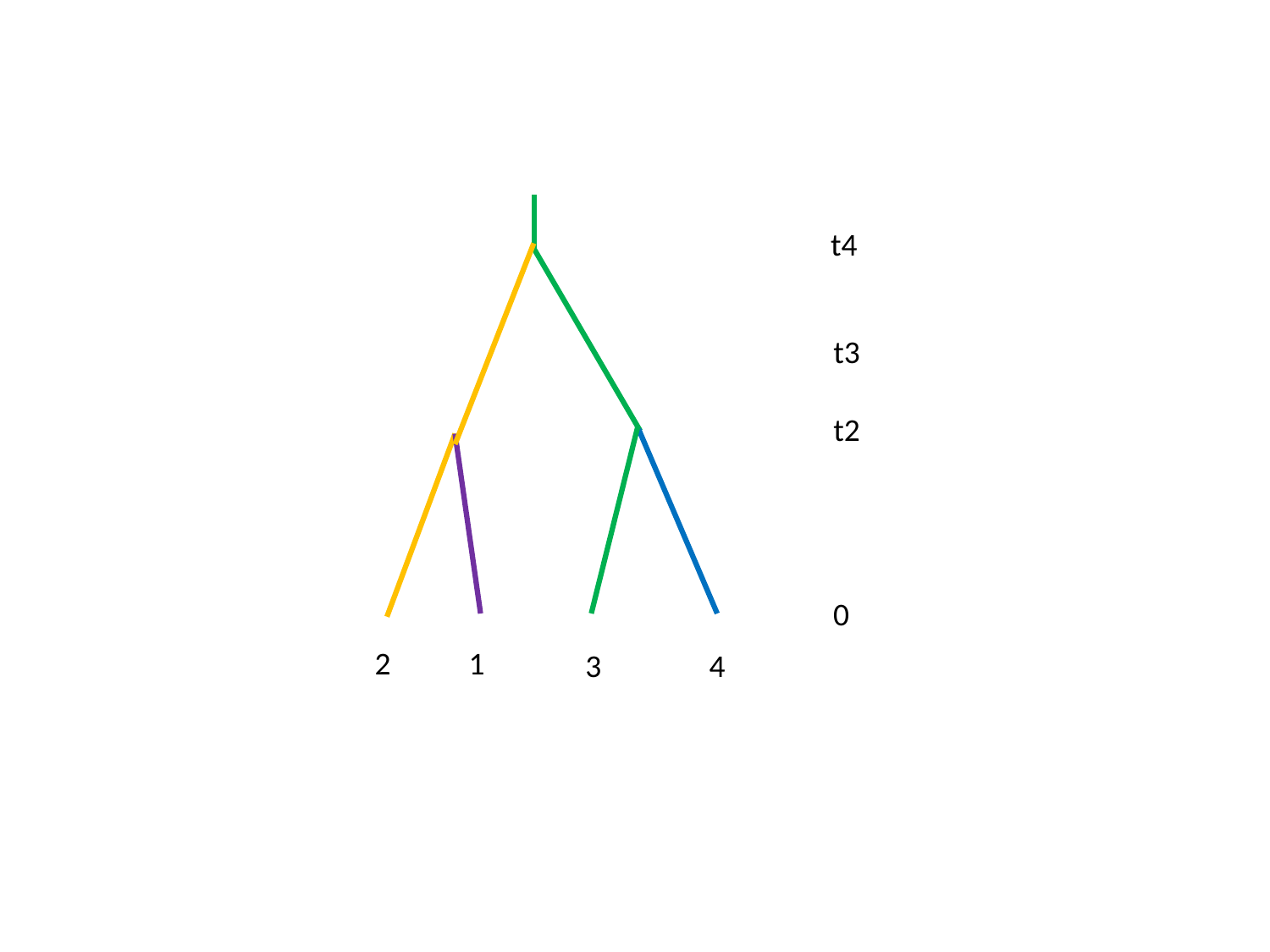

t4
t3
t2
0
2
1
3
4

## Slide 32
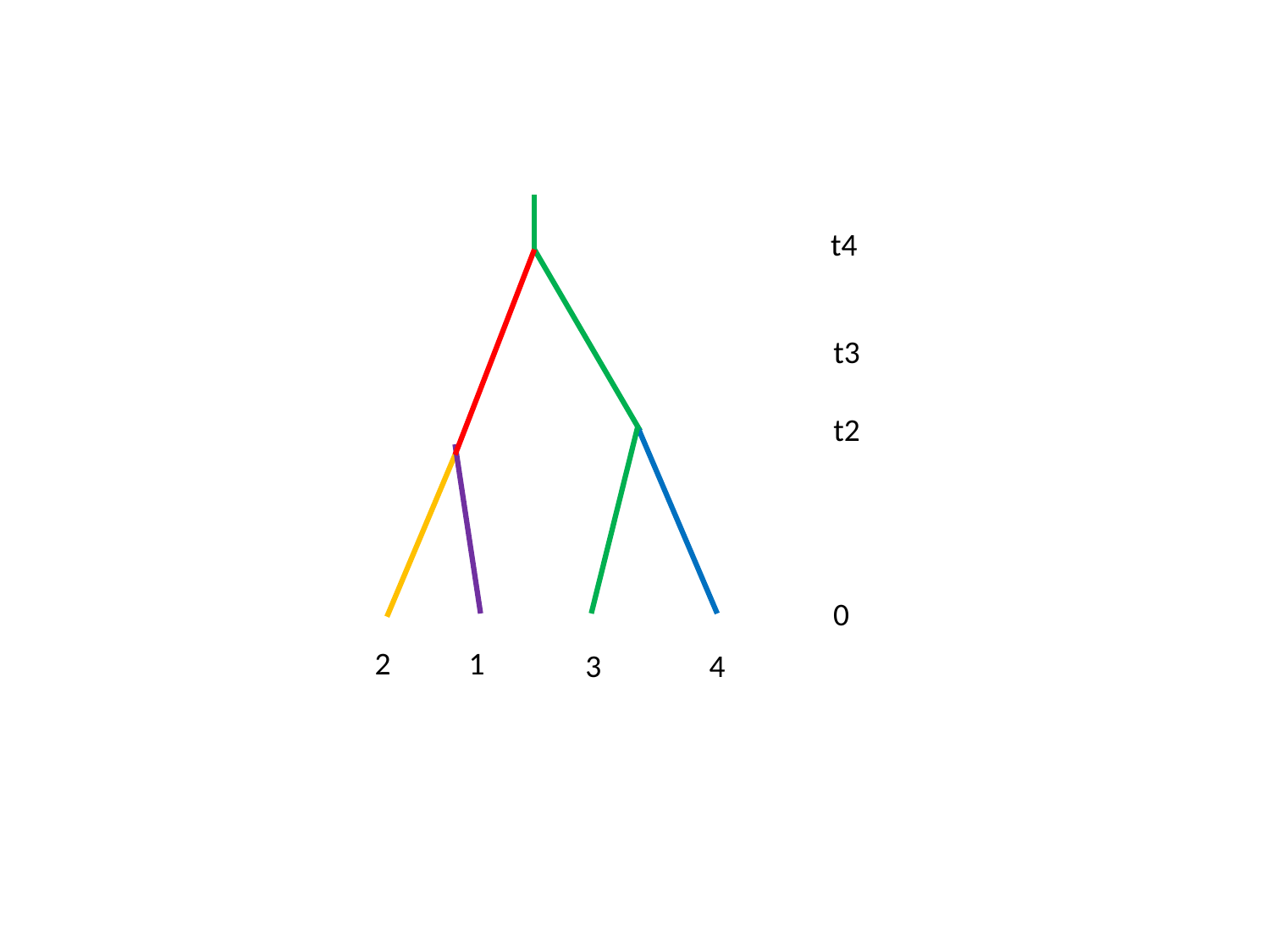

t4
t3
t2
0
2
1
3
4

## Slide 33
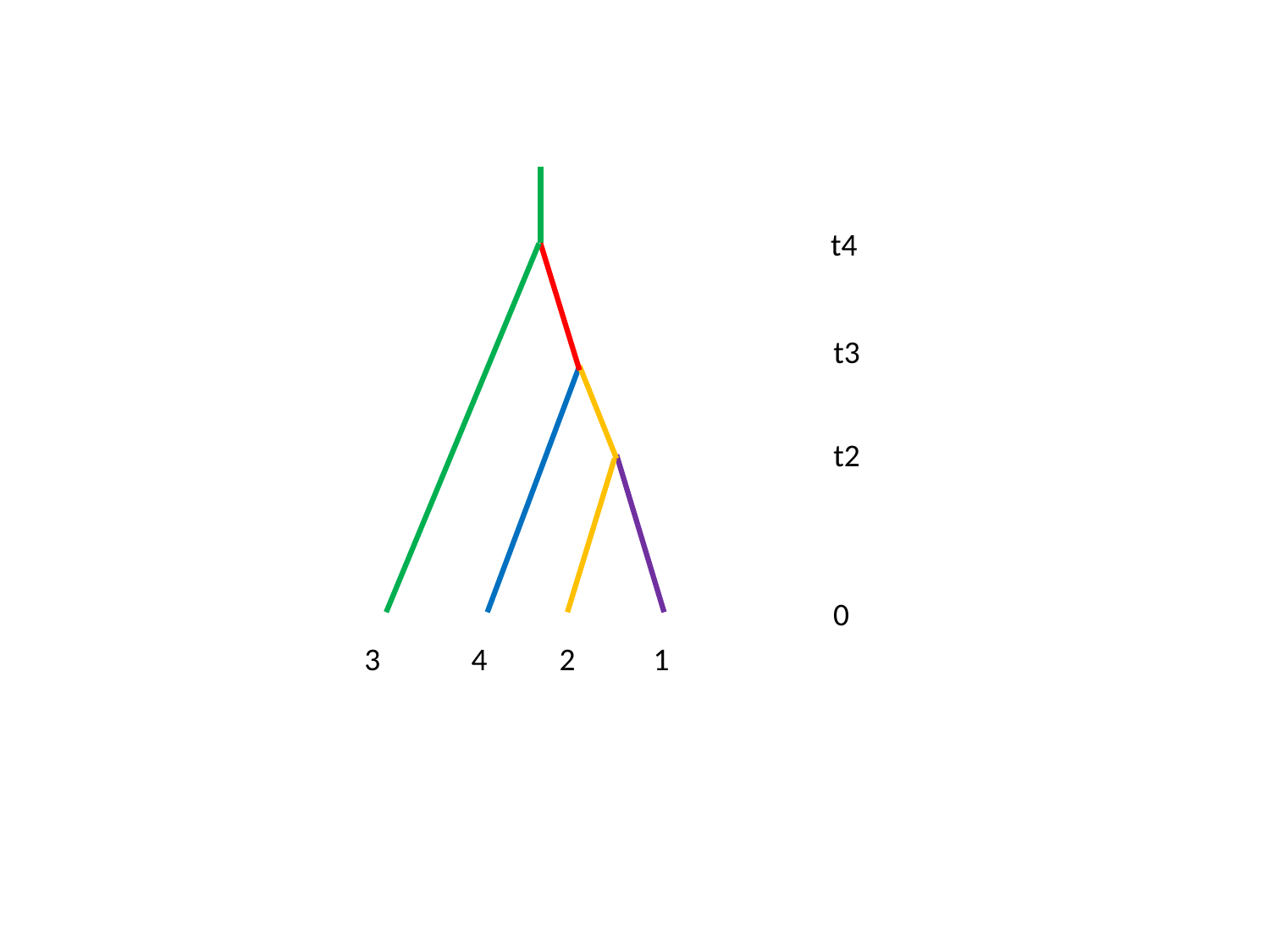

t4
t3
t2
0
3
4
2
1

## Slide 34
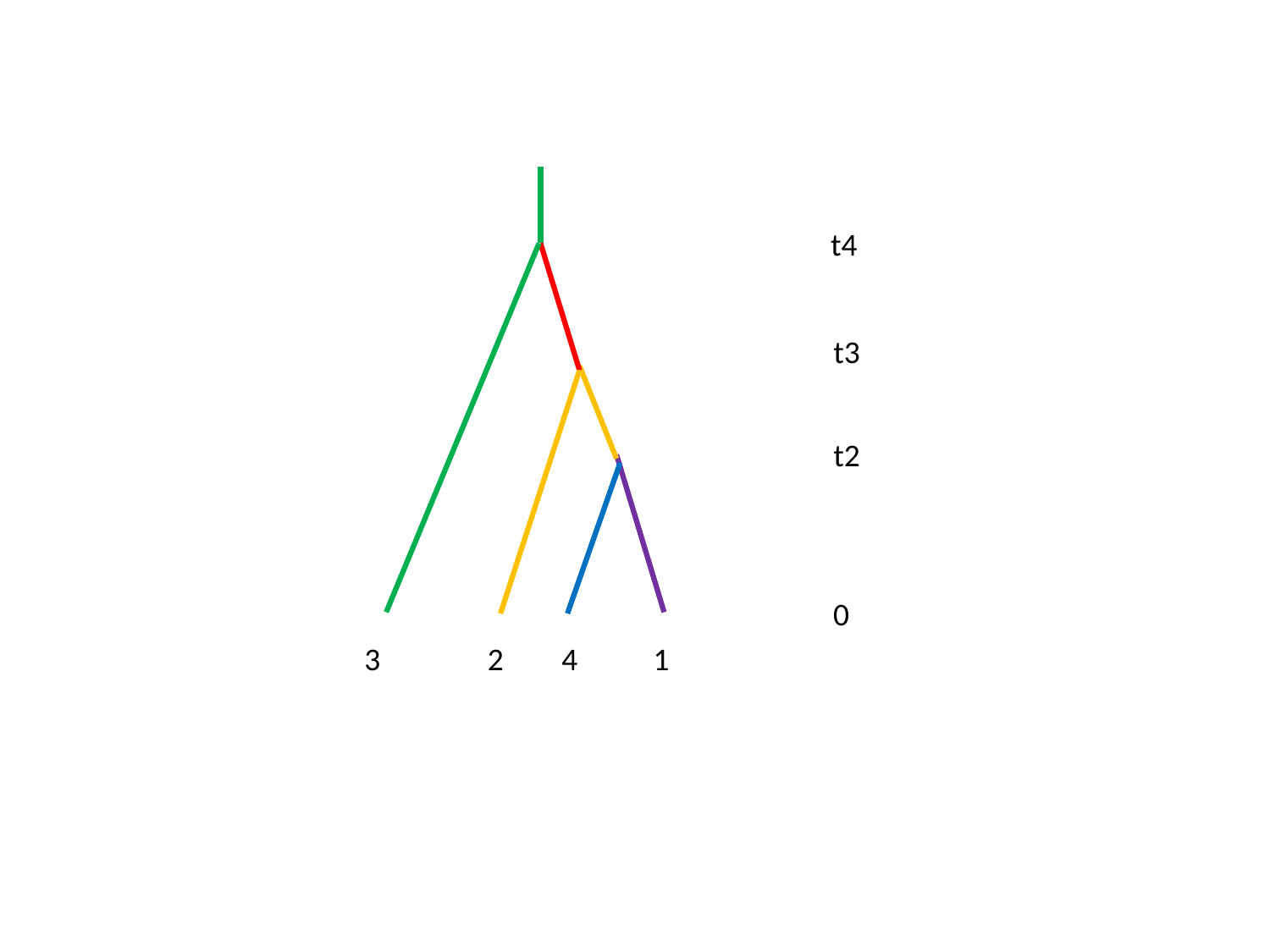

t4
t3
t2
0
3
2
4
1

## Slide 35
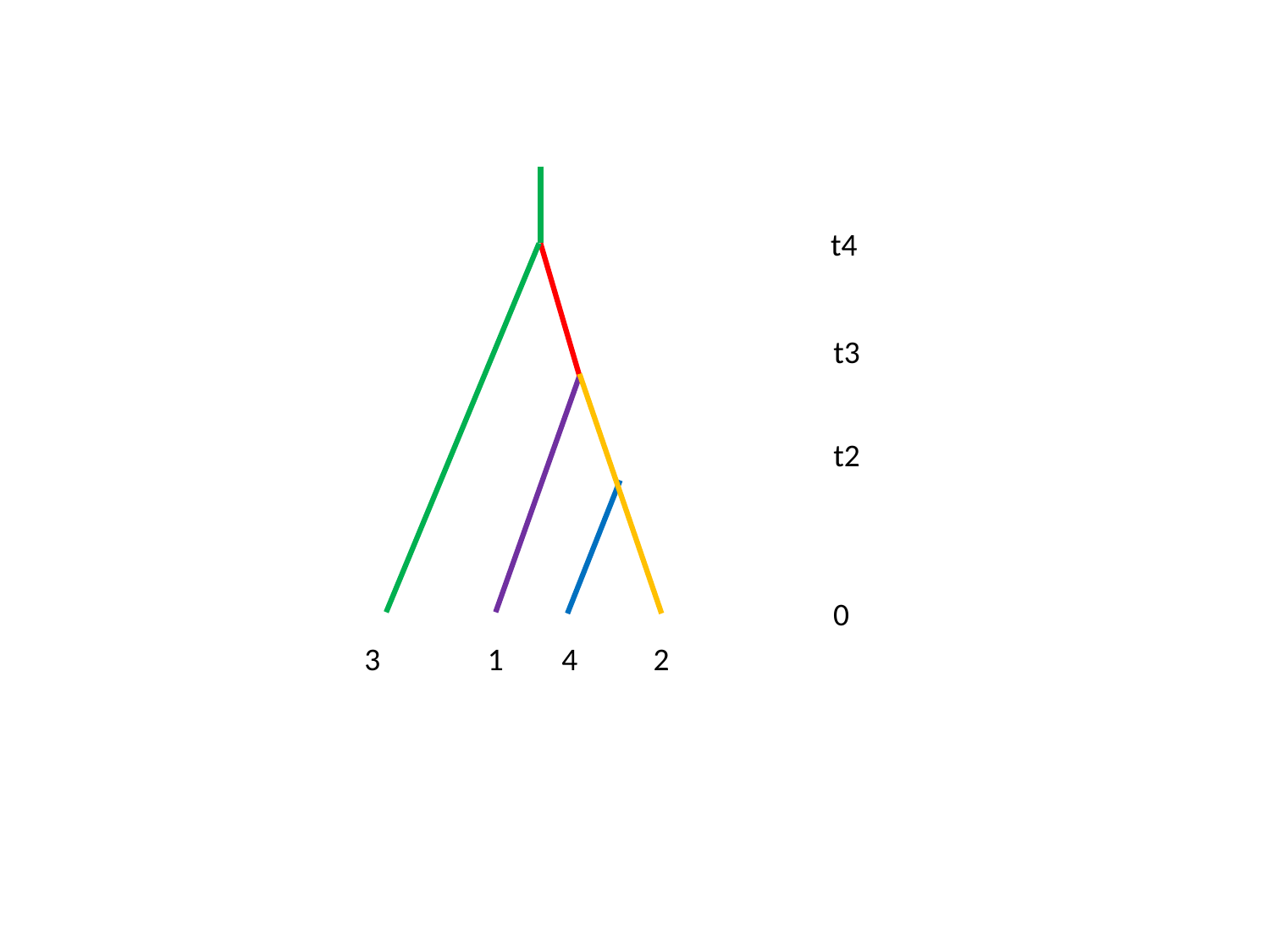

t4
t3
t2
0
3
1
4
2

## Slide 36
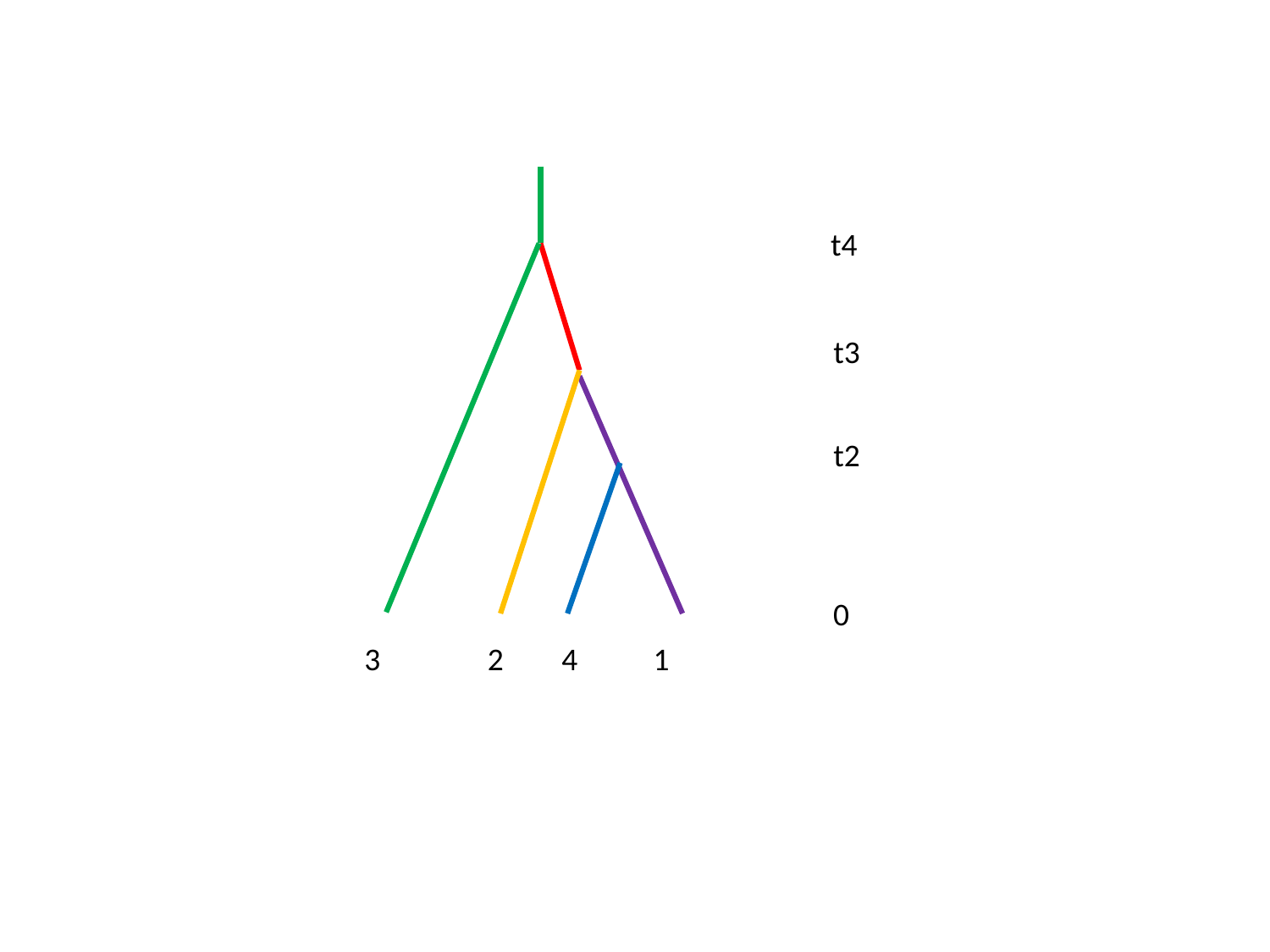

t4
t3
t2
0
3
2
4
1

## Slide 37
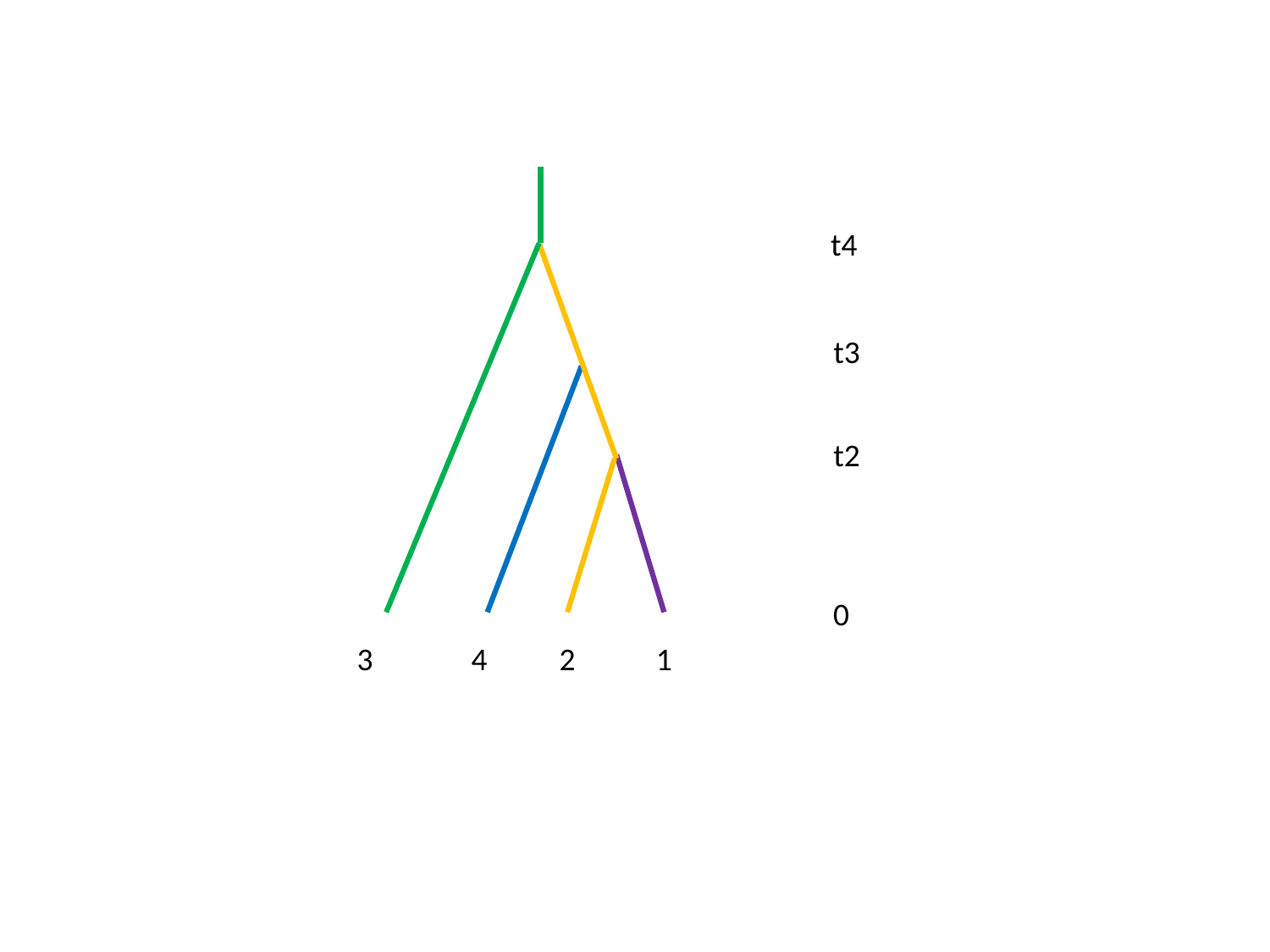

t4
t3
t2
0
3
4
2
1

## Slide 38
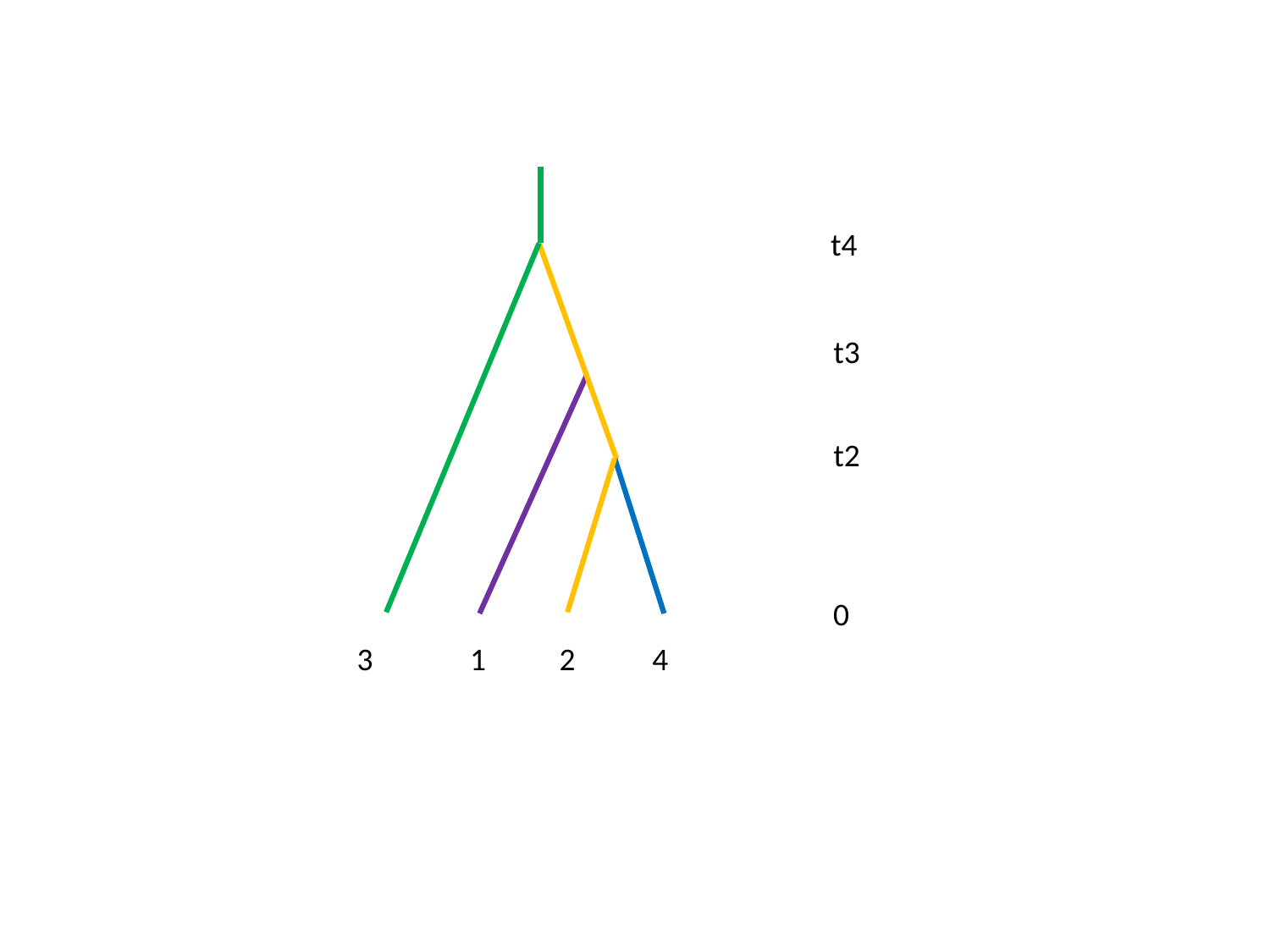

t4
t3
t2
0
3
1
2
4

## Slide 39
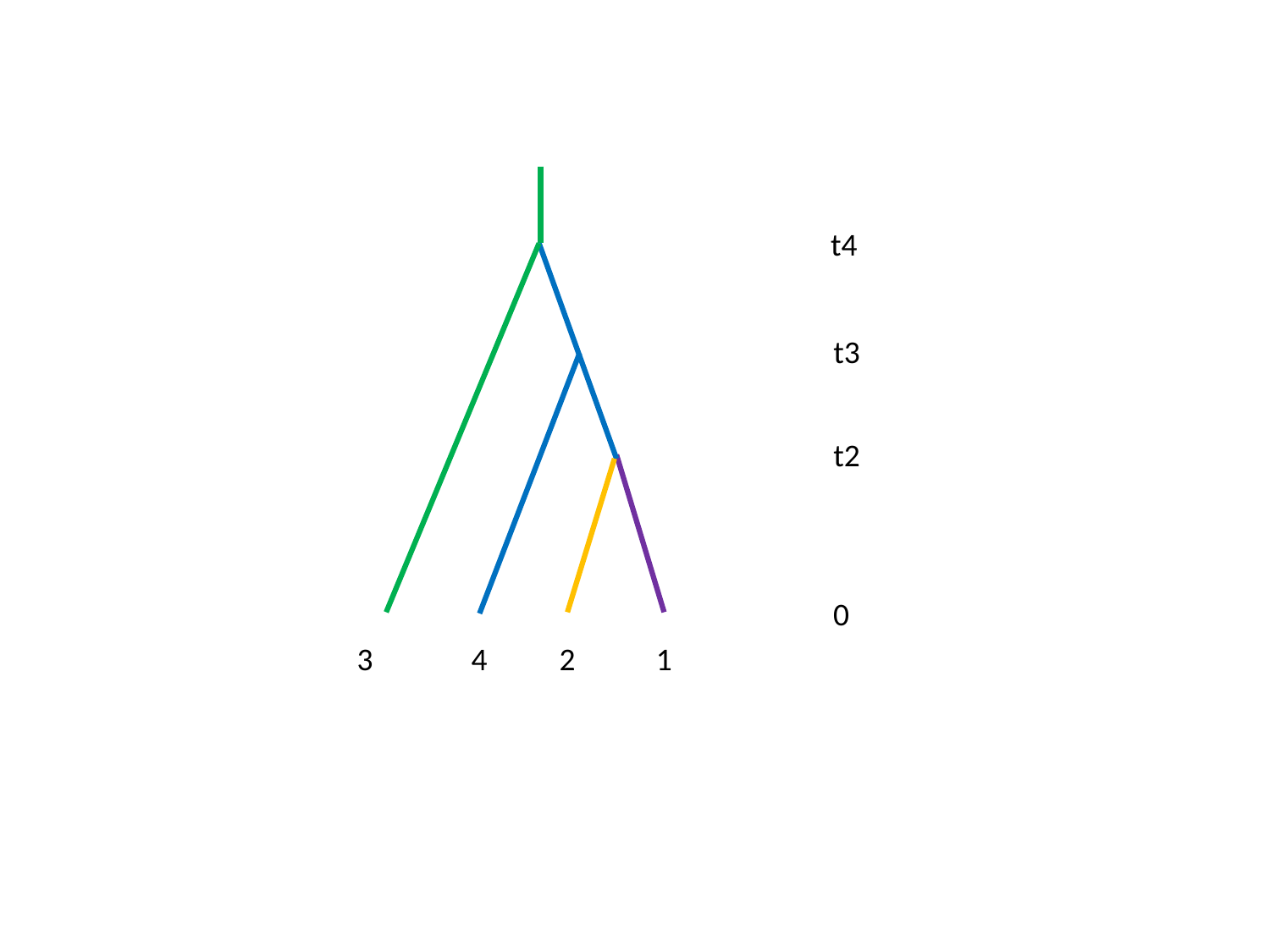

t4
t3
t2
0
3
4
2
1

## Slide 40
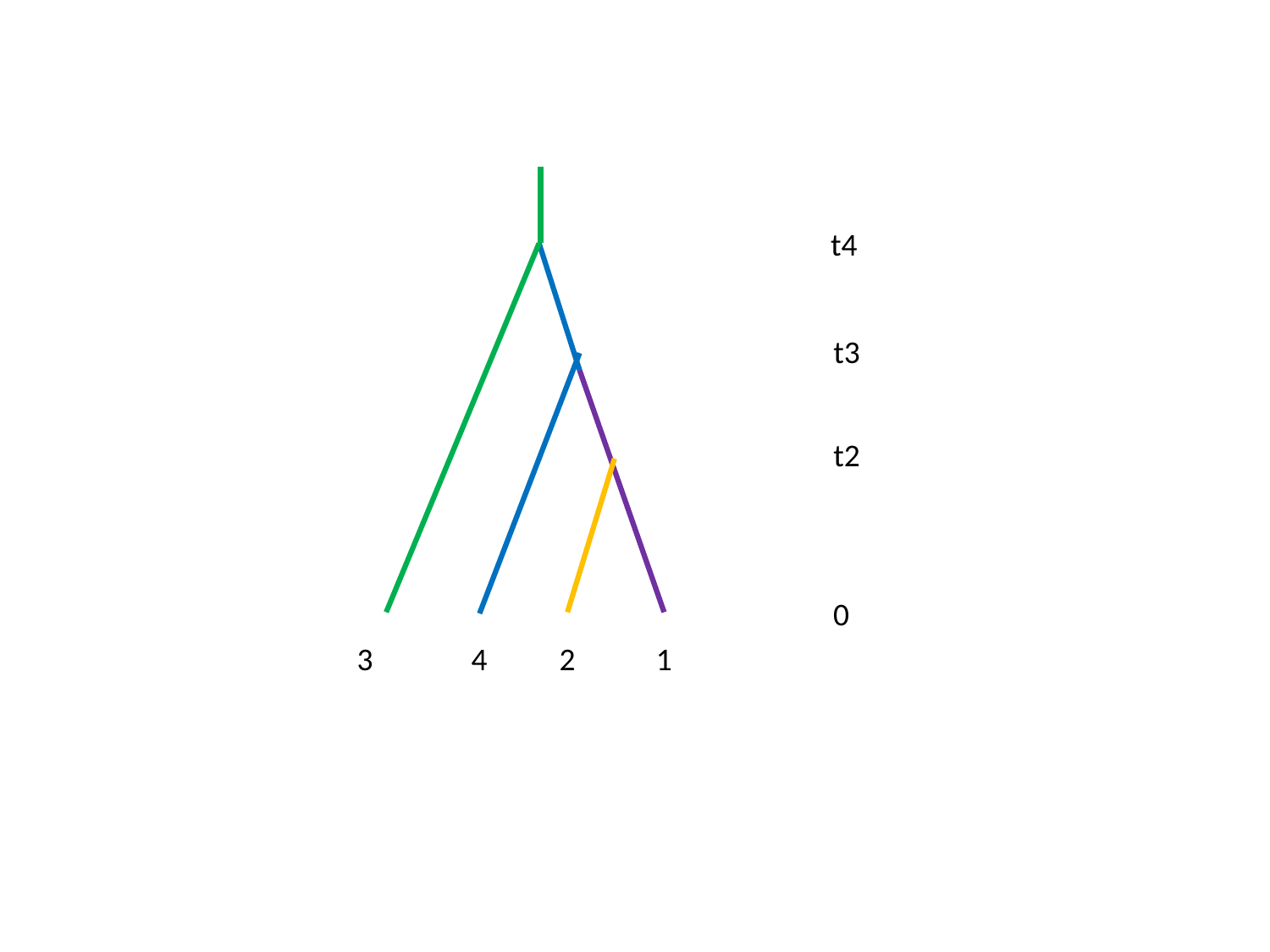

t4
t3
t2
0
3
4
2
1

## Slide 41
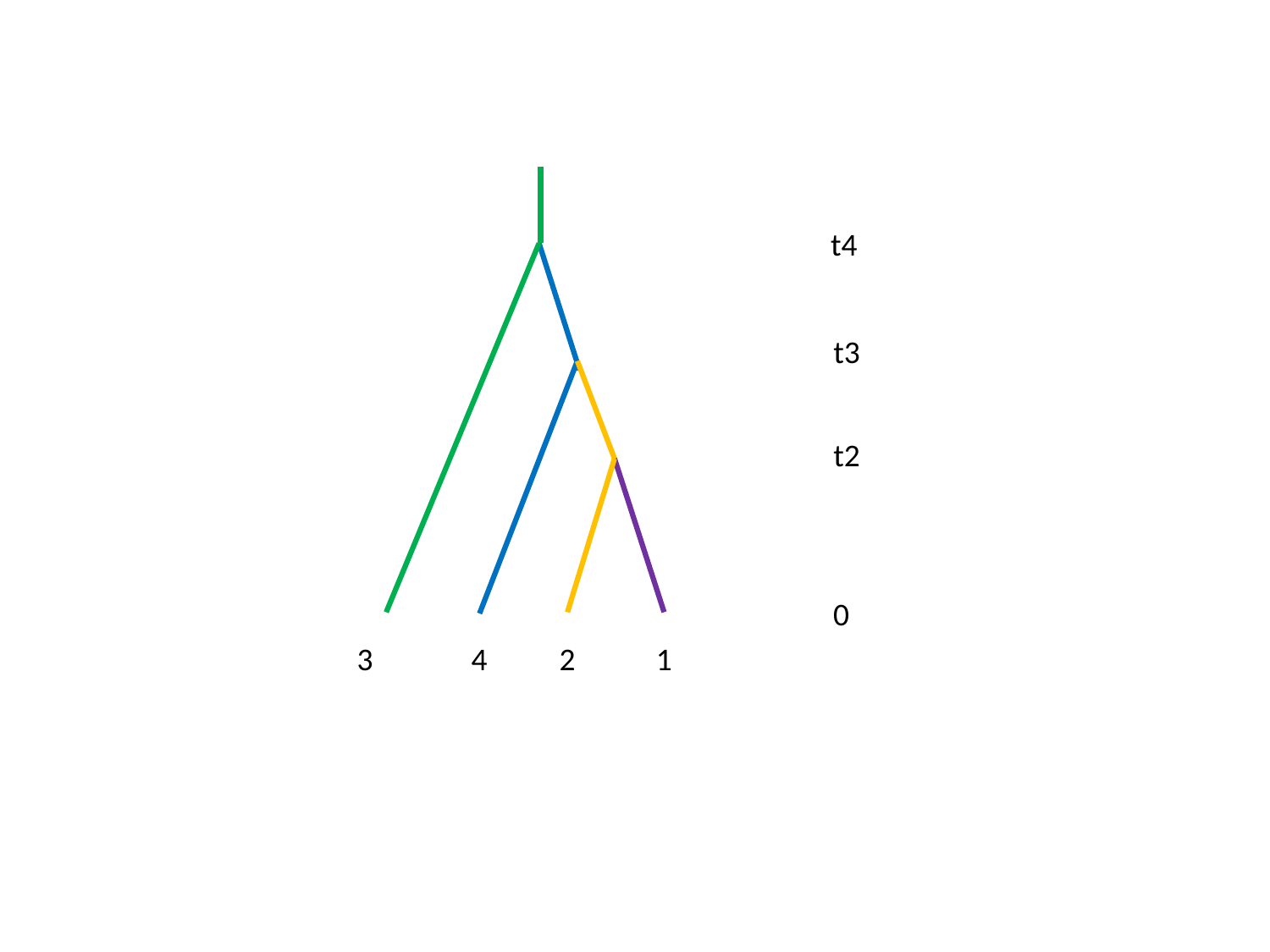

t4
t3
t2
0
3
4
2
1

## Slide 42
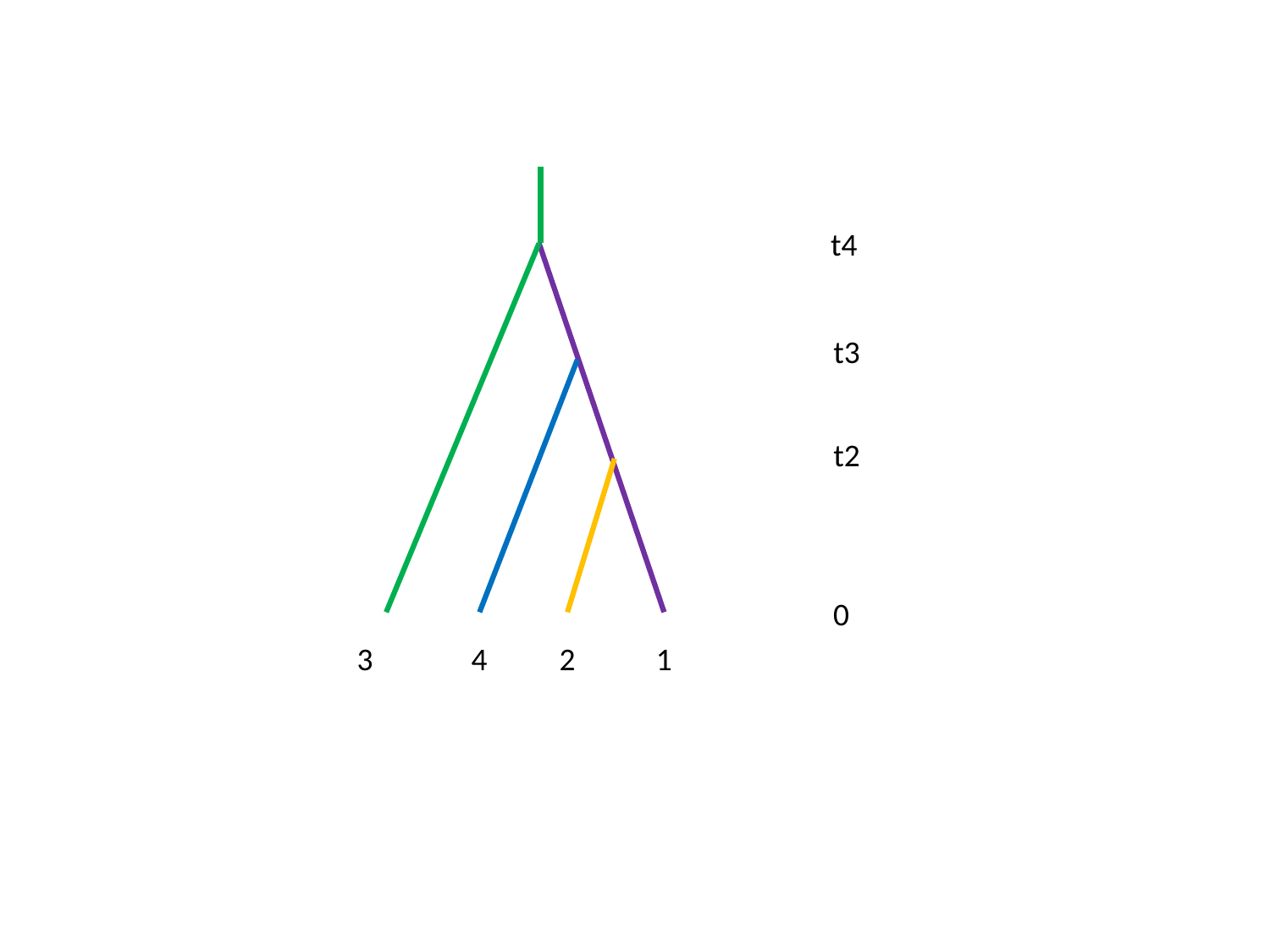

t4
t3
t2
0
3
4
2
1

## Slide 43
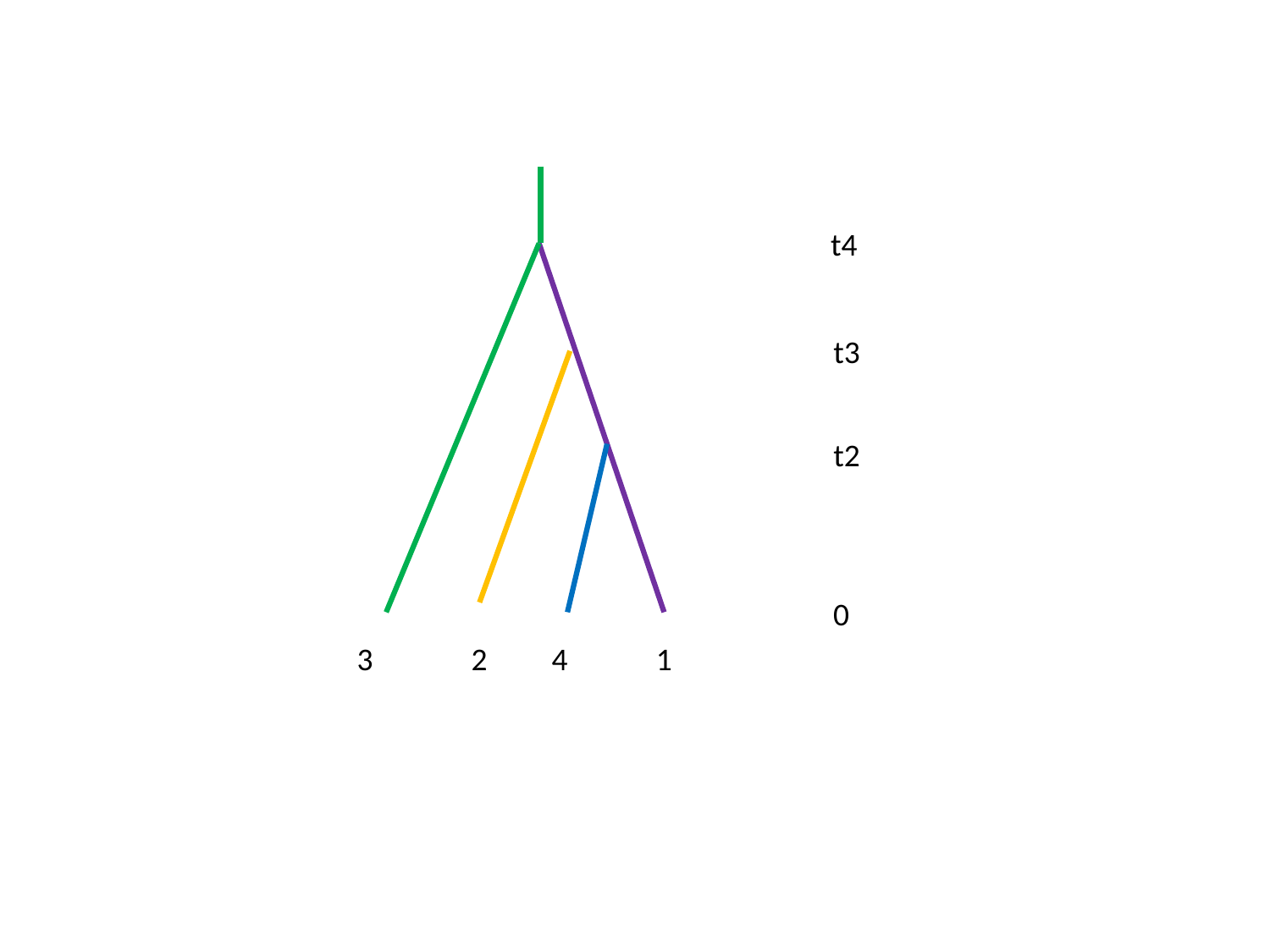

t4
t3
t2
0
3
2
4
1

## Slide 44
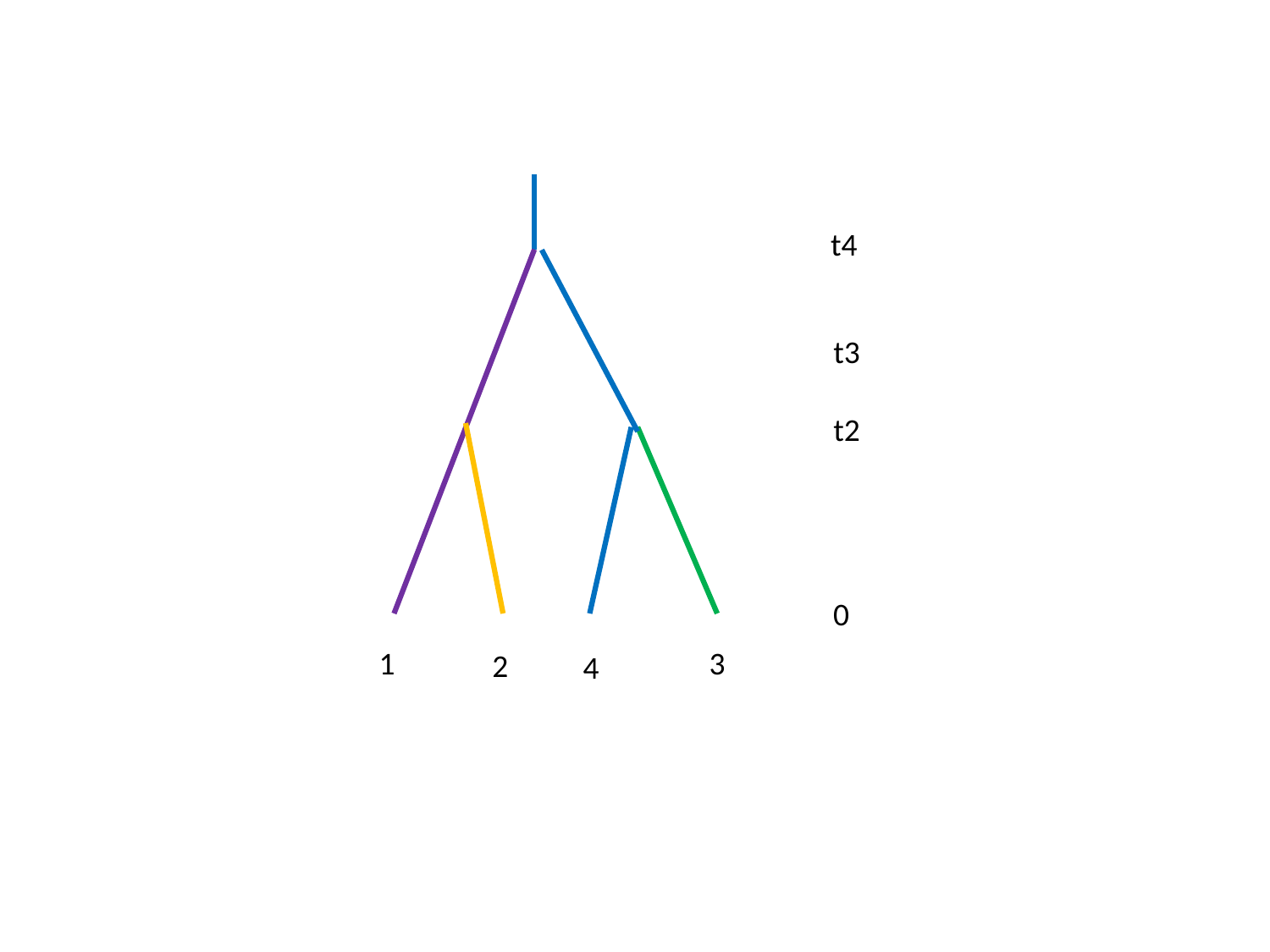

t4
t3
t2
0
1
3
2
4

## Slide 45
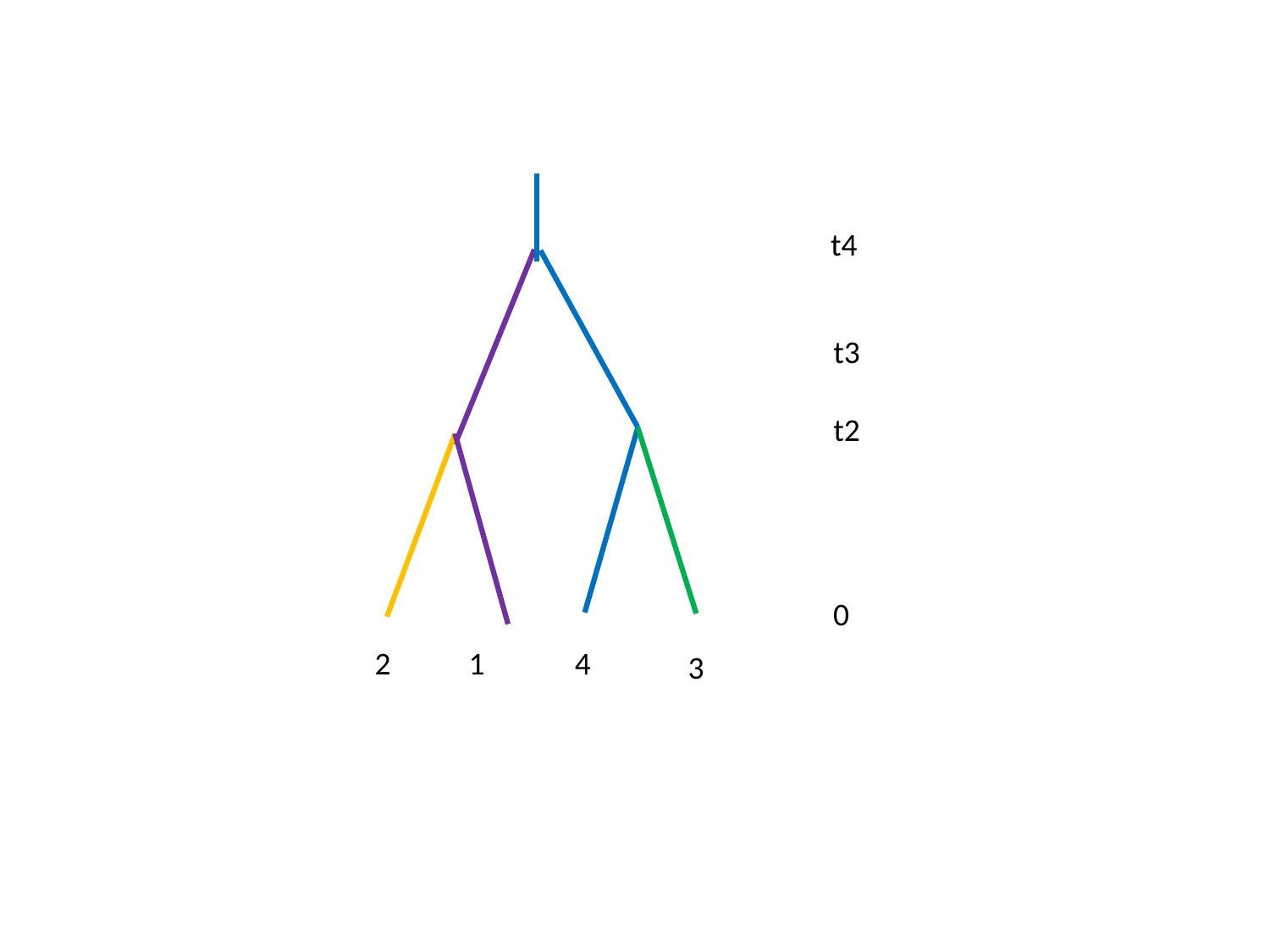

t4
t3
t2
0
2
1
4
3

## Slide 46
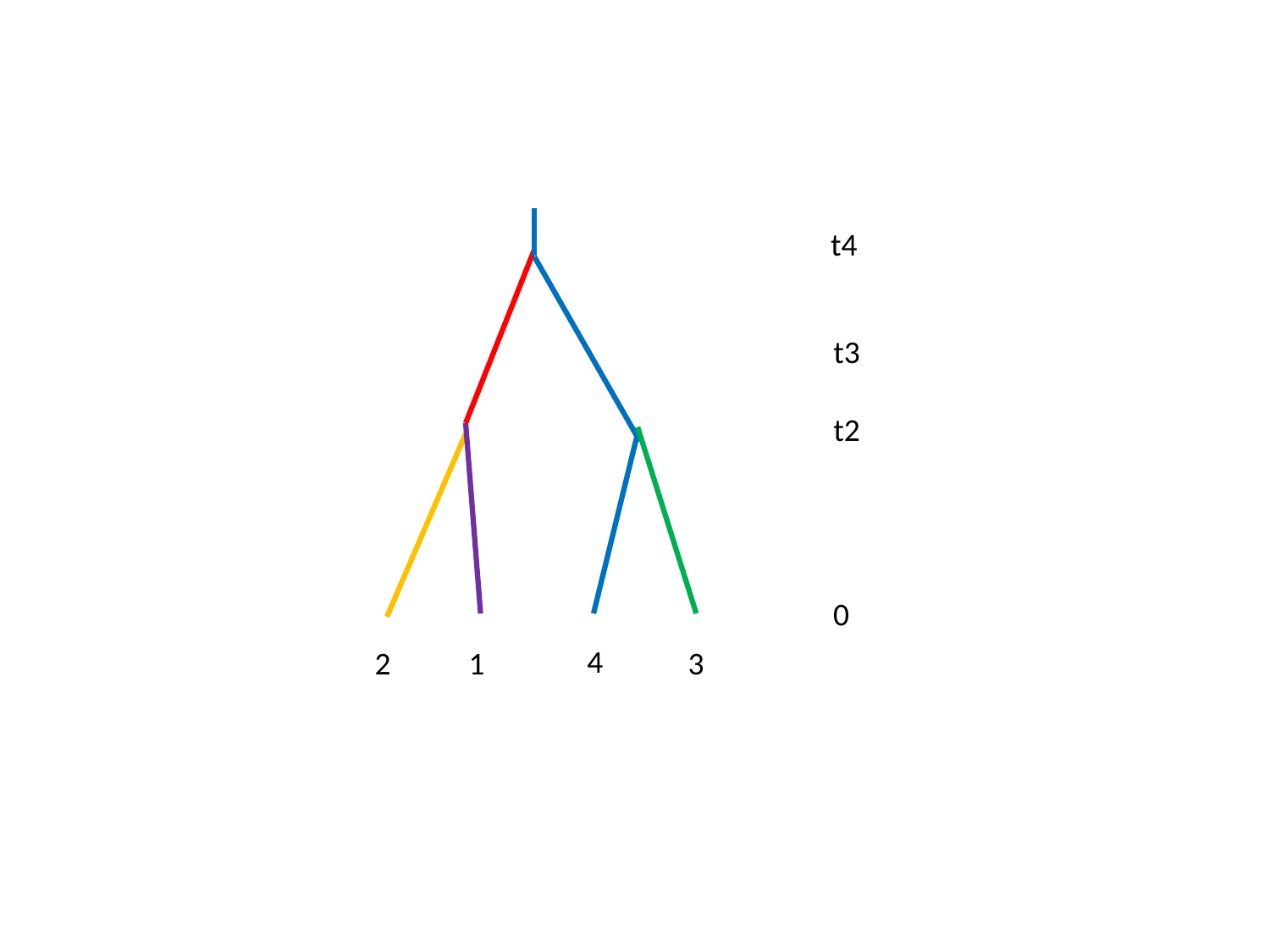

t4
t3
t2
0
4
2
1
3

## Slide 47
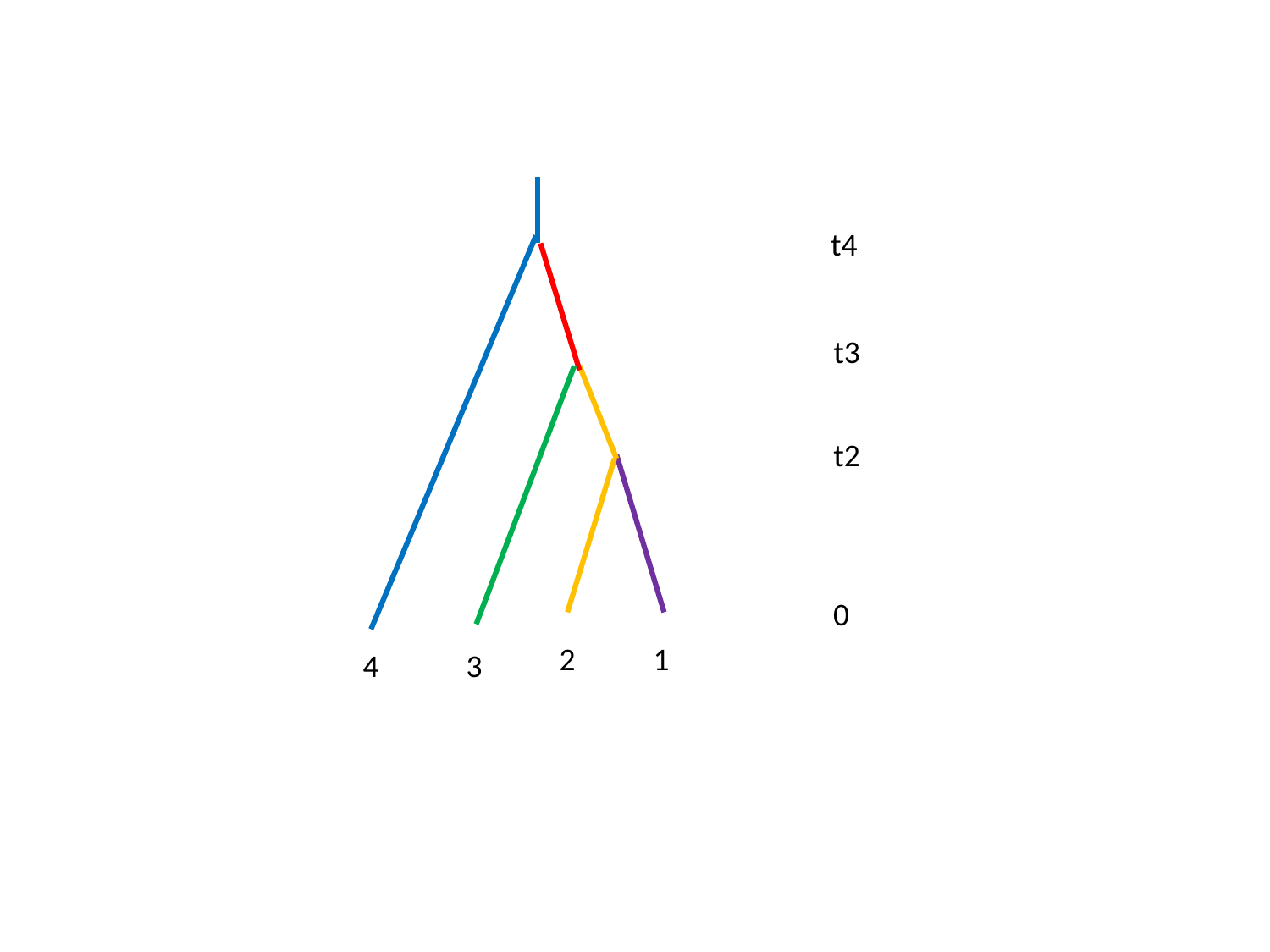

t4
t3
t2
0
2
1
4
3

## Slide 48
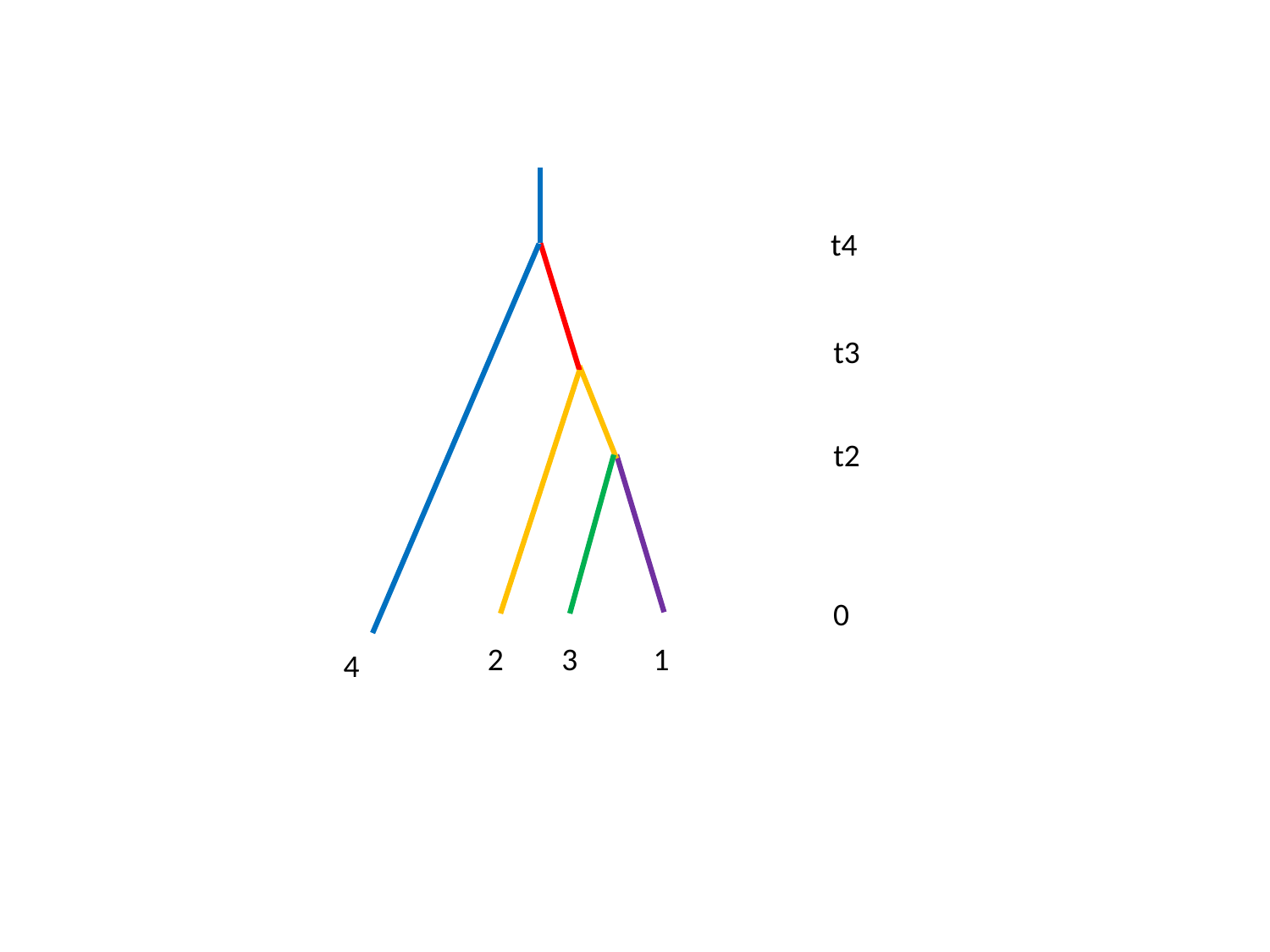

t4
t3
t2
0
2
3
1
4

## Slide 49
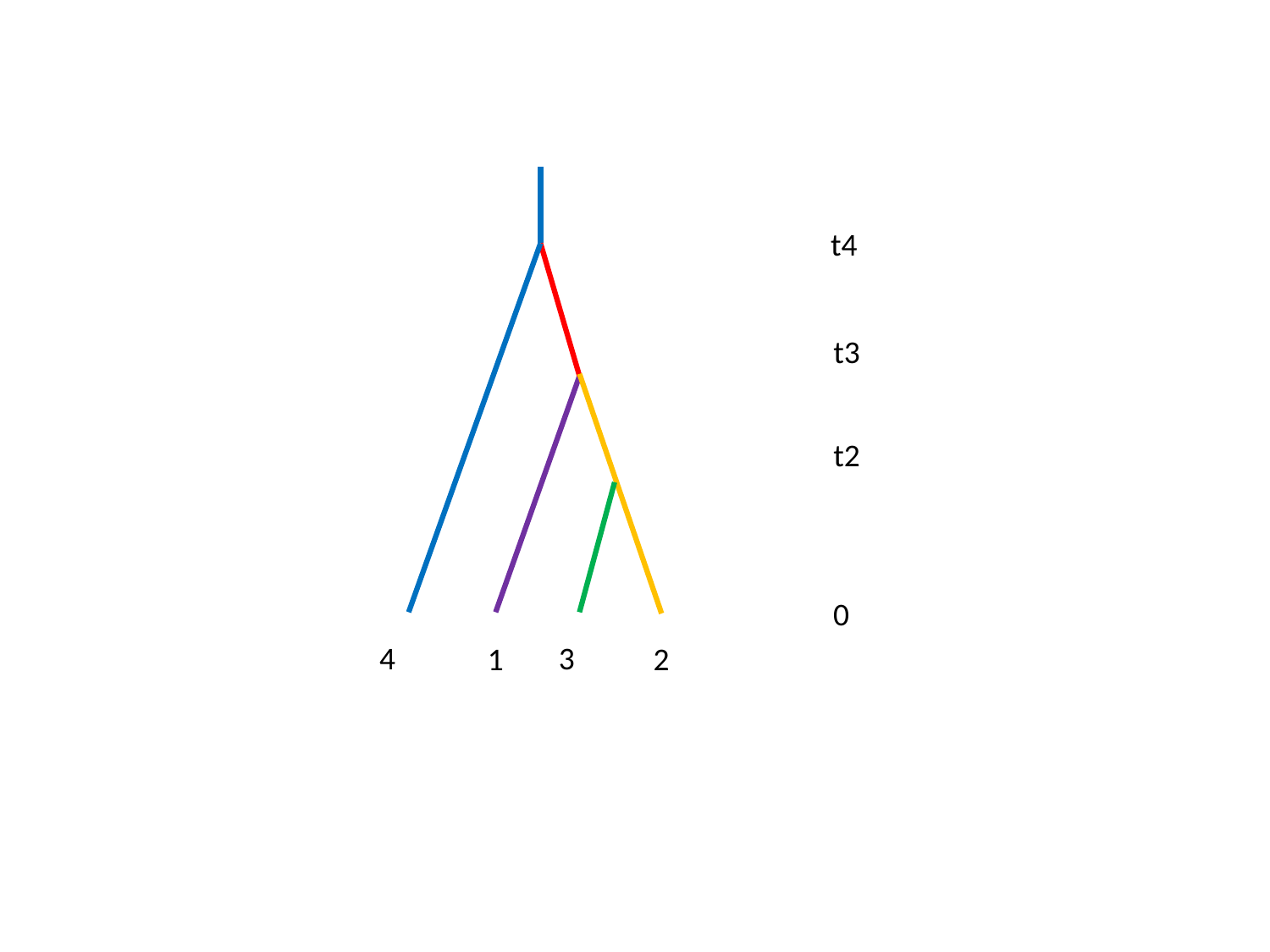

t4
t3
t2
0
4
3
1
2

## Slide 50
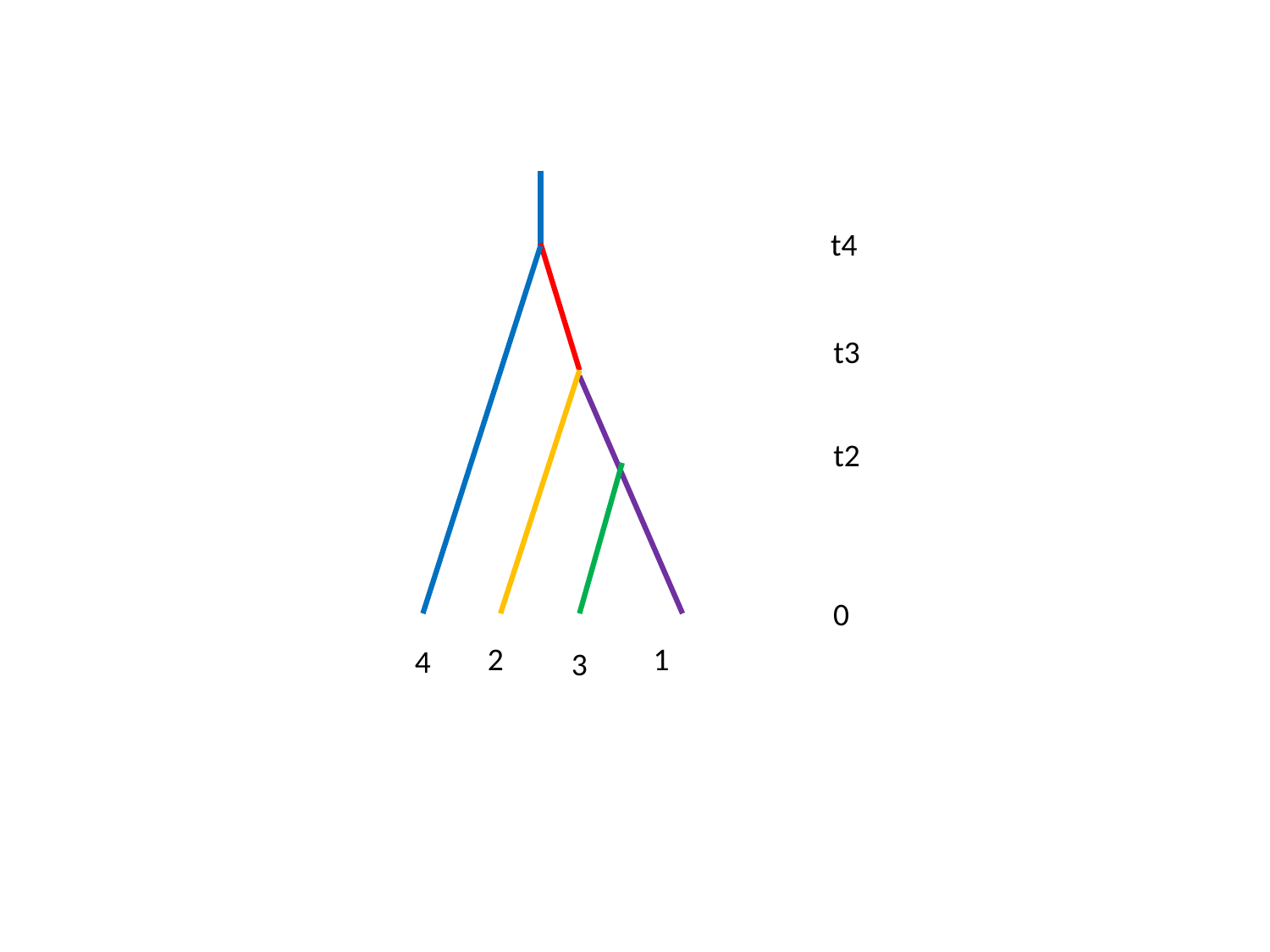

t4
t3
t2
0
2
1
4
3

## Slide 51
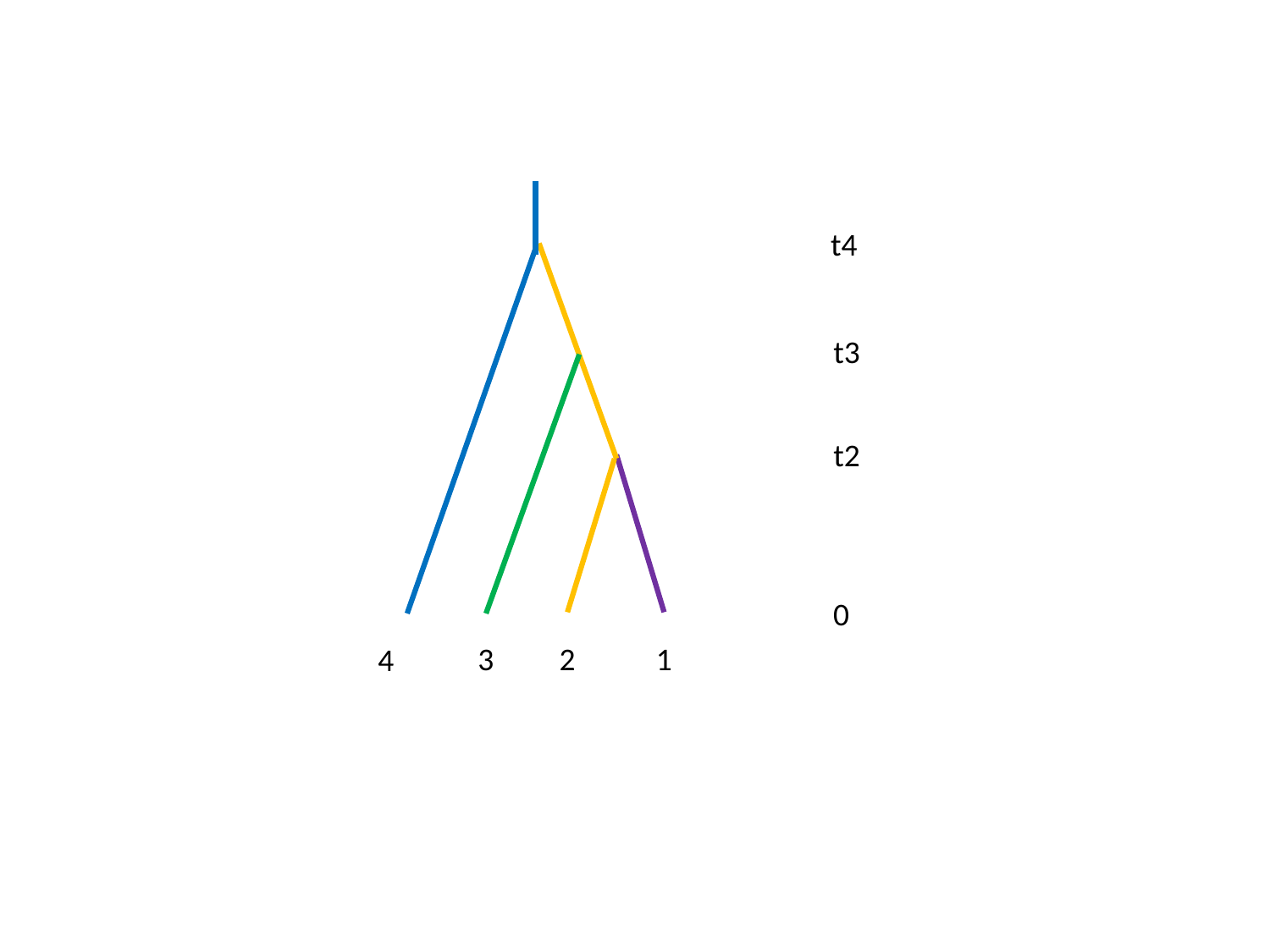

t4
t3
t2
0
3
2
1
4

## Slide 52
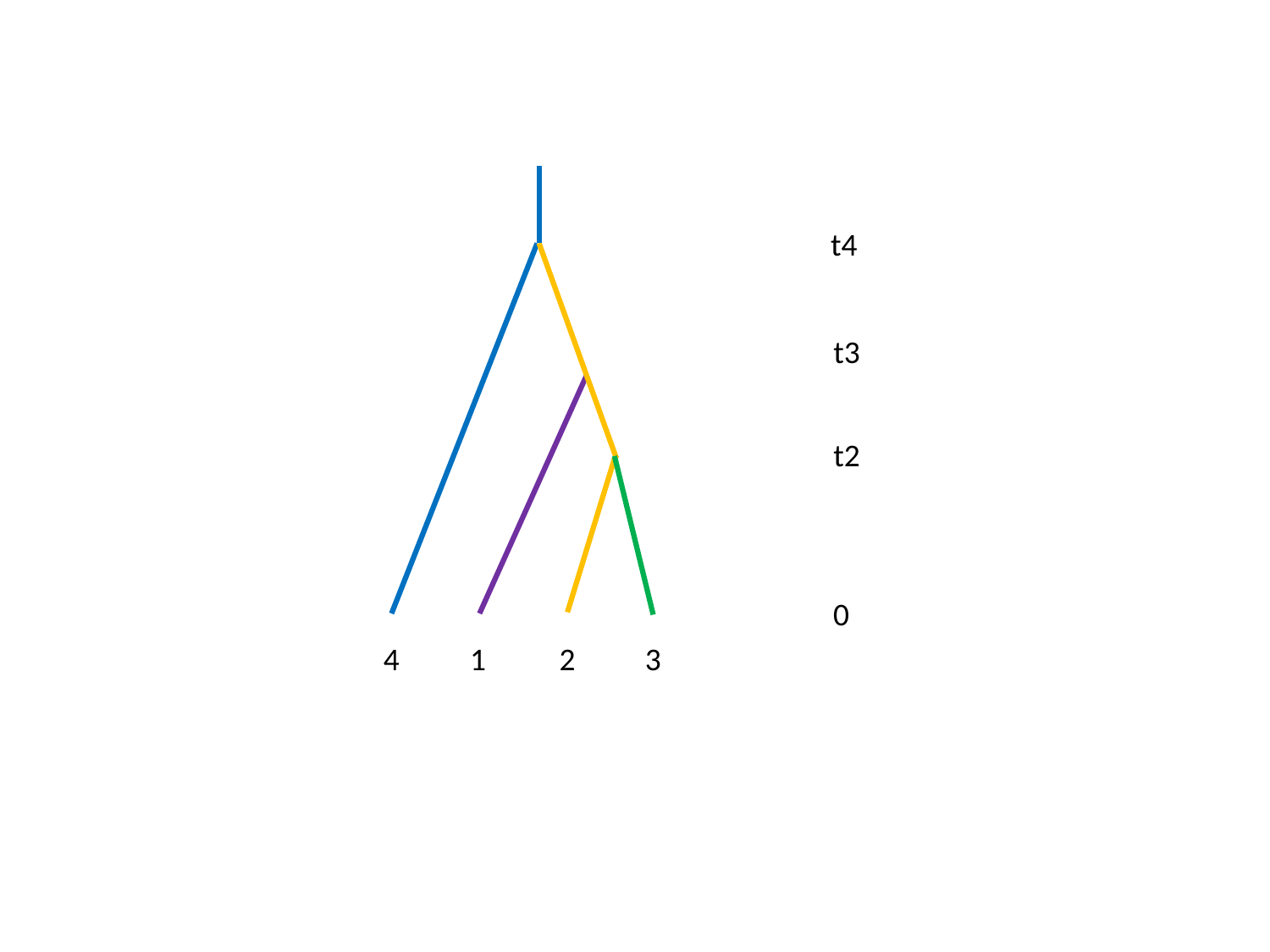

t4
t3
t2
0
4
1
2
3

## Slide 53
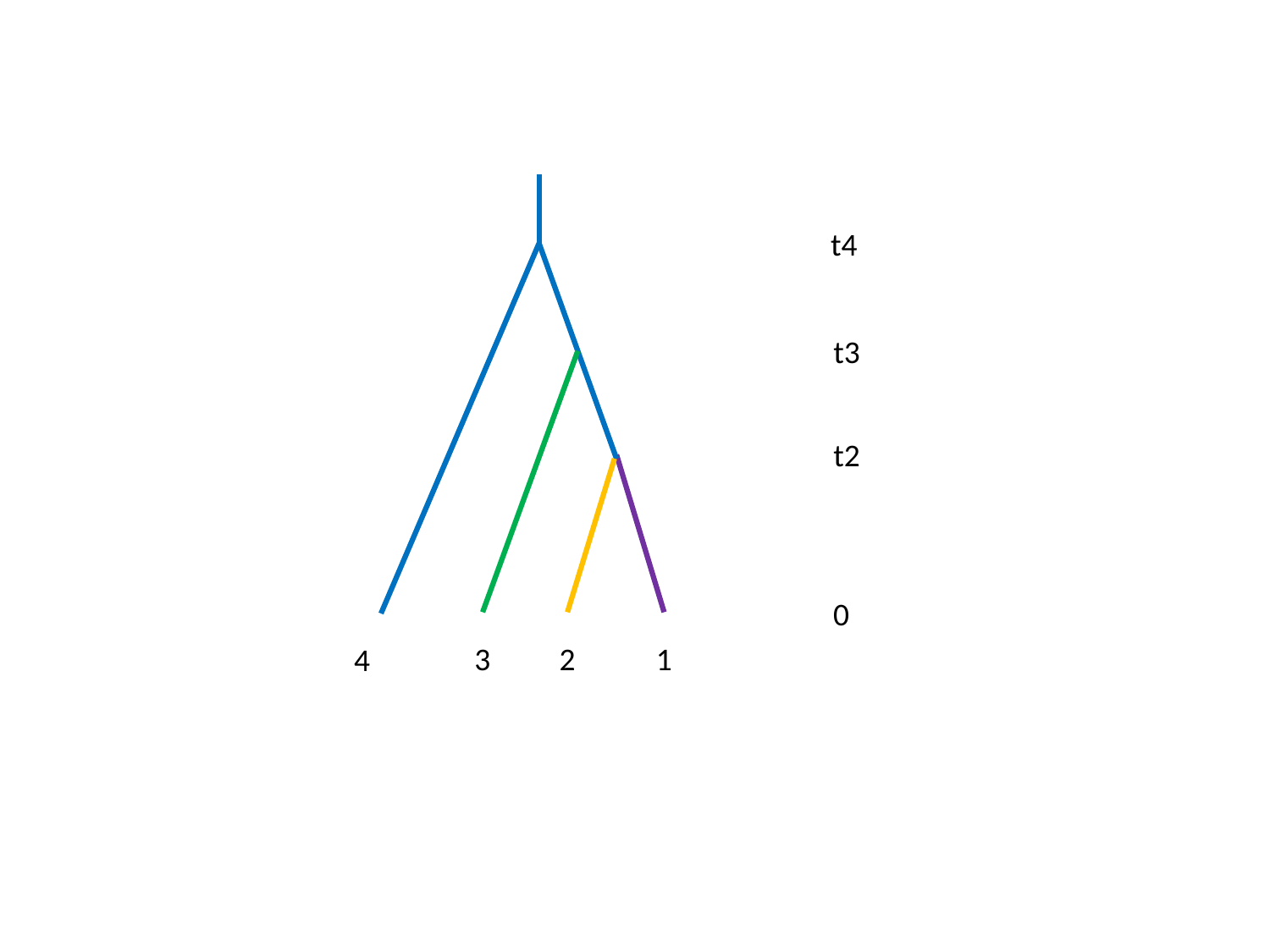

t4
t3
t2
0
3
2
1
4

## Slide 54
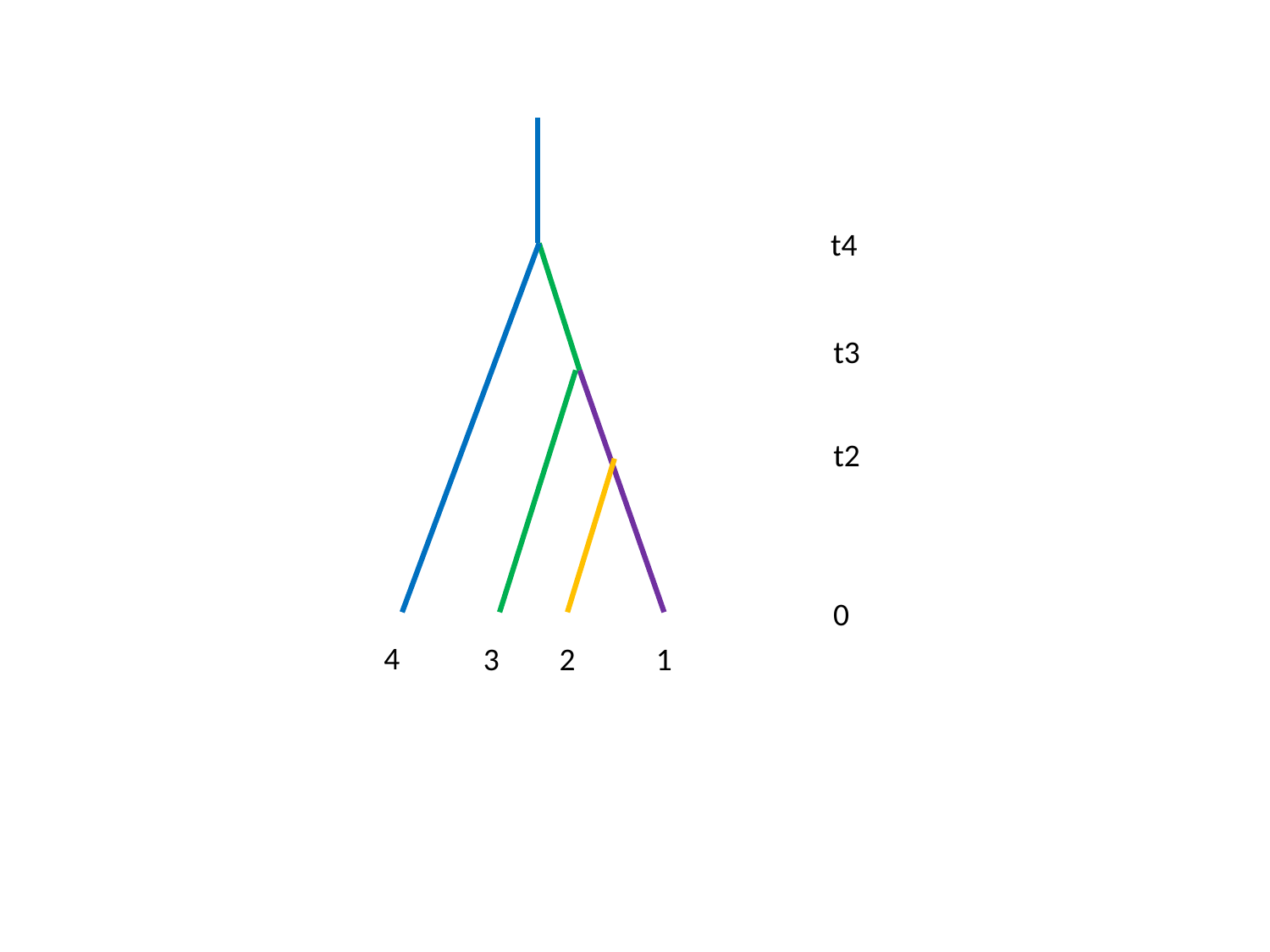

t4
t3
t2
0
4
3
2
1

## Slide 55
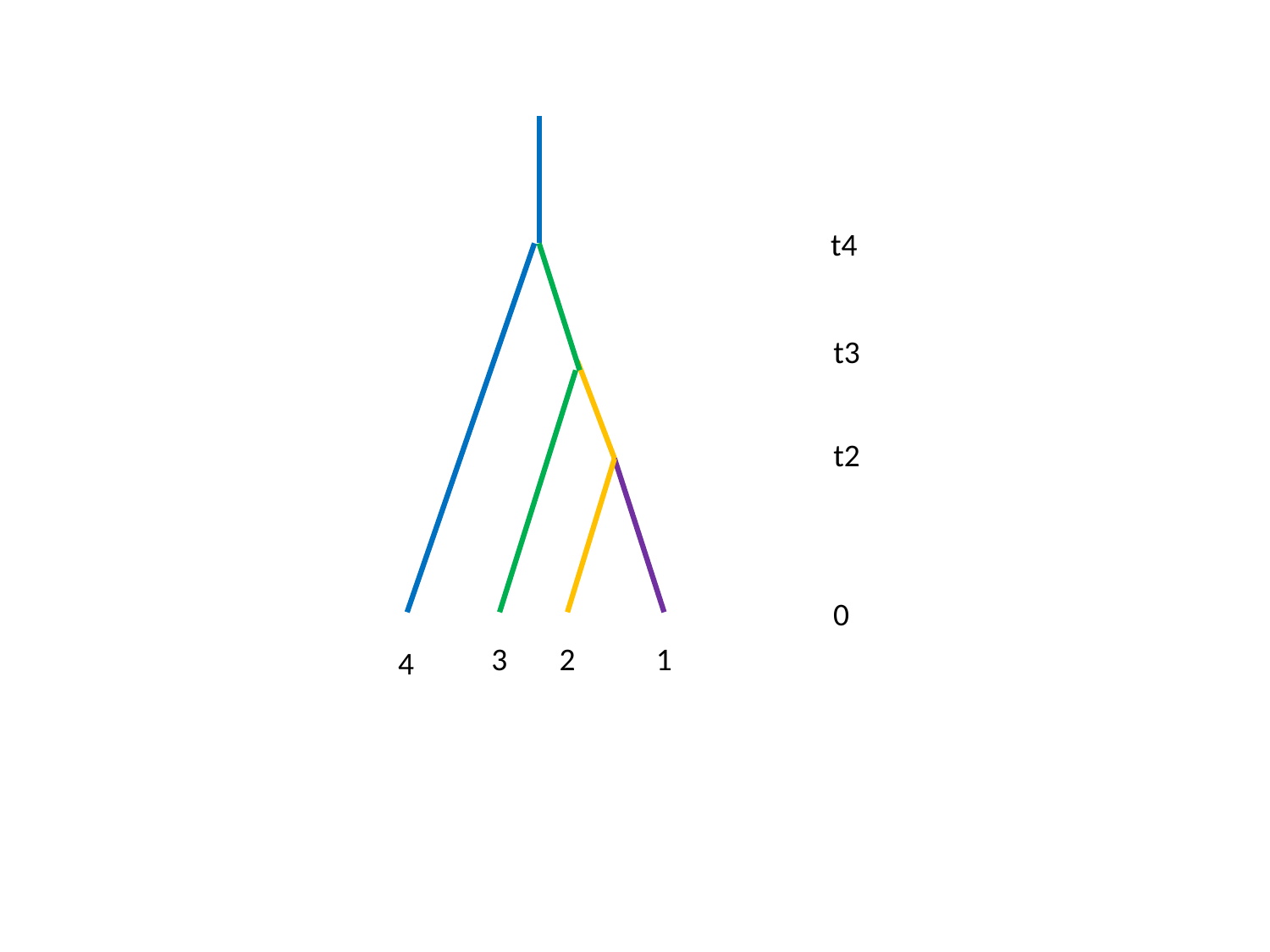

t4
t3
t2
0
3
2
1
4

## Slide 56
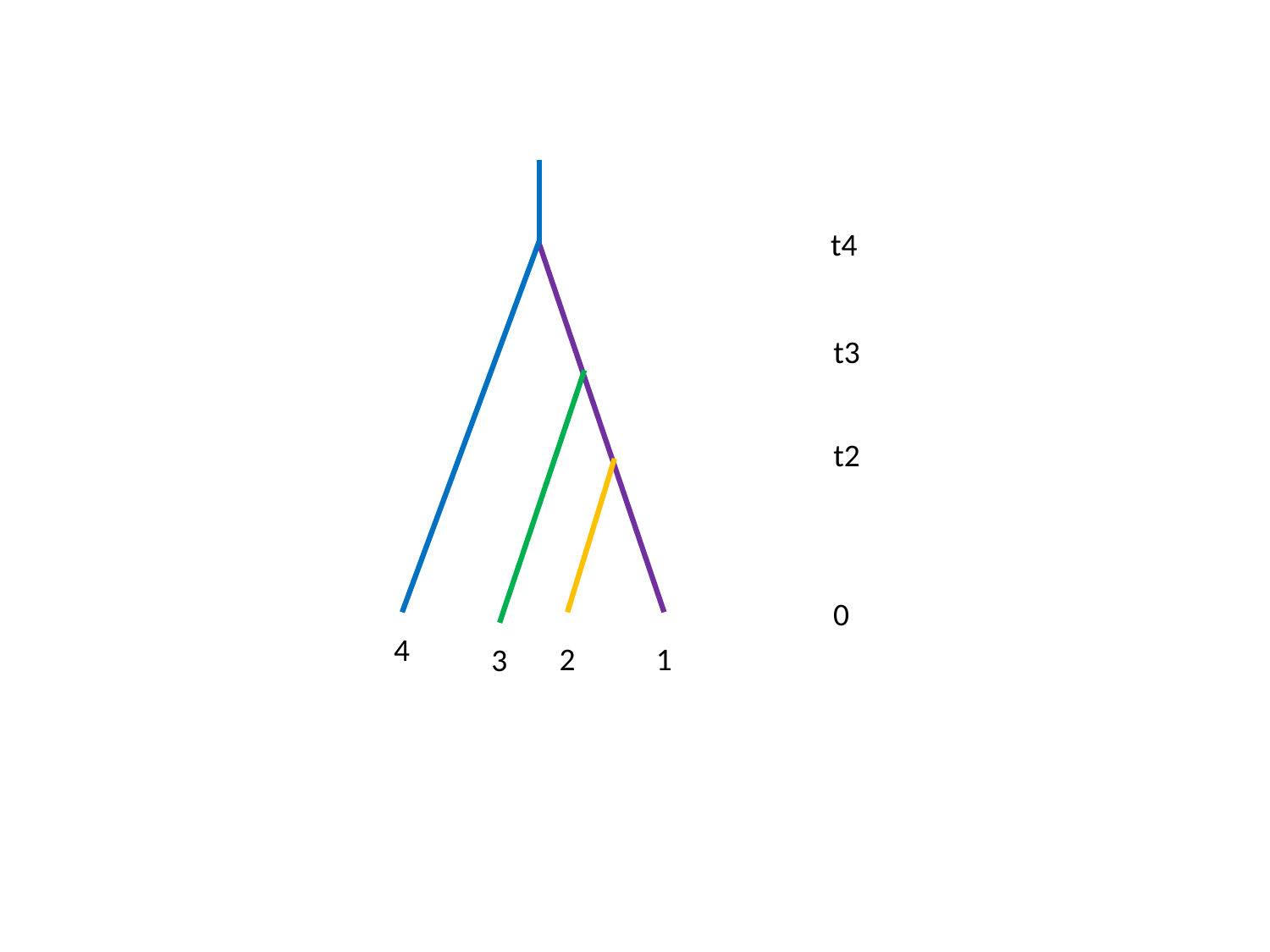

t4
t3
t2
0
4
2
1
3

## Slide 57
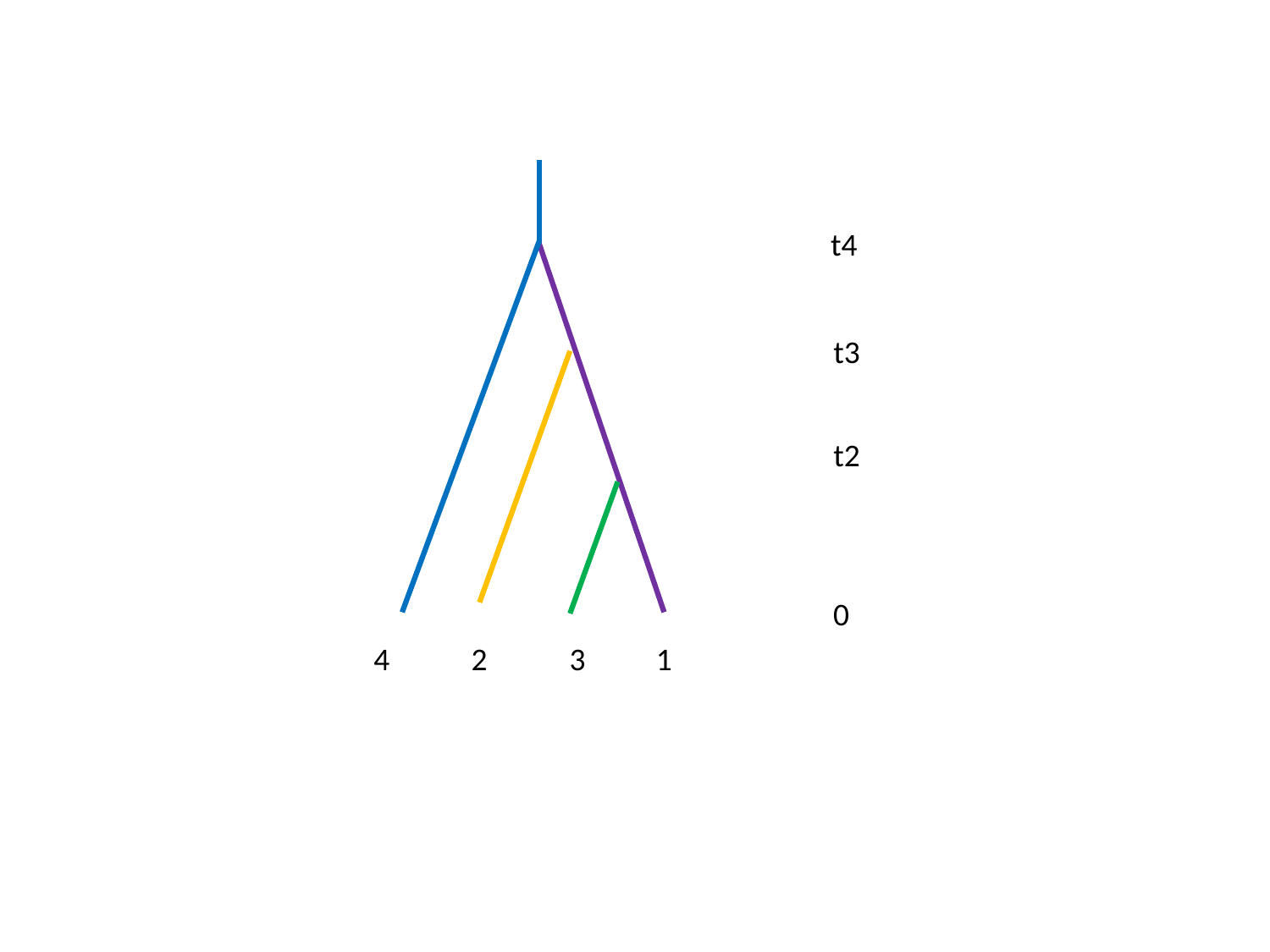

t4
t3
t2
0
4
2
3
1

## Slide 58
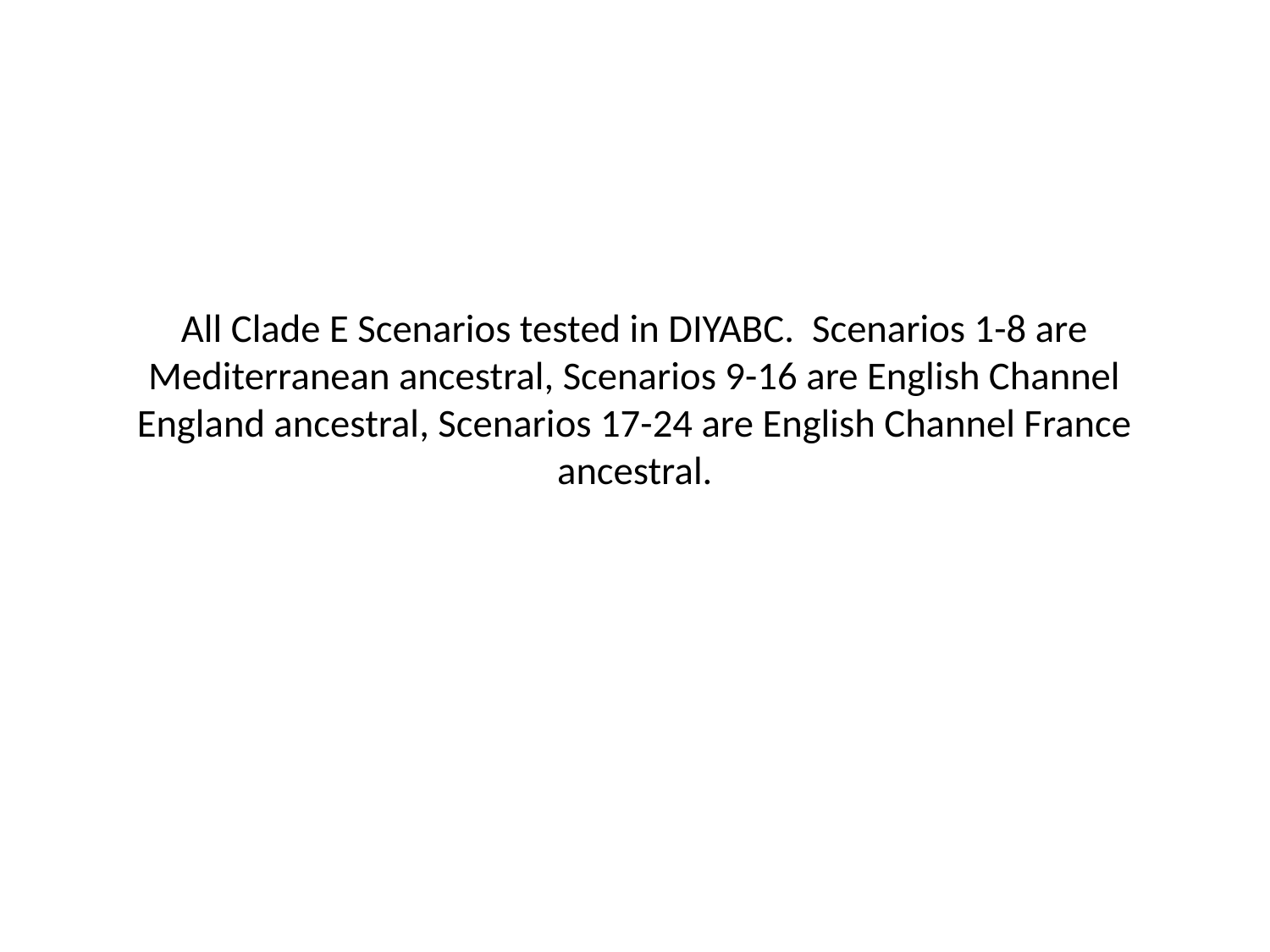

# All Clade E Scenarios tested in DIYABC. Scenarios 1-8 are Mediterranean ancestral, Scenarios 9-16 are English Channel England ancestral, Scenarios 17-24 are English Channel France ancestral.

## Slide 59
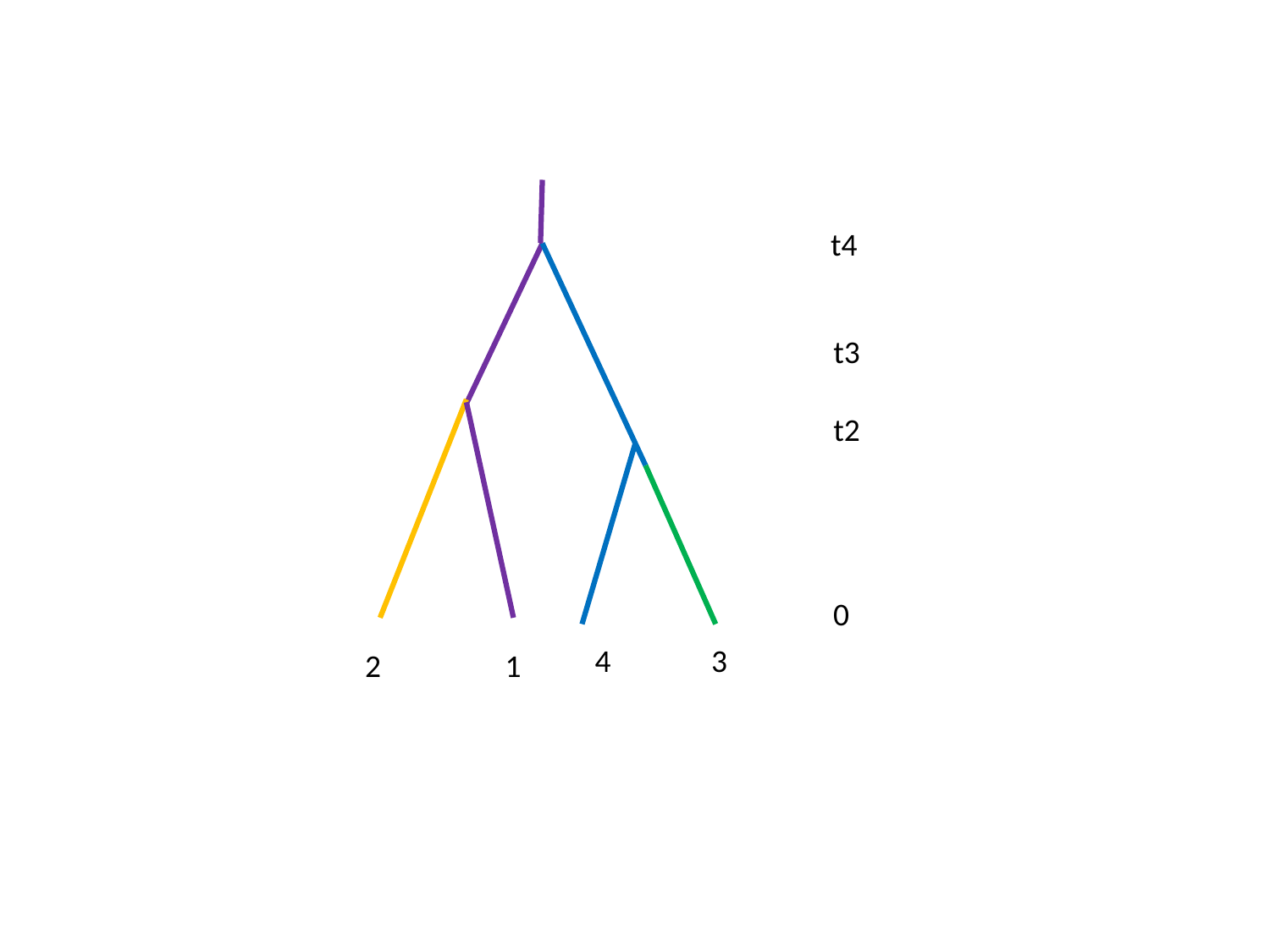

t4
t3
t2
0
4
3
2
1

## Slide 60
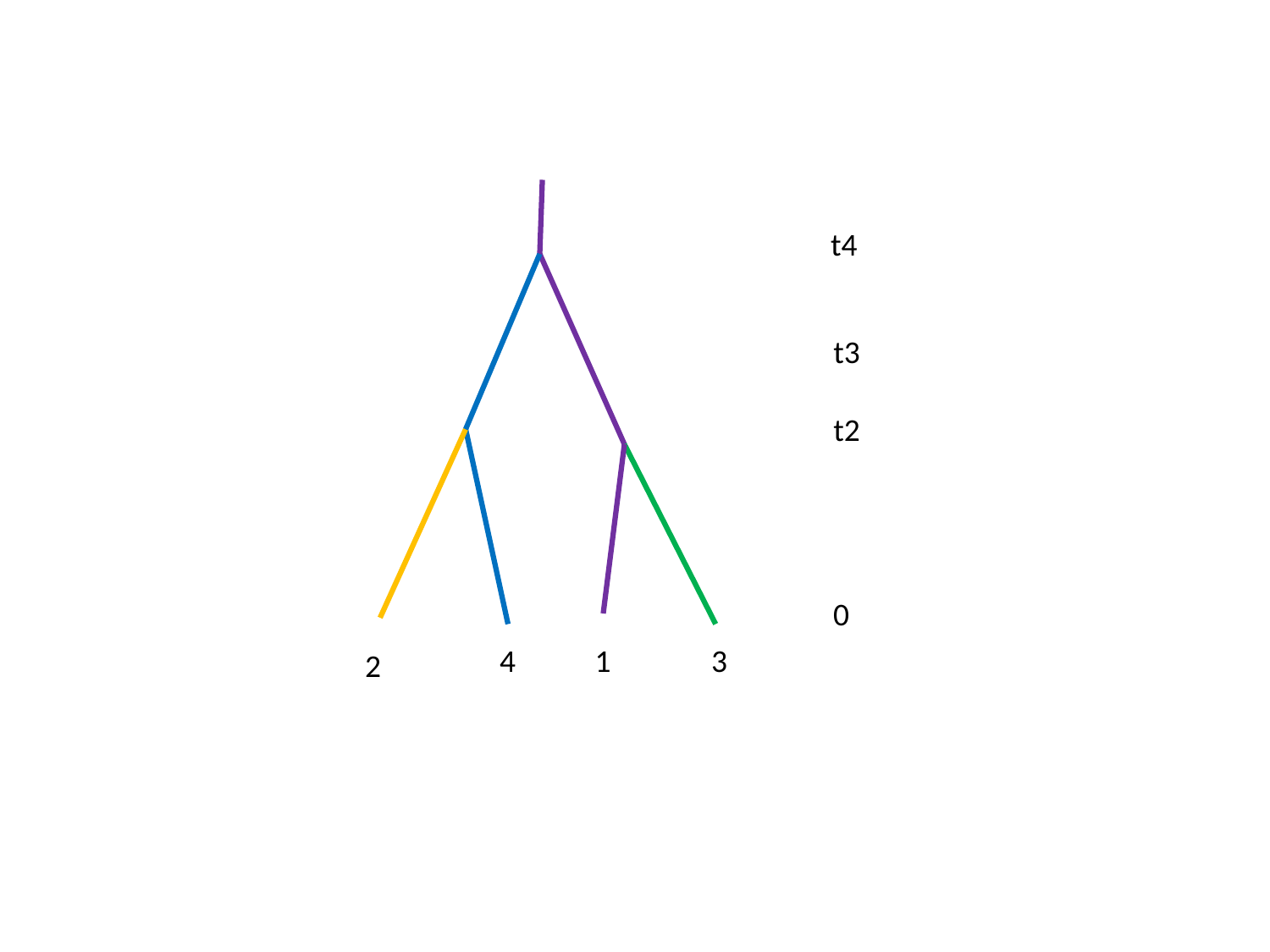

t4
t3
t2
0
4
1
3
2

## Slide 61
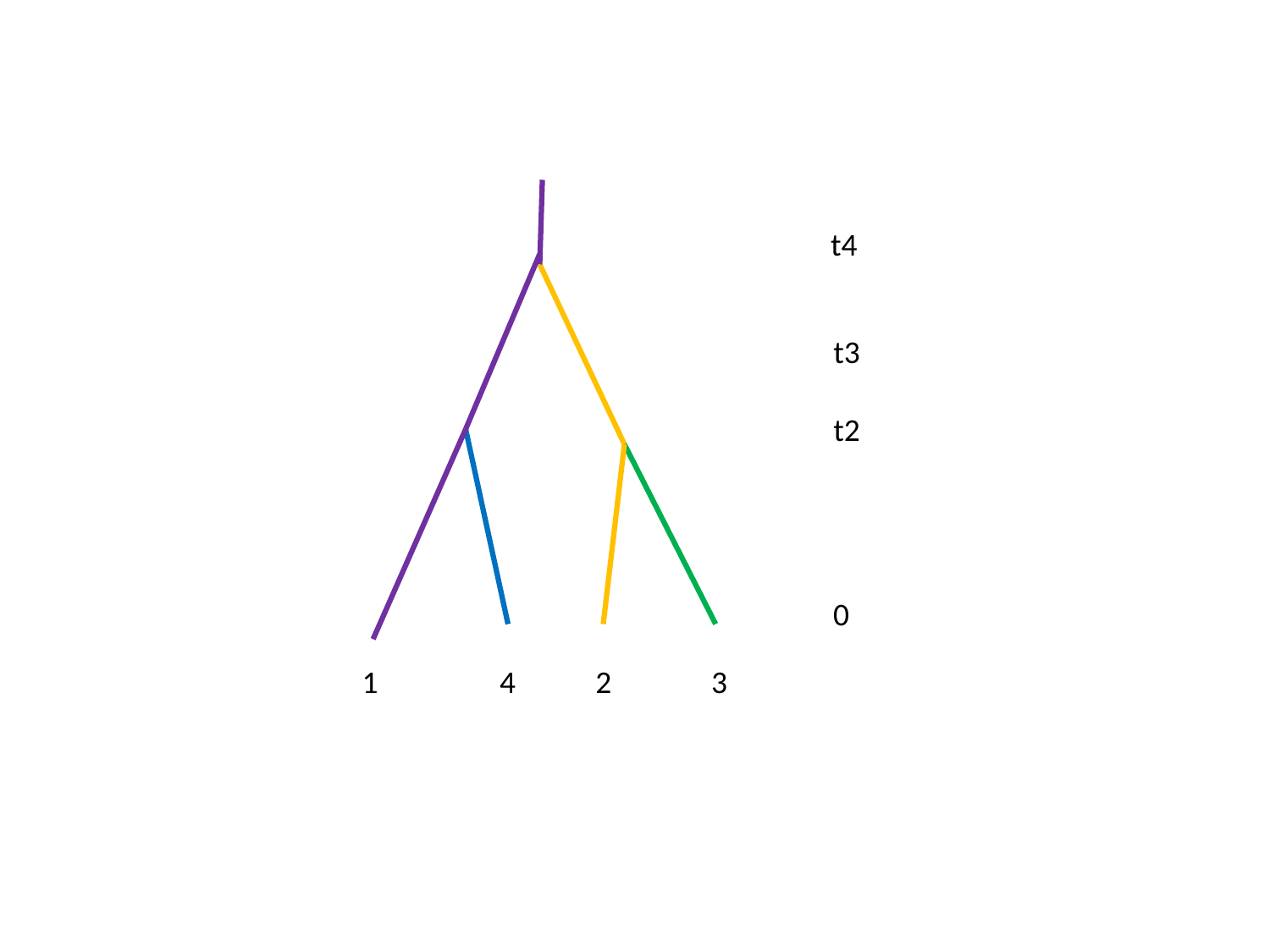

t4
t3
t2
0
1
4
2
3

## Slide 62
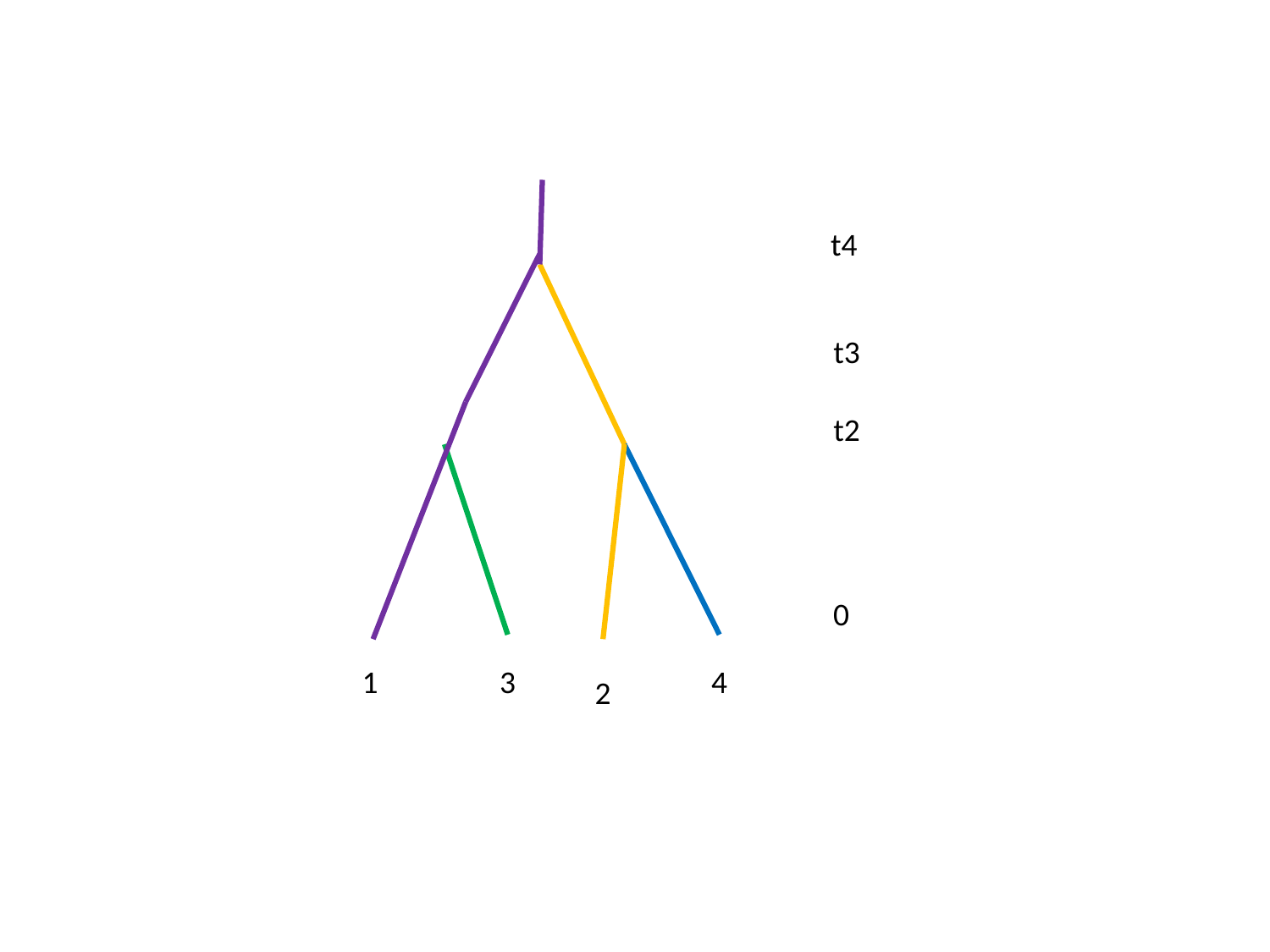

t4
t3
t2
0
1
3
4
2

## Slide 63
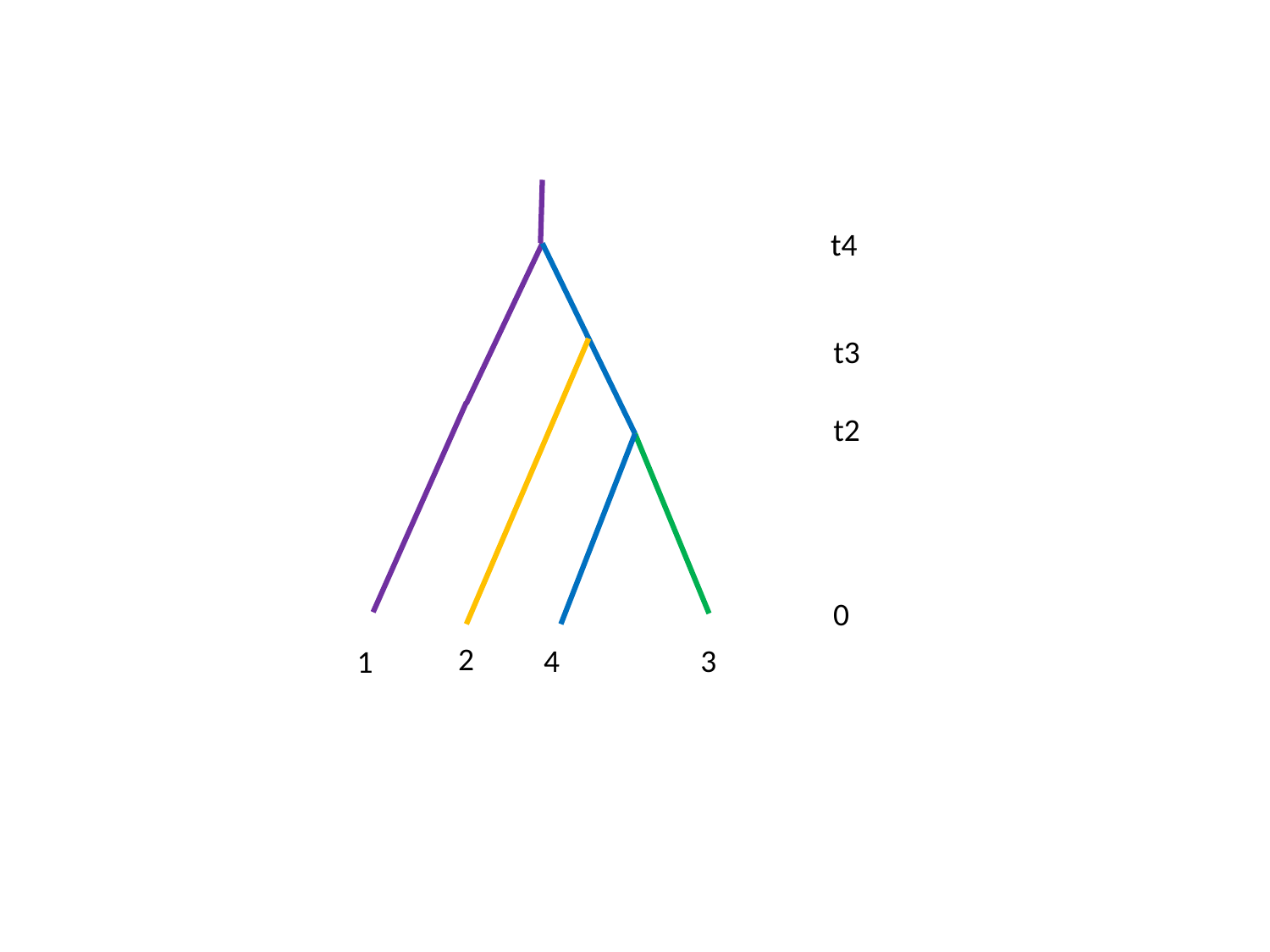

t4
t3
t2
0
2
4
3
1

## Slide 64
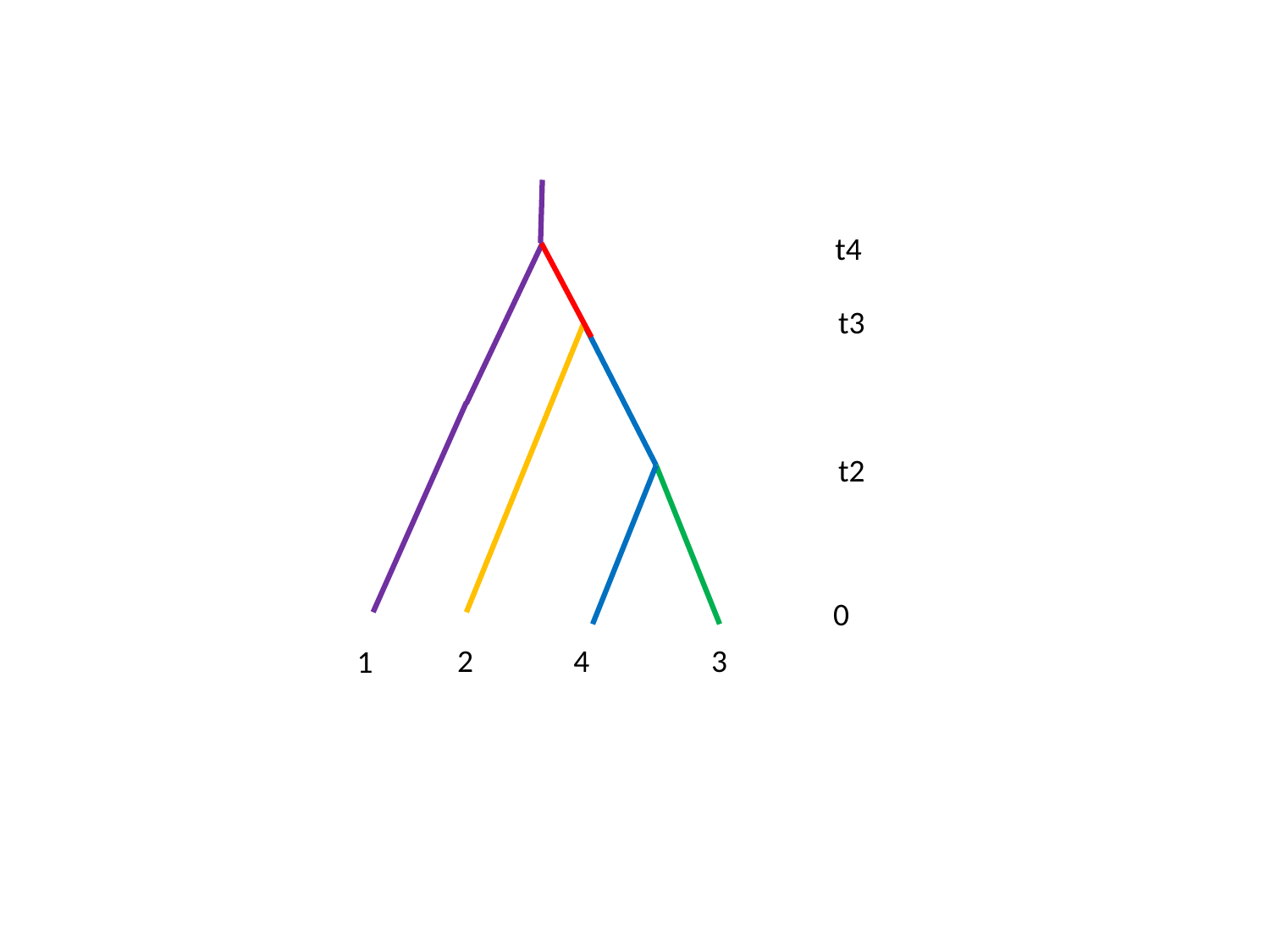

t4
t3
t2
0
2
4
3
1

## Slide 65
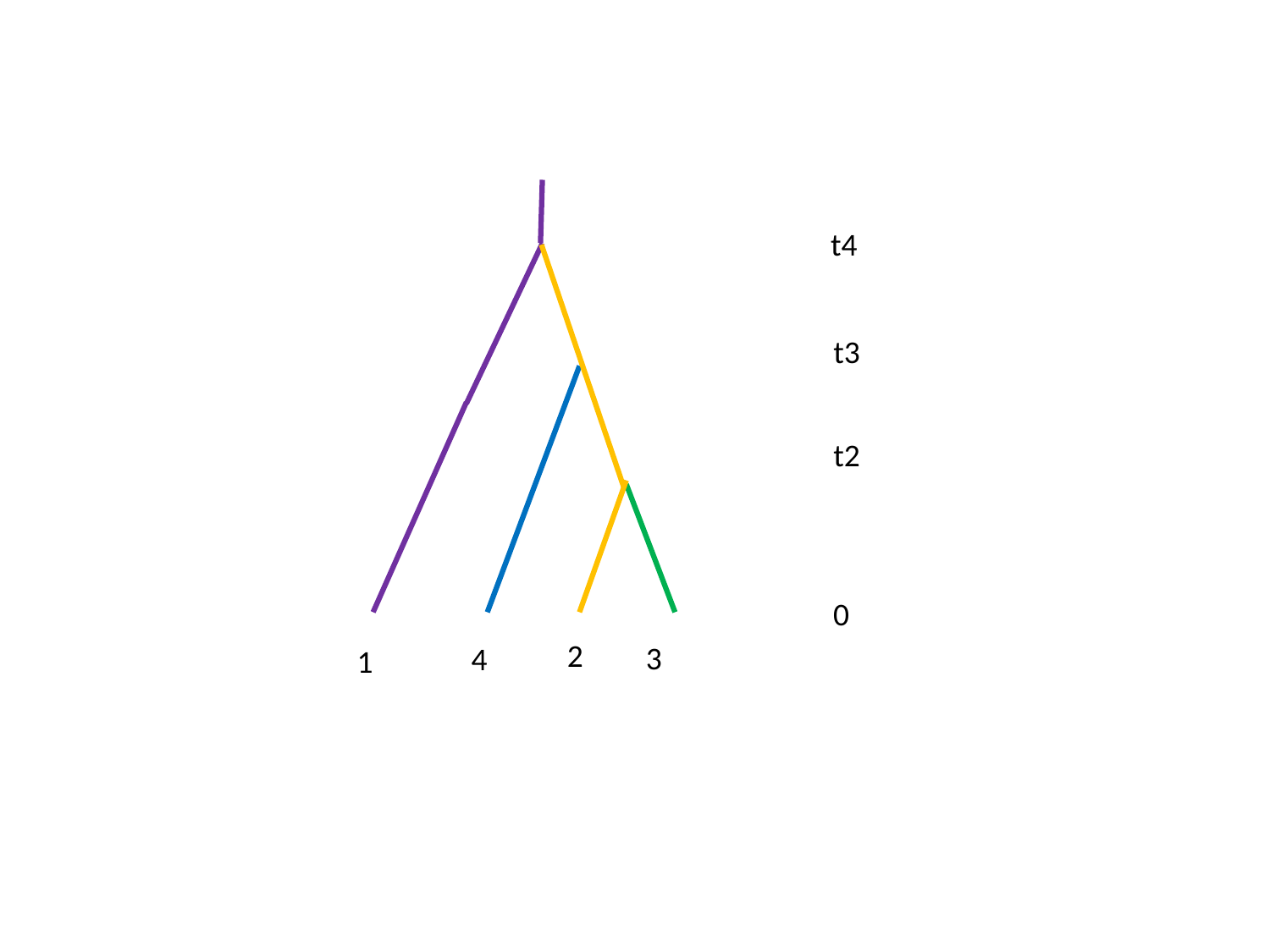

t4
t3
t2
0
2
3
4
1

## Slide 66
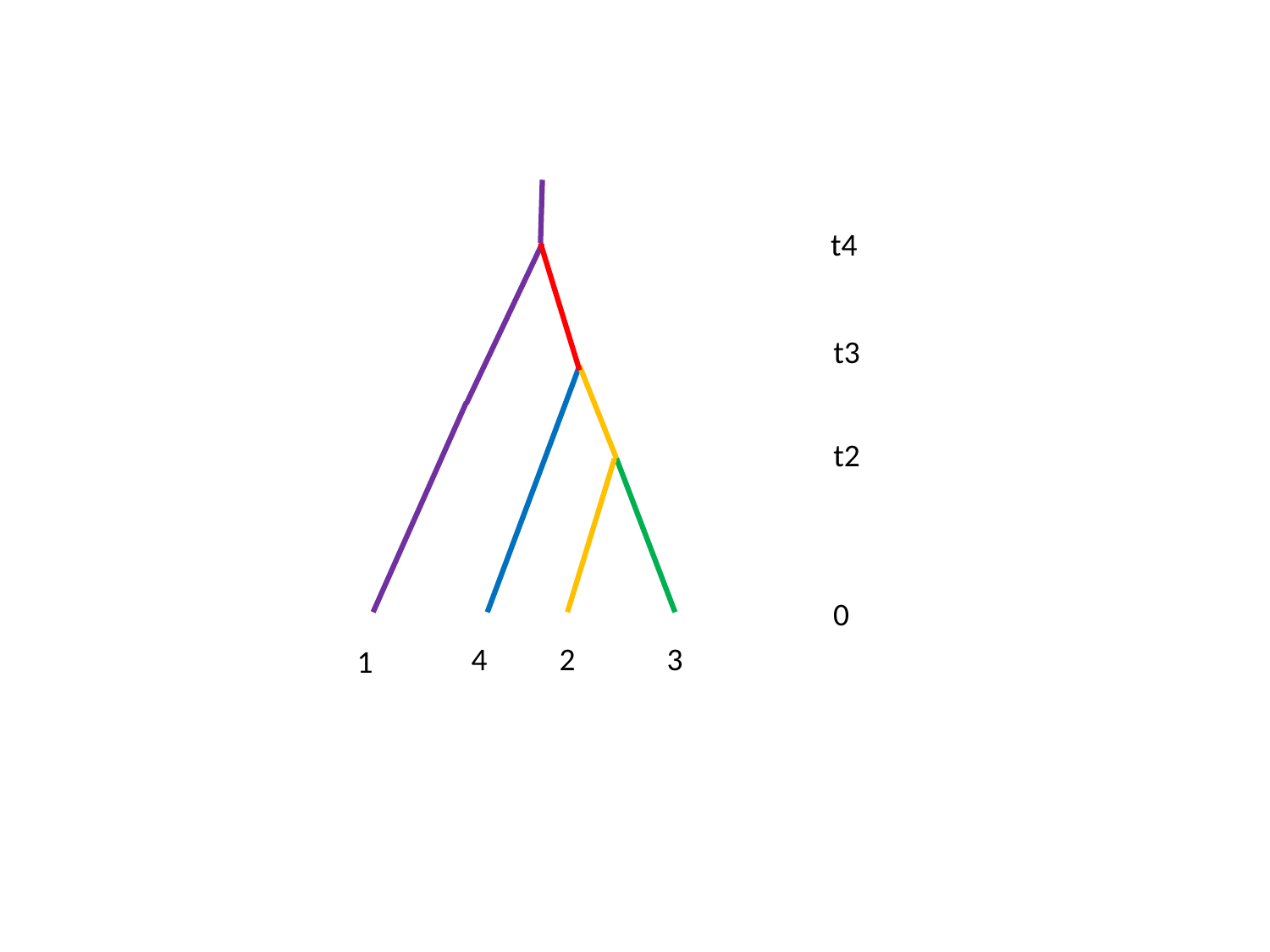

t4
t3
t2
0
4
2
3
1

## Slide 67
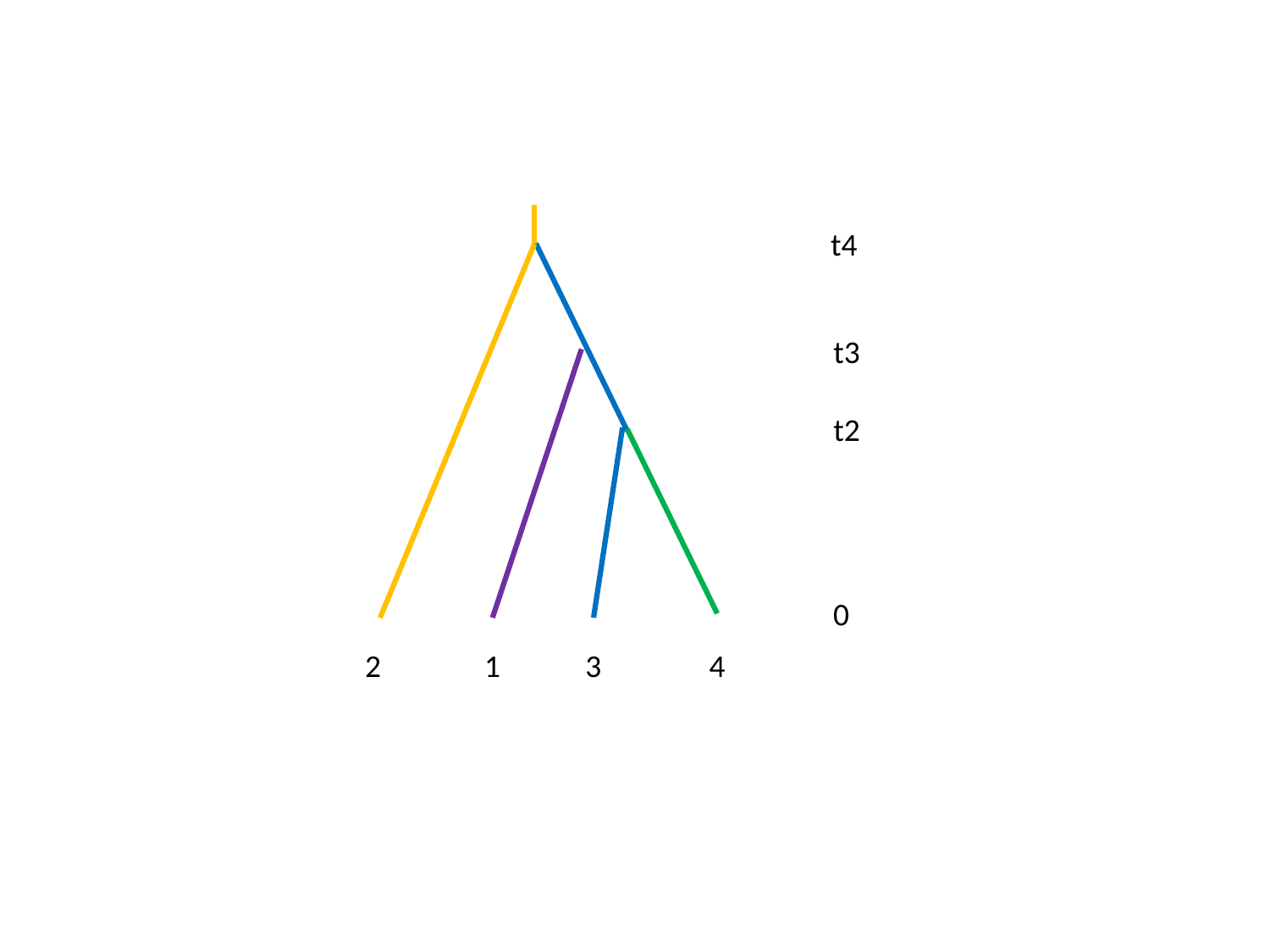

t4
t3
t2
0
2
1
3
4

## Slide 68
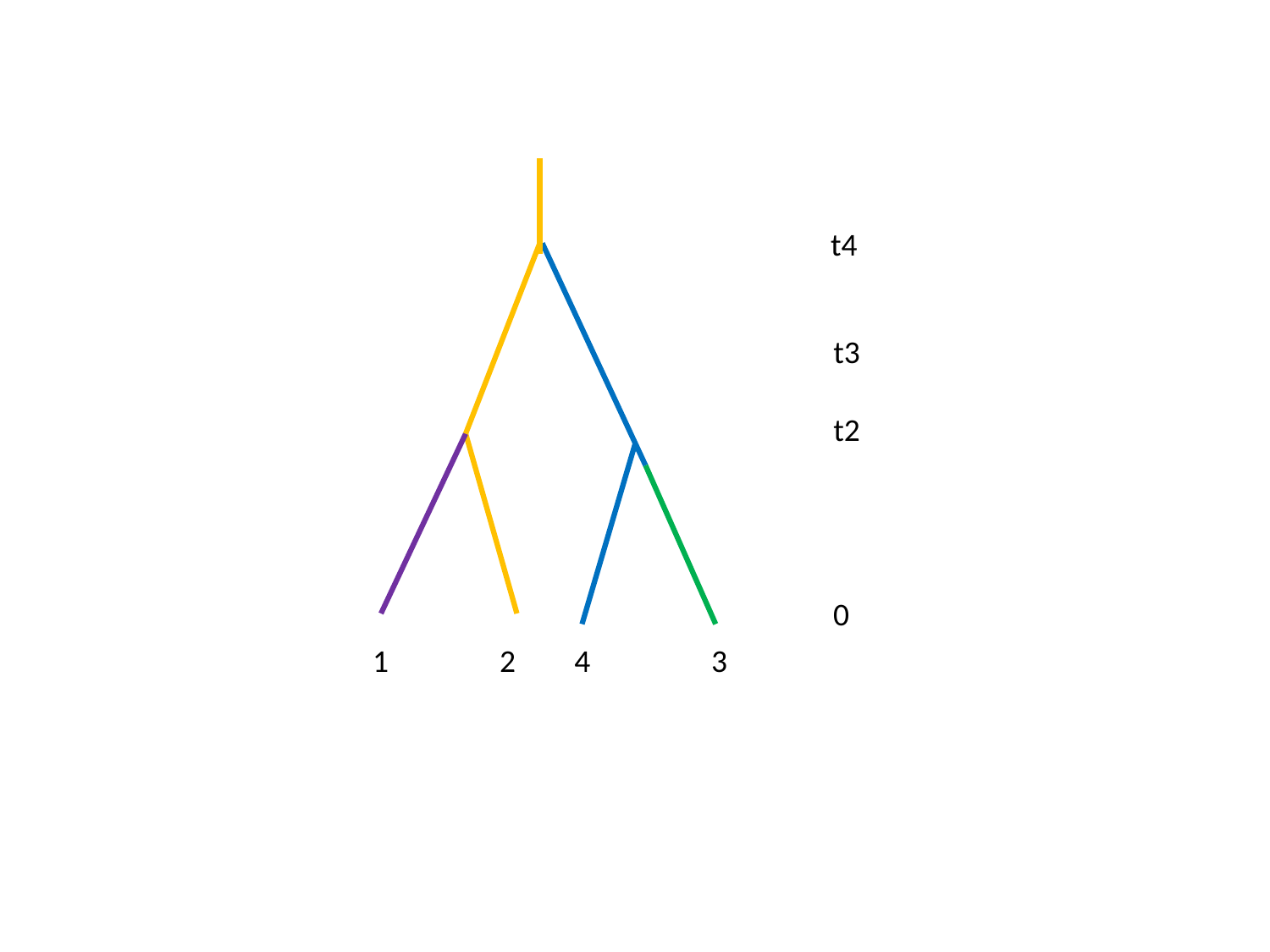

t4
t3
t2
0
1
2
4
3

## Slide 69
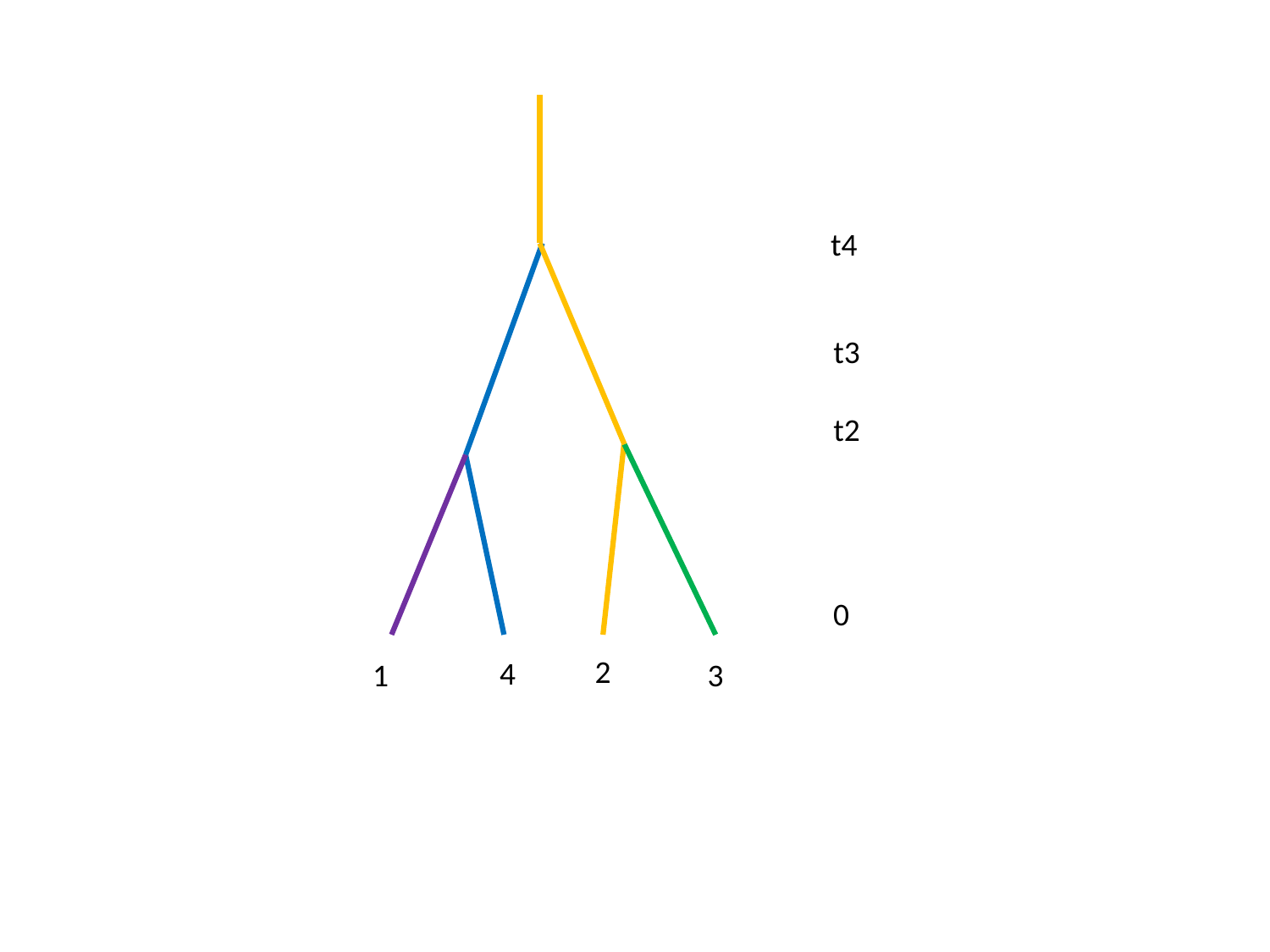

t4
t3
t2
0
2
4
1
3

## Slide 70
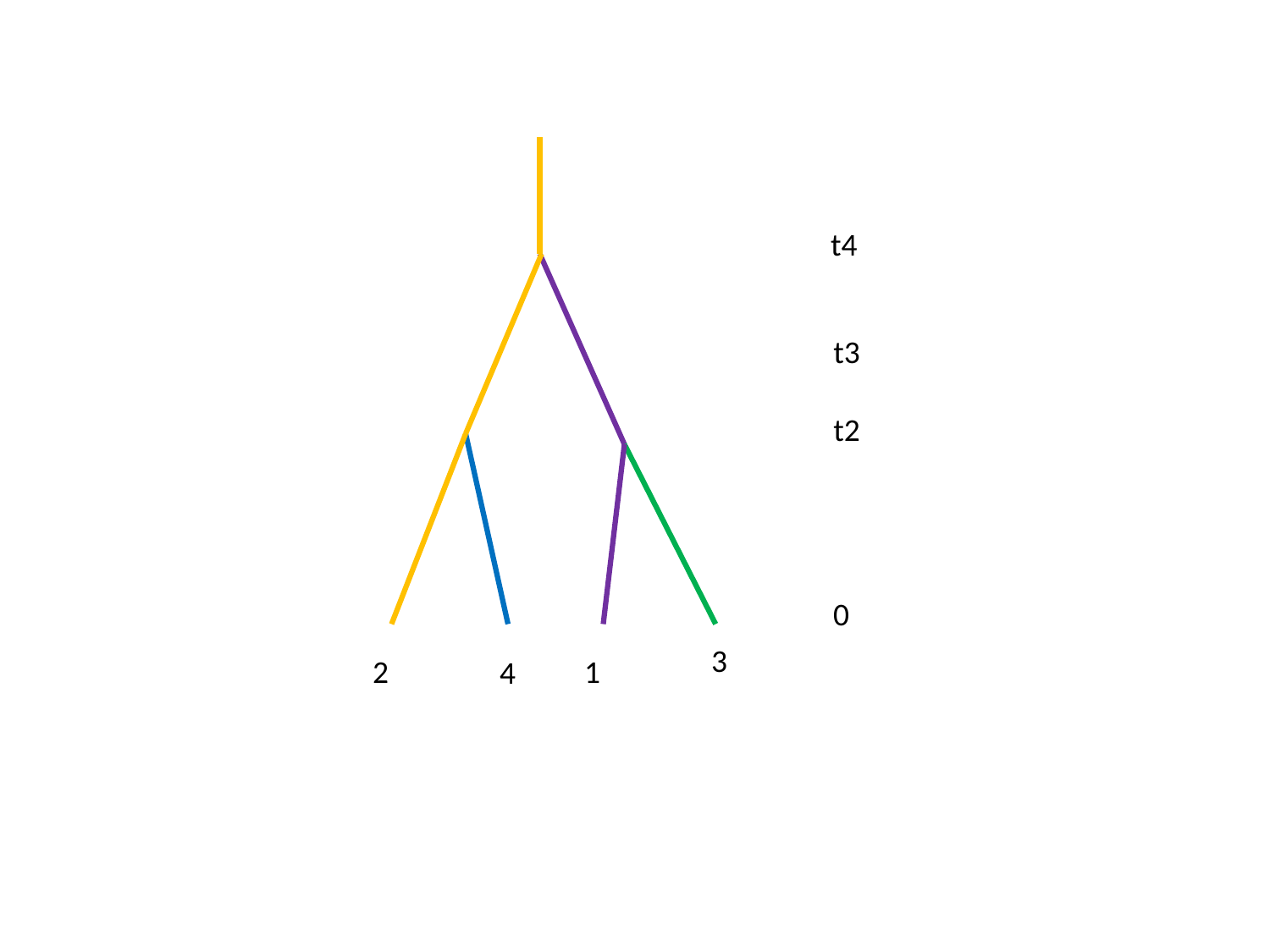

t4
t3
t2
0
3
2
1
4

## Slide 71
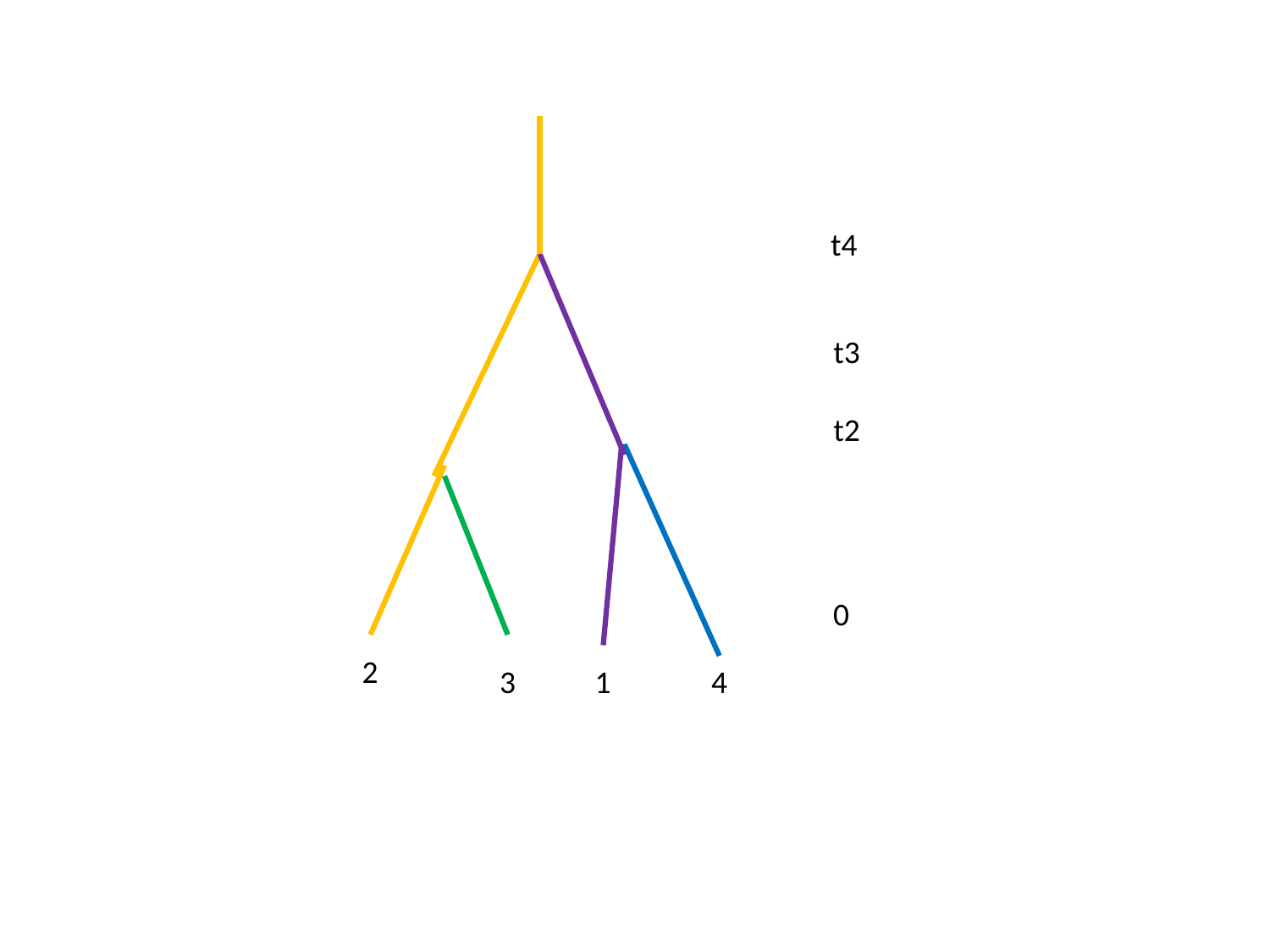

t4
t3
t2
0
2
3
1
4

## Slide 72
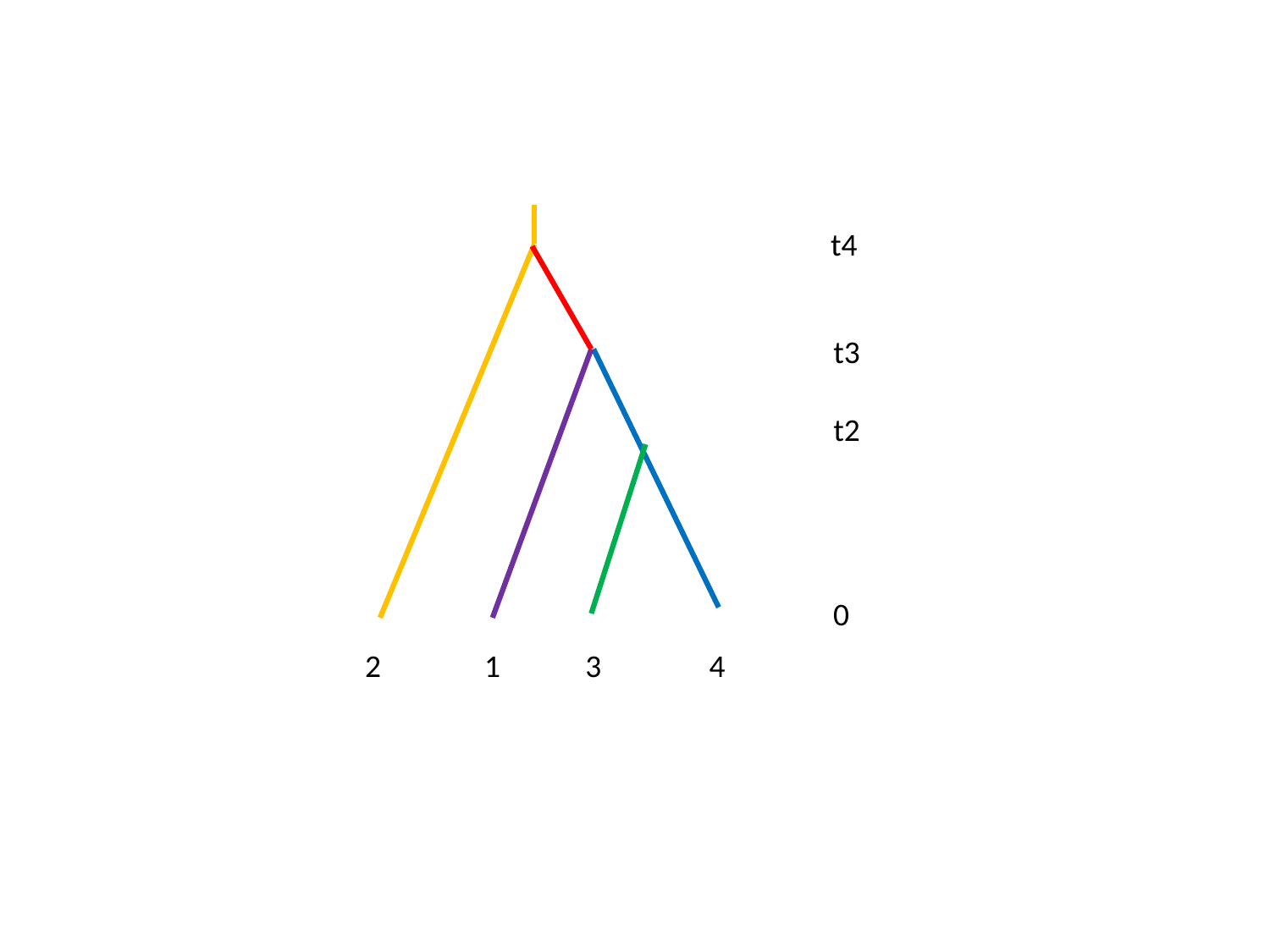

t4
t3
t2
0
2
1
3
4

## Slide 73
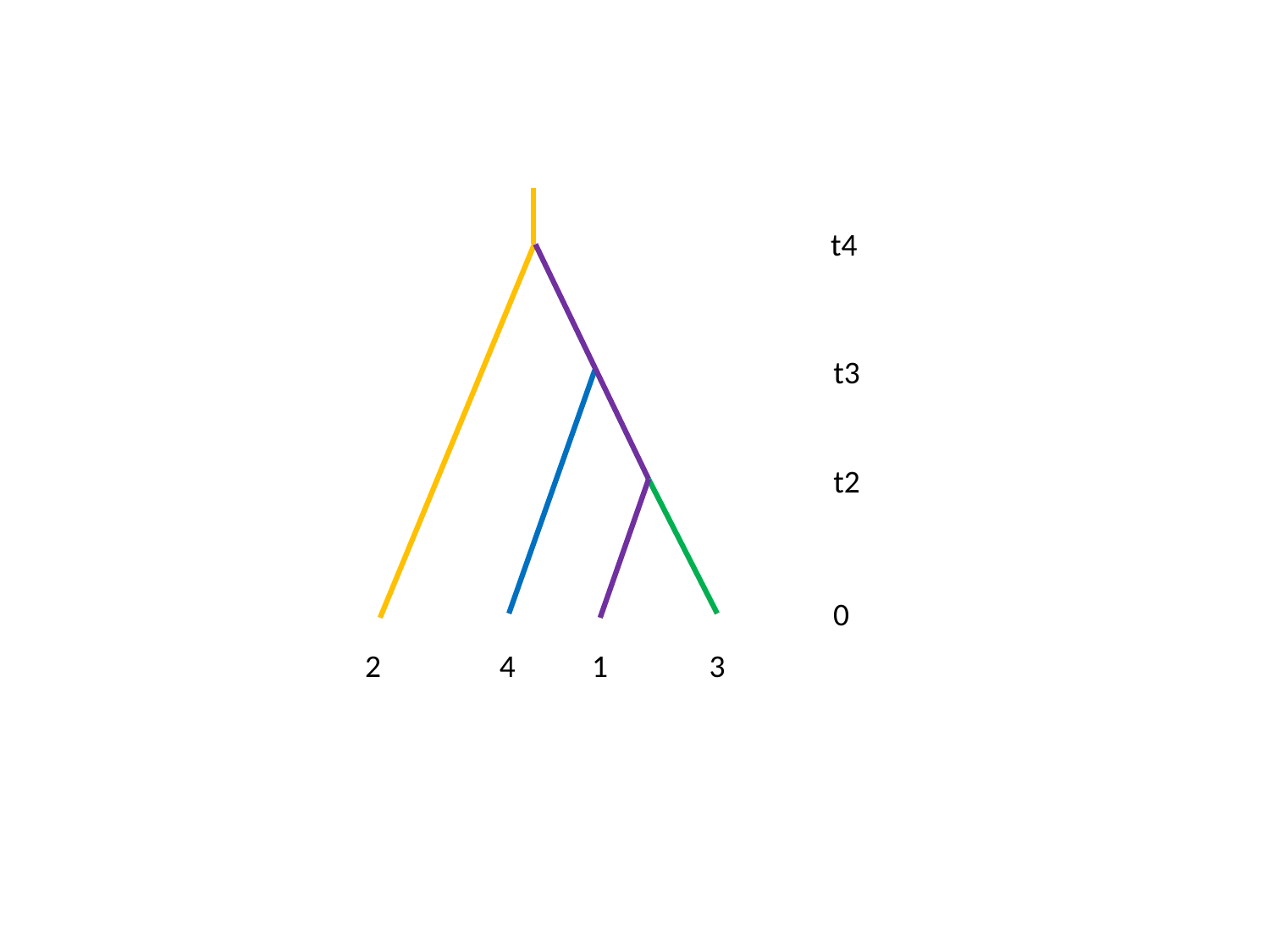

t4
t3
t2
0
2
4
1
3

## Slide 74
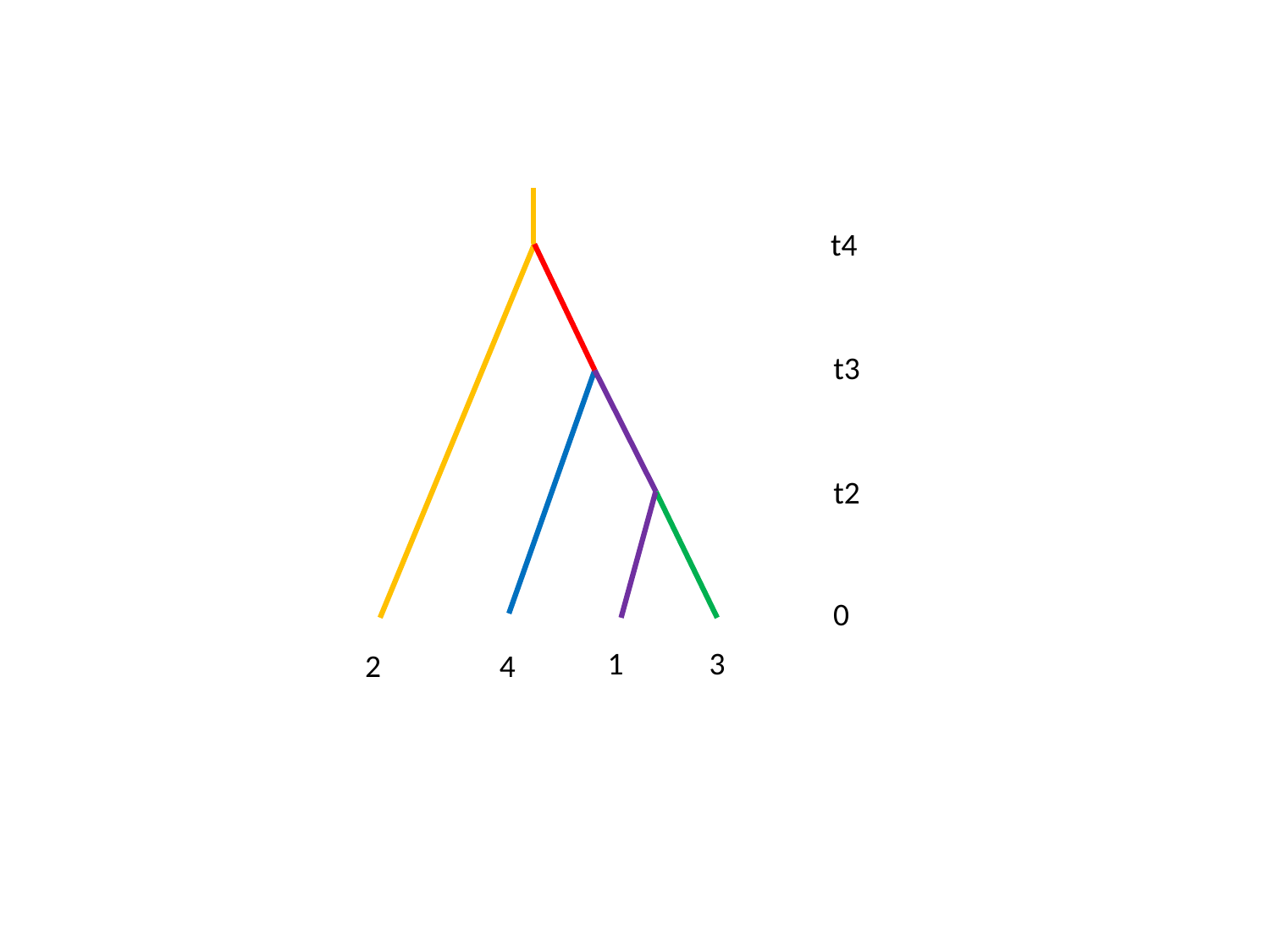

t4
t3
t2
0
1
3
2
4

## Slide 75
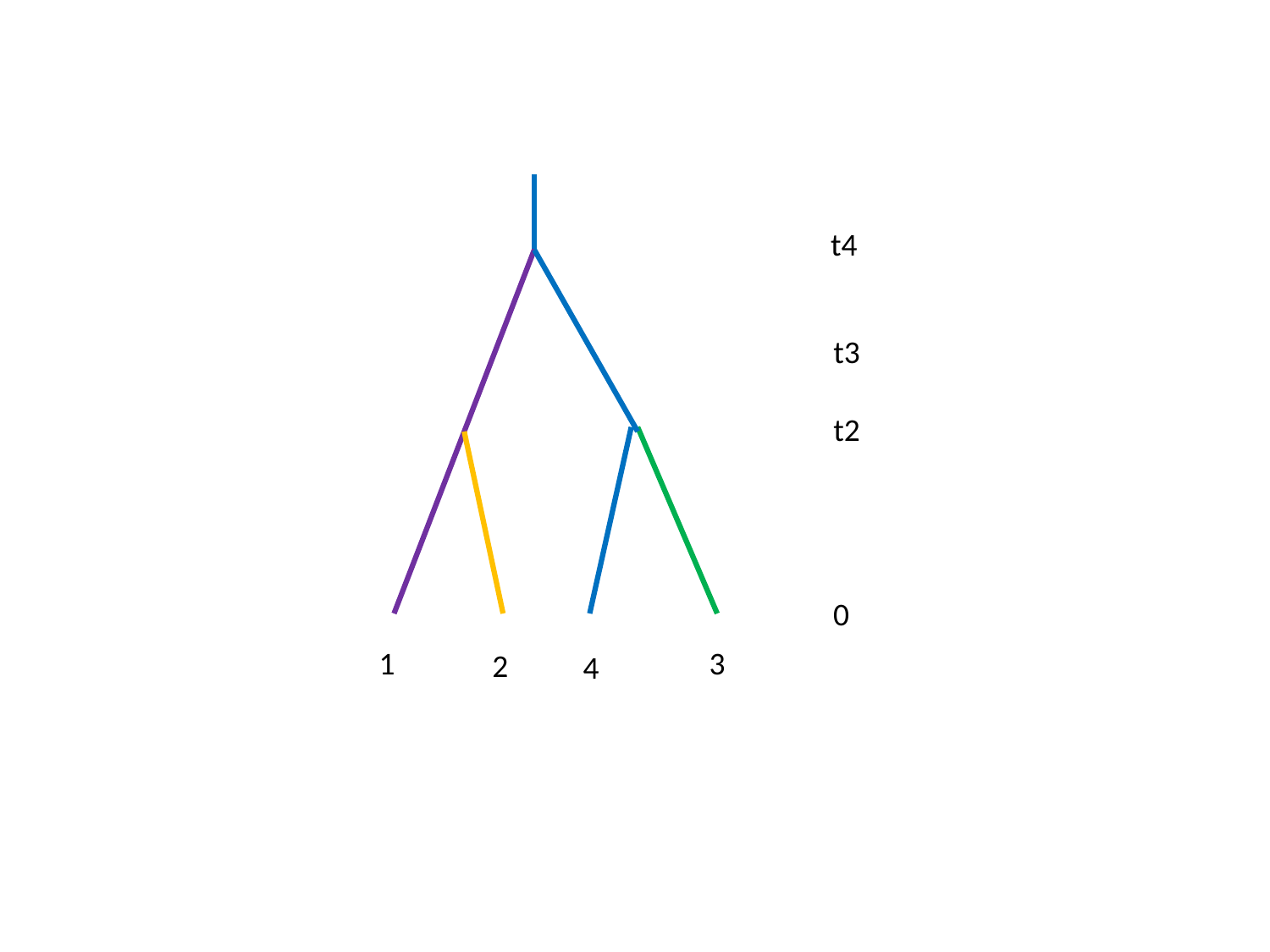

t4
t3
t2
0
1
3
2
4

## Slide 76
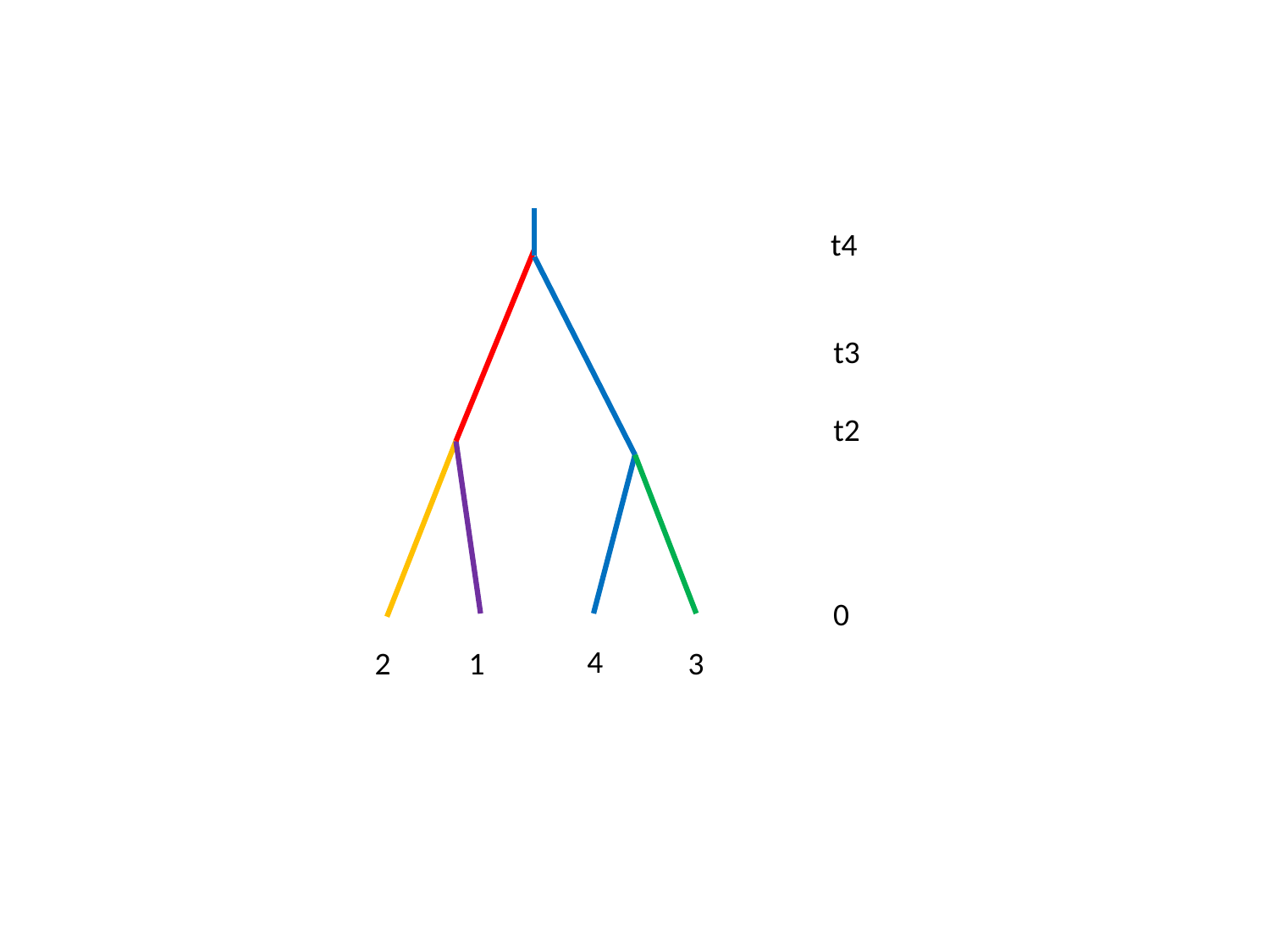

t4
t3
t2
0
4
2
1
3

## Slide 77
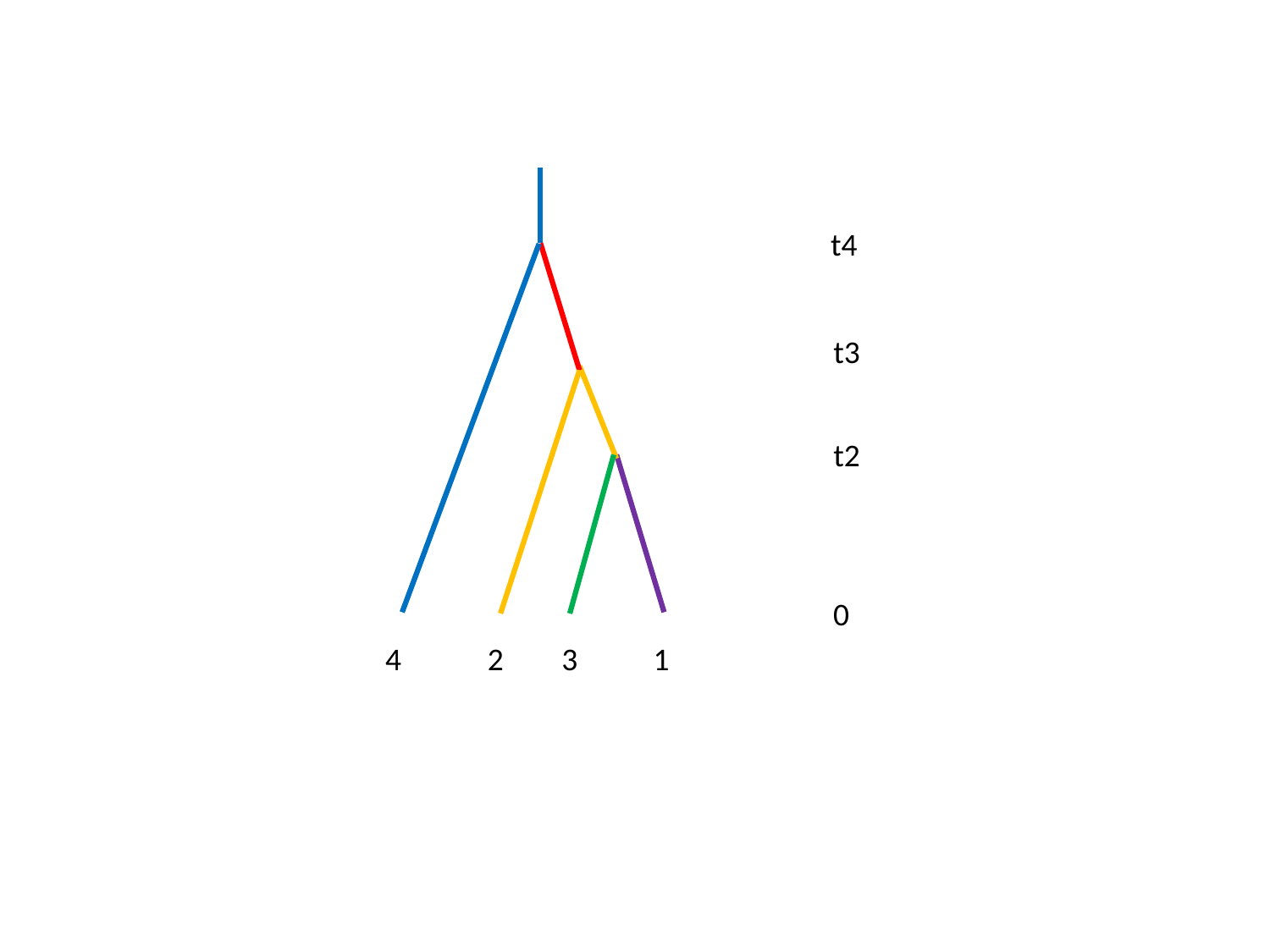

t4
t3
t2
0
4
2
3
1

## Slide 78
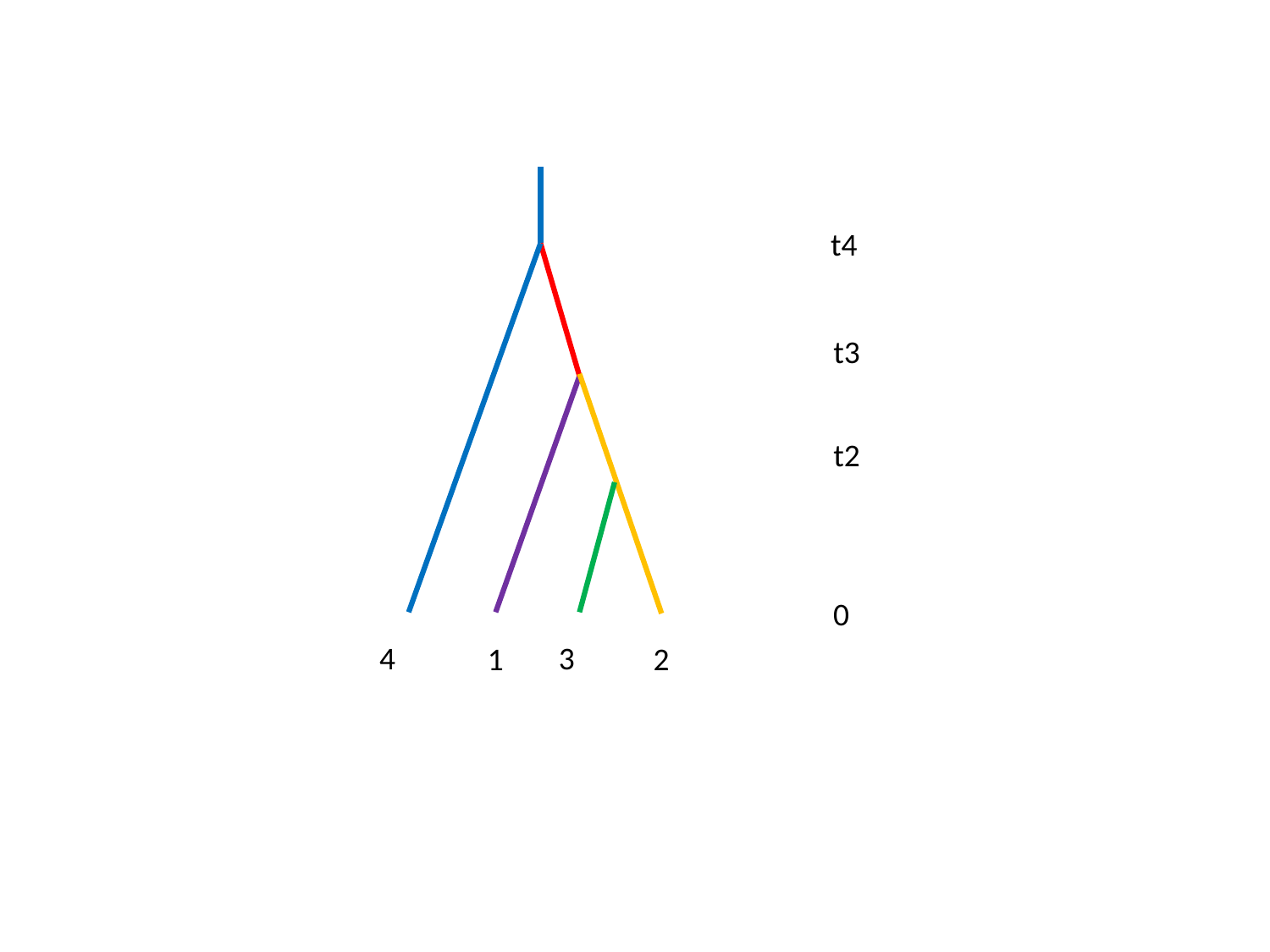

t4
t3
t2
0
4
3
1
2

## Slide 79
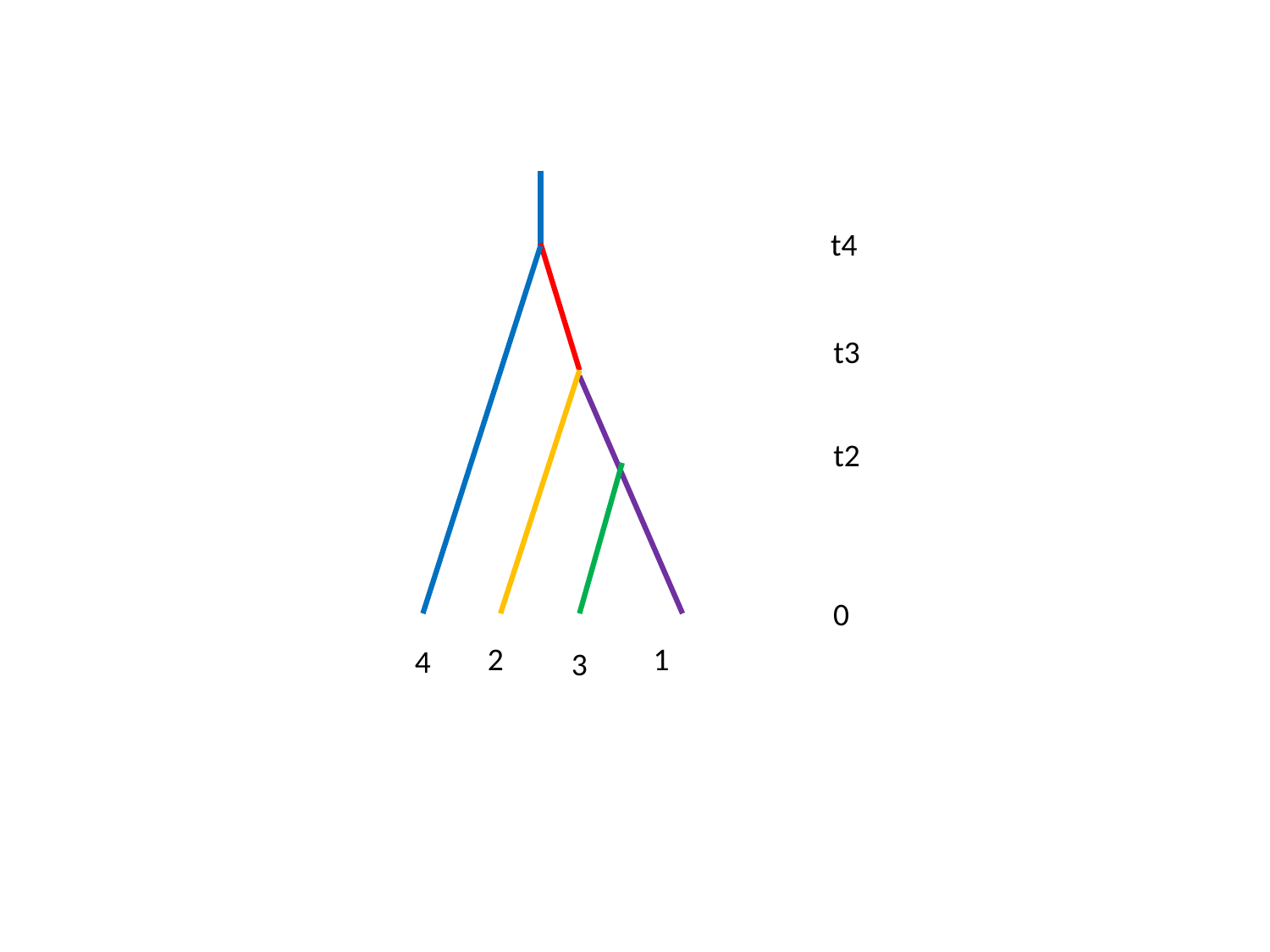

t4
t3
t2
0
2
1
4
3

## Slide 80
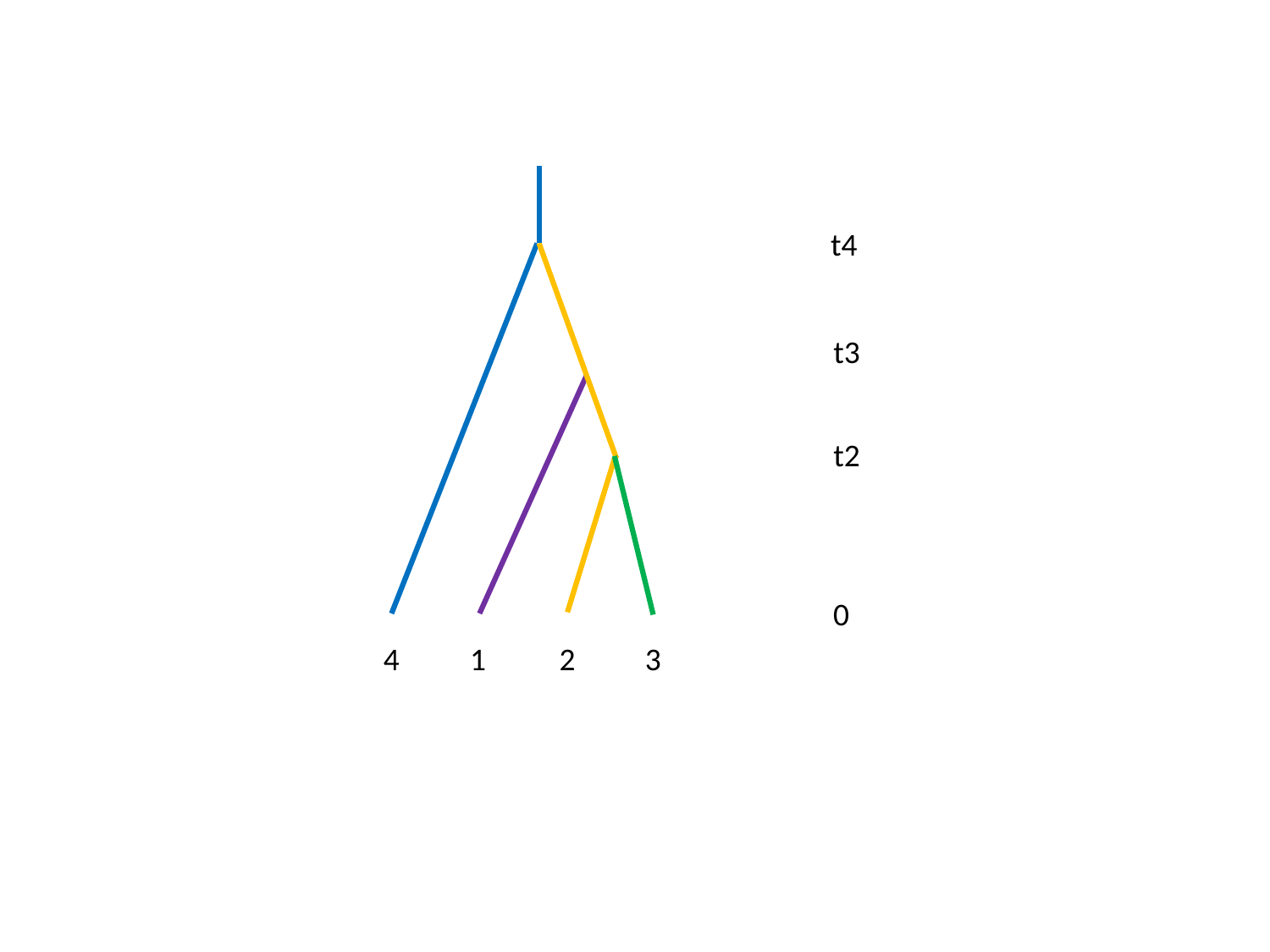

t4
t3
t2
0
4
1
2
3

## Slide 81
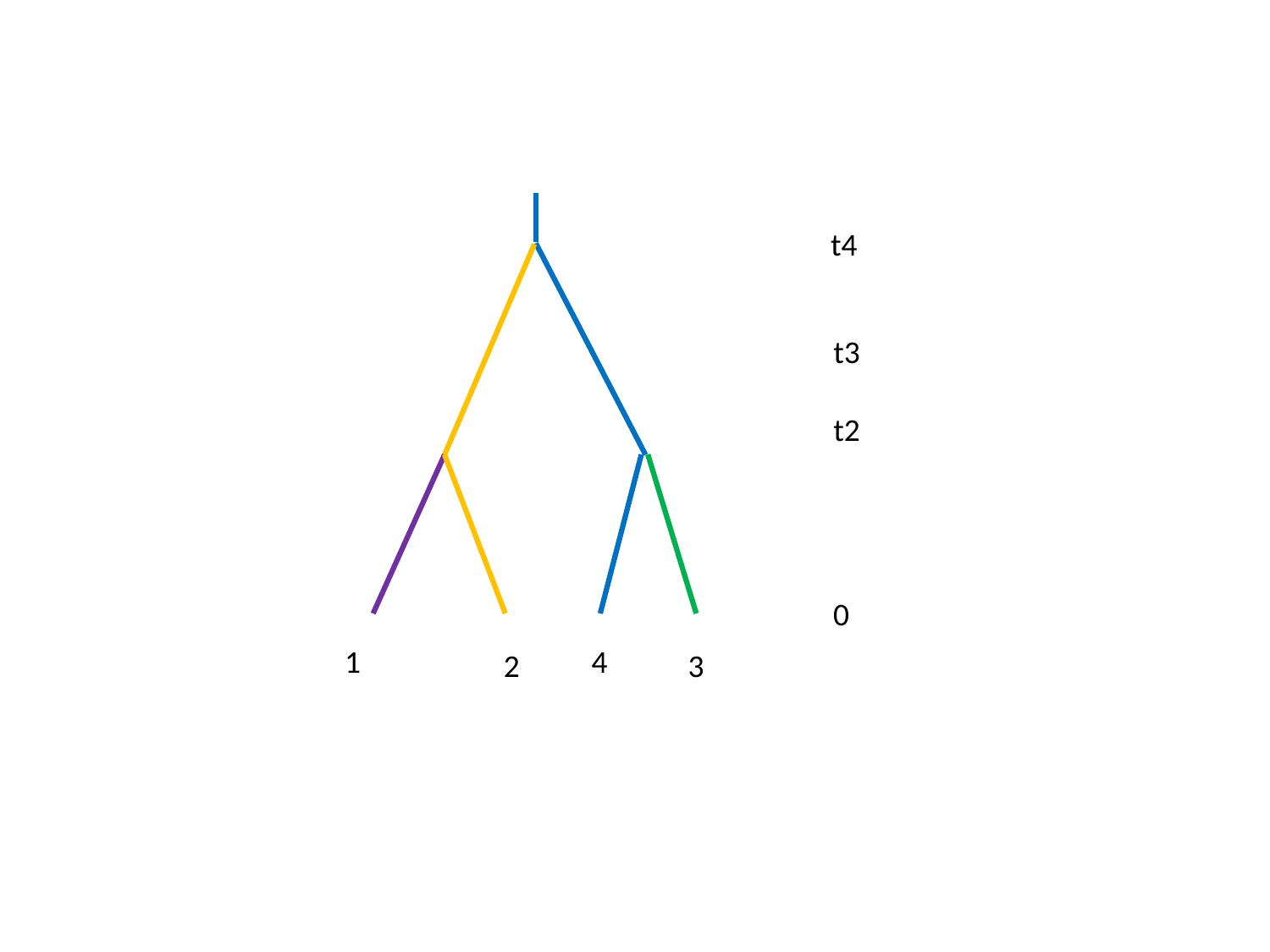

t4
t3
t2
0
1
4
2
3

## Slide 82
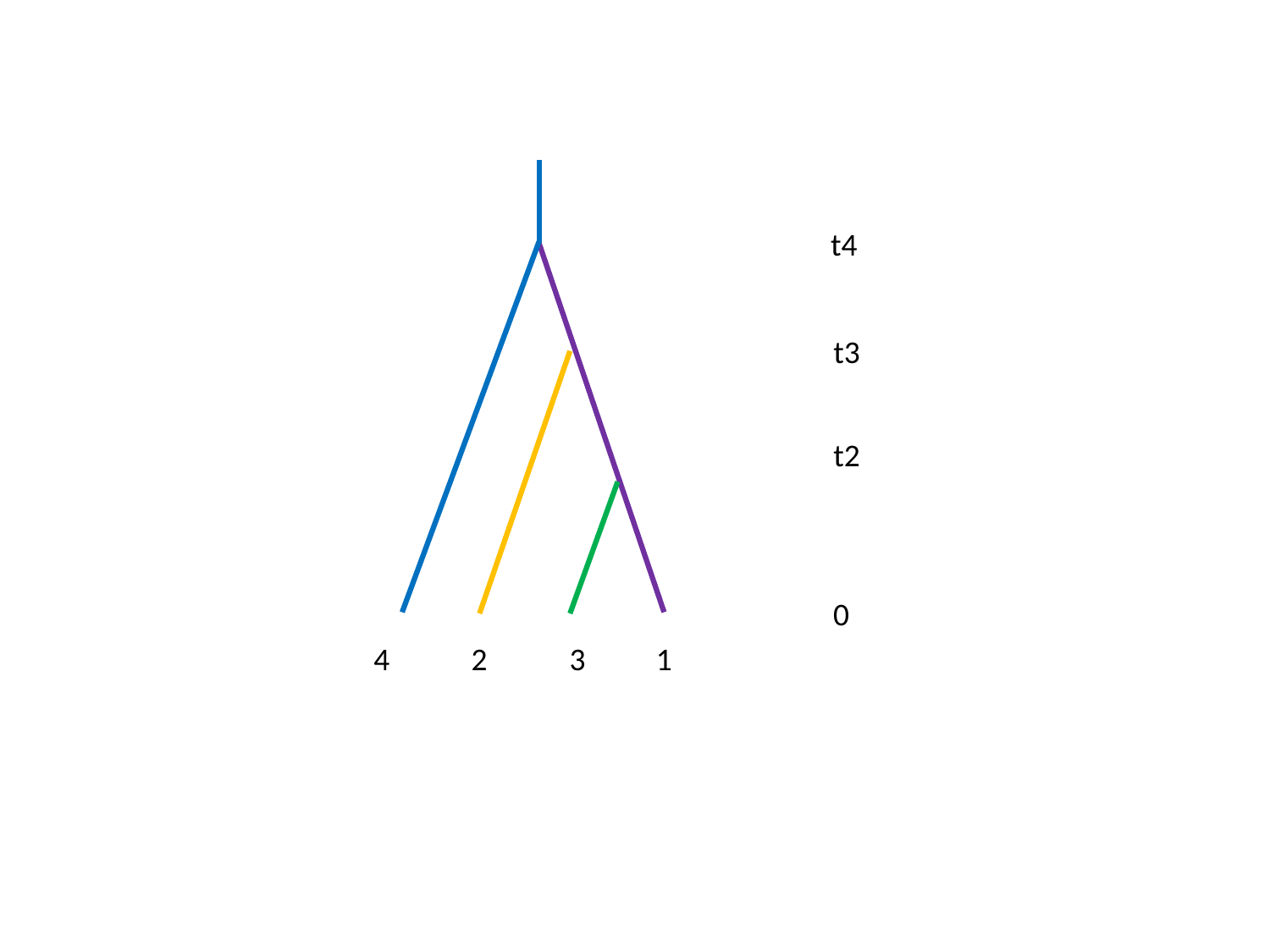

t4
t3
t2
0
4
2
3
1
